# Effect of proline rich 15-deficiency on trophoblast viability and survival

**DOI:** 10.1371/journal.pone.0174976

**Published:** 2017-04-05

**Authors:** Katherine C. Gates, Lindsey N. Goetzmann, Jeremy D. Cantlon, Kimberly M. Jeckel, Russell V. Anthony

**Affiliations:** Department of Biomedical Sciences, College of Veterinary Medicine and Biomedical Sciences, Colorado State University, Fort Collins, Colorado, United States of America; Institute of Zoology, Chinese Academy of Sciences, CHINA

## Abstract

Deviations from the normal program of gene expression during early pregnancy can lead to early embryonic loss as well as dysfunctional placentation, which can cause significant morbidity and mortality. Proline rich 15 (PRR15) is a low molecular weight nuclear protein expressed by the trophoblast during early gestation. Lentivirus-mediated knockdown of *PRR15* mRNA in ovine trophectoderm led to demise of the embryo by gestational day 15, providing compelling evidence that *PRR15* expression is critical during this precarious window of development. Our objective was to determine the effect of *PRR15* knockdown on trophoblast gene expression, proliferation, and survival. The first-trimester human trophoblast cell line, ACH-3P, was infected with control lentivirus or a lentivirus expressing a short hairpin (sh)RNA to target *PRR15* mRNA for degradation, resulting in a 68% reduction in *PRR15* mRNA. Microarray analysis of these cell lines revealed differential expression of genes related to cancer, focal adhesion, and p53 signaling. These changes included significant up-regulation of *GDF15*, a cytokine increased in pregnancies with preeclampsia. Viability and proliferation decreased in PRR15*-*deficient cells, which was consistent with down-regulation of cell cycle-related genes *CCND1* and *CDK6* and an up-regulation of *CCNG2* and *CDKN1A* in the PRR15-deficient cells. *TNFSF10*, a tumor necrosis factor superfamily member known to induce apoptosis increased significantly in the PRR15-deficient cells. Migration through a basement membrane matrix decreased and an increased population of apoptotic cells was present when treated with shRNA to target *PRR15*. These results suggest that PRR15 enhances trophoblast viability and survival during early implantation and placentation.

## Introduction

Maintenance of pregnancy in eutherian mammals requires an intricate coordination of events between the embryo and endometrium in order to develop a fully functional placenta. The period of early pregnancy when the embryo begins to attach, adhere to, and invade into the endometrium is the most precarious time for the developing embryo. In humans, it is estimated that nearly half of all conceptions are lost, with the majority of these losses occurring during early pregnancy [1,2]. Additionally, common disorders of pregnancy, such as early-onset preeclampsia and intrauterine growth restriction, originate with defective placentation during the first trimester [3]. Appropriate proliferation, differentiation, and turnover of trophoblast cells are required for normal placental development, while aberrations in the normal program of gene expression may trigger disorders of early pregnancy.

The trophectoderm is the first lineage to differentiate in the developing embryo, and is the source of the established placenta [4]. During human implantation, cytotrophoblast cells begin to differentiate into either invasive extravillous cytotrophoblasts, which invade the maternal decidua, or villous cytotrophoblasts, which fuse to form the multinucleated syncytium [5,6]. The cytotrophoblast cells underlying the syncytium continue to fuse throughout gestation as the villous surface area expands. A balance of cell turnover and renewal allows for appropriate and controlled growth of the placenta. Trophoblast apoptosis is increased in pregnancies complicated by intrauterine growth restriction (IUGR) and preeclampsia, suggesting a disruption in the normal balance of cell death and proliferation in these placentas [7]. In all mammalian species, a coordinated expression of transcription factors, cell cycle regulators, growth factors, and other genes is essential to proper placental development.

Proline rich 15 (PRR15) is a small, well-conserved nuclear protein originally identified in murine intestinal epithelium [8]. Glover and Seidel [9] independently identified *PRR15* in elongating bovine embryos by mRNA differential display analysis. In silico analysis of this cDNA predicted an open reading frame encoding a 126 amino acid protein with four putative protein kinase C (PKC) phosphorylation sites, two casein kinase II phosphorylation sites, and a nuclear targeting sequence [9]. The expression profile in the sheep conceptus during pregnancy revealed a peak in expression at day 16 of gestation [10]. This coincides with a halt in elongation of the conceptus and a period of apposition, followed by attachment to the uterine epithelium [11]. Immunohistochemistry localized PRR15 to the trophectoderm and extraembryonic endoderm of day 15 sheep conceptuses [10]. *PRR15* mRNA expression increased when trophoblast cells, both sheep (oTR) and human (ACH-3P), were cultured on Matrigel, a basement membrane matrix. During this time, cells cluster together and appear to invade into the extracellular matrix [12]. First trimester human cytotrophoblasts grown on extracellular matrix differentiate into an invasive phenotype, characterized by the same phenotypic changes observed in our trophoblast cell lines [13]. Lentivirus-mediated knockdown of *PRR15* in ovine trophectoderm at the blastocyst stage led to demise of the embryo by day 15 of gestation [10]. This provides compelling evidence that PRR15 is a critical factor during this window of development where proliferation gives way to differentiation of the trophoblast cells.

In view of the fact that *PRR15* expression increases upon induction of the invasive, more differentiated phenotype, it could be involved in the pathogenesis of placental disorders demonstrating disturbed trophoblast growth. Lentivirus-mediated delivery of shRNA provided robust evidence for the necessity of PRR15 during early embryonic development in the sheep. PRR15 does not contain any known DNA binding motifs and may not have a direct effect on gene transcription. Due to its nuclear localization, it may act as a co-activator or co-repressor of transcription or influence mRNA processing. Understanding the effect of PRR15 on trophoblast gene expression will help to illuminate the function it may play in placental development. Therefore, our objective was to determine the impact of PRR15 deficiency on trophoblast gene expression, proliferation and apoptosis.

## Materials and methods

### Immunohistochemistry

First trimester human placentas were obtained at 6 (n = 3), 8 (n = 3) or 11 (n = 1) weeks of gestation following elective pregnancy terminations from anonymous, non-smoking, non-drug using patients 18 to 28 years of age, with written consent, as per protocol 10-1623H approved by the Colorado State University Institutional Review Board. A portion of the 6- and 8-week placental samples were frozen at -80°C until used for total cellular RNA isolation (see below). The remainder of the 6- and 8-week placental samples, as well as the 11-week sample, were fixed in 4% paraformaldehyde in PBS (140 mM NaCl, 2.7 mM KCl, 10 mM Na2HPO4, and 1.8 mM KH2PO4, pH 7.3) for 1 h and then placed into 70% ethanol overnight at 4°C before paraffin embedding. Six-micrometer sections were cut from the 11-week placental sample and placed onto Superfrost/Plus slides (Thermo Fisher Scientific, Waltham, MA) and dried overnight. Slides were then deparaffinized and were rehydrated through a graded ethanol series (100%, 95%, 70%, and 50%). Sections were then bathed in 3% hydrogen peroxide for four 30-minute incubations to quench any endogenous peroxidase activity. Slides were blocked overnight in 2% goat serum in a humidified chamber at 4°C and were then incubated with primary antibody (CSU-αoPRR15–146) [10] at a 1:5000 dilution, or with primary antibody (1:5000) pre-absorbed with 8 ng of recombinant ovine PRR15 [10]. Staining was performed using the Vectastain Elite ABC kit according to the manufacturer's protocol (Vector Laboratories, Burlingame, CA) and identified with VIP substrate. In addition, 6-week placental samples were subjected to immunofluorescence for co-localization of PRR15 with human chorionic gonadotropin (CGB) or cytokeratin-7 (KRT7). Sections were incubated with CSU-αoPRR15-146 (1:5,000) along with either anti-CGB (AB9592; 1:200; Abcam, Cambridge, MA) mouse monoclonal antibody or anti-KRT7 (AB9098; 1:100; Abcam) mouse monoclonal antibody, before incubation with 1:1000 goat anti-rabbit AlexaFluor 488 (AB150077; Abcam) and goat anti-mouse AlexaFluor 594 (AB150092; Abcam).

### Cell culture and lentiviral infection

The human first trimester trophoblast cell lines ACH-3P [14], HTR-8 [15], and Swan-71 [16], as well as BeWo choriocarcinoma cells [17], were assessed for *PRR15* mRNA concentration (see below). ACH-3P cells were maintained in Ham’s F-12 medium supplemented with 10% v/v fetal bovine serum (FBS). HTR-8 cells were maintained in RPMI 1640 medium supplemented with 10% v/v FBS. Swan-71 cells were maintained in Dulbecco’s modified eagle’s medium (DMEM) supplemented with 10% v/v FBS. BeWo cells were maintained in Ham’s F12K medium supplemented with 10% v/v FBS.

ACH-3P cells were used to generate cell lines for the following experiments. The lentiviral vector pLL3.7 [18] was used to create stable cell lines by transfection as well as to generate lentivirus for infection, described below. This vector contains a multiple cloning site for introducing shRNA cassettes downstream of the mouse U6 RNA polymerase III promoter, as well as enhanced green fluorescent protein (EGFP) driven by the cytomegalovirus promoter. For both infection and transfection, the control cell lines contained the LL3.7 vector with no shRNA cassette. To target *PRR15* mRNA for degradation, a shRNA homologous to human *PRR15* was inserted into the pLL3.7 vector (5’-TGGAAATCGCTCACCAACATTTCAAGAGACTGTTGGTGAGCGATTTCCTTTTTT-3’). This vector was used previously [10] as a negative control during the *in vivo* infections of sheep conceptuses, as it contains 3-bp mismatches to the ovine *PRR15* mRNA and specifically targeted human *PRR15* mRNA rather than ovine. The transfected and infected cells are referred to as “control” or “PRR15-shRNA” from this point forward.

Lentivirus was generated as described previously [10]. Briefly, 293FT cells were grown to confluence in a 15-cm tissue culture plate in high glucose DMEM supplemented with 10% v/v FBS. For each 15-cm plate, Polyfect (180 μl, Qiagen, Valencia, CA) was added to the following lentiviral and packaging vectors in serum-free DMEM to a total volume of 675 μl: pLL3.7 lentiviral construct (8.82 μg, control LL3.7 or PRR15-shRNA), pRΔ8.74 (6.66 μg; *gag*/*pol* elements), and pMD2.G (2.70 μg; *env* elements). The Polyfect-DNA mixture was added to 293FT cells along with 15 ml complete medium. After 4–6 hours of incubation in the transfection reagent, the medium was aspirated, cells were washed in PBS, and fresh complete medium was added. Two days after transfection, cell culture supernatants were collected and ultracentrifuged over a 20% w/v sucrose cushion at 47,000x*g* for 2 hours at 4°C. After ultracentrifugation, lentiviral pellets were resuspended in Ham’s F-12 supplemented with 10% v/v FBS, and stored in aliquots at -80°C. Aliquots of lentivirus were titered as described previously [10].

ACH-3P cells were infected in three replicate experiments with either control LL3.7 or PRR15-shRNA lentivirus at a multiplicity of infection of 100 transducing units per cell in 30 mm tissue culture dishes. To create stable lines, ACH-3P cells were co-transfected with either the control LL3.7 or PRR15-shRNA vector and pcDNA3.1 (Invitrogen, Carlsbad, CA) in a 20:1 ratio using Superfect (Qiagen) following the manufacturer’s protocol. The pcDNA3.1 vector contains a neomycin-resistance gene, allowing for selection of transfected cells. Transfected cells were selected by treatment with 400 μg/ml neomycin (G418) for three weeks. The concentration of *PRR15* mRNA in transfected and infected cells was assessed by quantitative real-time reverse transcriptase PCR (qPCR) and Western blot analysis.

### RNA isolation and microarray analysis

Total cellular RNA was isolated from placental tissue and cells using the RNeasy Mini Kit (Qiagen) according to the manufacturer’s protocol. RNA quality, measured by the 260/280 nm absorbance ratio, and concentration were assessed using a NanoDrop 1000 Spectrophotometer (Thermo Fisher Scientific). Samples were stored at -80°C until use. RNA from three replicate infections with control and PRR15-shRNA lentivirus was submitted to the Colorado State University Genomics and Proteomics Core for processing and hybridization to the GeneChip Human Genome U133 Plus 2.0 Array (Affymetrix, Santa Clara, CA). The raw data intensity files were read into ArrayTrack for analysis (http://www.fda.gov/ScienceResearch/BioinformaticsTools/Arraytrack/default.htm). Data were normalized by scaling to the geometric mean of the intensities of each chip. Genes that were flagged in more than three samples due to intensities too low to be reliable were excluded from the analysis. Expression data were deposited into the Gene Expression Omnibus (GSE41459). Control and PRR15-shRNA groups were compared by Welch’s t-test on log base 2 expression values (S1 Table presents differentially expressed genes with *p*<0.05 in Welch’s t-test, with ≥1.3-fold change). Pathway analysis on differentially expressed genes (*p*<0.05, ≥1.3-fold change) was conducted using the Kyoto Encyclopedia of Genes and Genomes (http://www.genome.jp/kegg/) pathway maps [19]. Fisher’s exact test was used to determine pathways that were significantly altered (*p*<0.05) by *PRR15* deficiency.

### Quantitative real-time RT-PCR

cDNA was generated from 1 μg of total cellular RNA by reverse transcription at 55°C for 50 min using oligo(dT) primers (Superscript III; Invitrogen), following the manufacturer’s protocol. Each cDNA sample was treated with 5 units of RNase H (Fermentas, Burlington, Canada) for 20 min at 37°C. Quantitative real-time RT-PCR (qPCR) was performed as described previously [10] except the samples were analyzed on a Lightcycler 480 (Roche Applied Science, Indianapolis, IN) in a 10 μl reaction volume. All primer sets were designed using Oligo software (Molecular Biology Insights, Cascade, CO) to amplify an intron-spanning product; forward and reverse primers for each gene are shown in S2 Table, along with conditions for qPCR. A PCR product for each gene was generated using cDNA from ACH-3P cells as a template and cloned into the PCR-Script Amp SK(+) vector (Agilent Technologies, Santa Clara, CA). Each PCR product was sequenced to verify amplification of the correct mRNA (Colorado State University Proteomics and Metabolomics Facility). A standard curve was generated from 1x10^2^ to 1x10^-6^ pg using a PCR product amplified from the sequenced plasmid for each gene and used to measure amplification efficiency. The starting quantity (pg) of each mRNA was normalized to the starting quantity (pg) of ribosomal protein S15 (*RPS15*), after verifying that the *RPS15* mRNA concentration did not change with treatment (*p*>0.50). Control and PRR15-shRNA treatments were compared by Students t-test, with *p*<0.05 selected as significant.

### Nuclear protein isolation and Western blot

Control and PRR15-deficient ACH-3P cells were dislodged from sub-confluent culture dishes using trypsin (0.25% w/v with 0.5 mM EDTA), washed in PBS, and pelleted. Nuclear protein was extracted using a modified Dignam method [20]. Cells were resuspended in three volumes of hypotonic buffer (10 mM HEPES, 1.5 mM MgCl2, 10 mM KCl, 0.5 mM DTT, 0.2 mM PMSF) and allowed to swell on ice for 10 minutes. The cells were homogenized in a Dounce homogenizer and nuclei were pelleted by centrifugation at 3300x*g* for 30 minutes. The nuclear pellet was resuspended in ½ volume low salt buffer (0.15 M NaCl, 0.1 mM EDTA, 20 mM TrisHCl, 0.5 mM DTT, 0.2 mM PMSF), followed by slowly adding ½ volume of high salt buffer (same as low salt with 1 M NaCl). Nuclei were extracted by gentle shaking on ice for 30 minutes, and then pelleted by centrifugation at 25,000x*g* for 30 minutes. The nuclear extract was dialyzed overnight in 10,000 molecular weight cutoff (MWCO) Slide-A-Lyzer dialysis cassettes (Thermo Fisher Scientific) against dialysis buffer (20 mM HEPES, 0.2 mM EDTA, 0.5 mM DTT, and 0.2 mM PMSF). Following dialysis, the extract was concentrated by centrifugation over a 3,000 MWCO Amicon centrifugal filter (EMD Millipore, Billerica, MA) and protein concentration was determined by Bradford assay. Glycerol (20% v/v) was added as a cryoprotectant prior to aliquoting and storage at -80°C until use.

Thirty μg aliquots of protein reduced in β-mercaptoethanol were electrophoresed through 4–12% w/v bis-tris denaturing gels in MOPS-SDS running buffer (Invitrogen). After electrophoresis, proteins were transferred to nitrocellulose membranes, and the membranes blocked with non-fat dry milk TBST buffer (5% w/v NFDM, 10 mM Tris-HCl, 150 mM NaCl, and 0.1% v/v Tween 20, pH 8.0) at 37°C overnight. Membranes were then incubated with rabbit anti-PRR15 (1:1000) or rabbit anti-β-actin (1:1,000) at 4°C overnight, then rinsed in TBS (10 mM Tris-HCl pH 8.0, 150 mM NaCl, pH 8.0). Subsequently, membranes were incubated with anti-rabbit IgG (1:5000) horseradish peroxidase-conjugated secondary antibody overnight and rinsed in TBS. Following incubation in ECL Prime reagent (GE Healthcare, Pittsburgh, PA), the membranes were imaged with a ChemiDoc XRS (BioRad, Hercules, CA).

### Viability and proliferation assays

Stably transfected ACH-3P cells were plated in a 96-well plate with 5000 cells per well and three replicates per treatment. Cell viability was measured using the Cell Counting Kit 8 (Enzo Life Sciences, Farmingdale, NY). Ten μl of WST-8 [2-(2-methoxy-4-nitrophenyl)-3-(4-nitrophenyl)-5-(2,4-disulfophenyl)-2H-tetrazolium, monosodium salt] was added to each well and incubated for 3 hours, then absorbance at 450 nm was measured using a BioRad Model 680 Microplate Reader. Measurements were made 3, 24, 48, and 72 hours after plating cells and the percent change relative to the 3 hour measurement assessed. Concurrently, BrdU uptake was measured by ELISA, following the manufacturer’s protocol (Calbiochem, Darmstadt, Germany). Briefly, 5000 cells were plated in a 96-well plate the day prior to labeling, with three replicates per group. BrdU label (diluted 1:10,000) was added to media for 20 hours prior to ELISA. Cell media was removed and cells were fixed in provided fixative/denaturing solution for 30 minutes at room temperature. Cells were incubated in anti-BrdU antibody diluted 1:100 in antibody dilution buffer for one hour, washed three times in wash buffer, and then incubated with goat anti-mouse IgG HRP conjugate diluted 1:1000 in conjugate diluent for 30 minutes. The plate was washed three times, and then 100 μl substrate solution (tetra-methylbenzidine solution) was added for 15 minutes in the dark, followed by stop solution (2.5N H_2_SO_4_). Absorbance was measured on a spectrophotometric microplate reader (BioRad) at dual wavelengths of 450–595 nm. Absorbances in control and PRR15-shRNA cells were compared by Student’s t-test, with *p*<0.05 considered statistically significant.

### Caspase assays

Caspase 3/7 and 8 activity was measured using the Caspase-Glo Reagent (Promega, Madison, WI) in stably transfected ACH-3P cells following the manufacturer’s protocol. Briefly, cells (30,000 per well for Caspase 3/7 and 60,000 per well for Caspase 8) were plated in triplicate in a white-walled clear-bottom 96-well plate (Corning, Tewksbury, MA). Caspase-Glo Reagent was added and incubated at room temperature for 30 minutes. Luminescence was measured on a BioTek Microplate Reader (Winooski, VT) with integration for 10 seconds. The amount of protein in each well was quantified by a Bradford assay, and used to normalize luminescence values. Groups were compared by Student’s t-test, with *p*<0.05 considered statistically significant.

### Flow cytometry for annexin V

The FlowCellect Annexin Red Kit (EMD Millipore) was used to quantify apoptosis in stably transfected ACH-3P cells. Cells were collected by detaching with EDTA (15mM in PBS, pH 7.4) and resuspending in 1X Assay Buffer HSC. Annexin V CF647 Working Solution was added to each sample and incubated for 15 minutes in a 37°C CO_2_ incubator. Cells were washed in 1X Assay Buffer, and then incubated with 7-AAD reagent in the dark for 5 minutes. Samples were analyzed by flow cytometry on a MoFlo flow cytometer (Dako, Carpinteria, CA) at the Colorado State University Proteomics and Metabolomics Facility. The 7-AAD signal was measured on a detector with a 630/30 Band pass filter and the CF647 Annexin V signal was measured on a detector with a 670/20 Band pass filter, with compensation used between the two dyes. Data were analyzed using Summit Software (Dako). Cell counts for control versus PRR15-shRNA were compared by a Student’s t-test, with *p*<0.05 considered statistically significant.

### Matrigel invasion assay

ACH-3P and BeWo cells, infected with the Control or PRR15-shRNA virus, were grown to 70% confluency in 10 cm culture plates, then fluorescently labeled in 15 μM CellTracker ^™^ Green CMFDA (5-chloromethylfluorescein diacetate) Dye (Life Technologies, Carlsbad, CA), following manufacturer’s instructions. After incubation, cells were washed and resuspended in complete DMEM/F-12 (1:1) Nutrient Mixture F-12 Ham’s 1X medium, without phenol red, (HyClone Laboratories, Inc., Logan, UT) supplemented with 10% v/v FBS and 1% v/v antibiotic/antimycotic at a concentration of 50,000 cells/ml. Cells were then added to the top chamber (25,000 cells/well) of 8 μm pore BioCoat Tumor Invasion System 24-Multiwell Insert Plates (BD Biosciences, San Jose, CA). At time 0, and again at 4, 24 and 48 hours after plating, fluorescence was measured in both the top and bottom chambers using a BioTek Synergy 2 Multi-Mode Plate Reader (BioTek), with excitation at 485 nm and emission at 528 nm. After normalizing to time 0, the invasion index was calculated using the formula: (Bottom/(Top+Bottom))*100. Resulting invasion index data for each time-point, within ACH-3P and BeWo control and PRR15-shRNA cell lines, were compared by a Student’s t-test, with *p*<0.05 considered statistically significant.

## Results

### PRR15 is expressed in first-trimester human placenta

**Fig 1:**
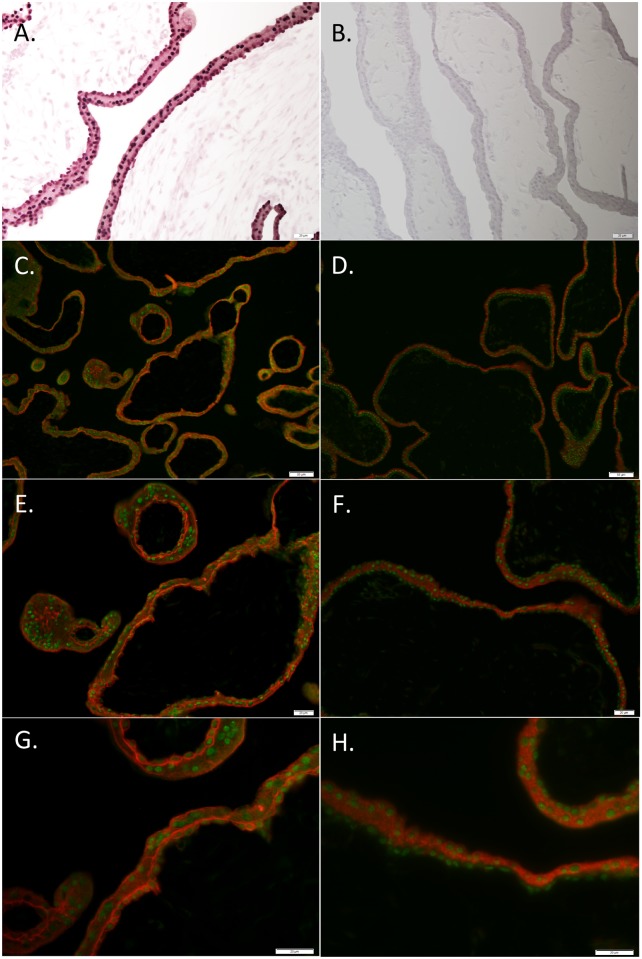
Immunohistochemical localization of PRR15 in 6- and 11-week human placenta. (A) An 11-week placenta sample was stained with rabbit anti-PRR15 antibody, or (B) with the rabbit anti-PRR15 antibody that was preadsorbed with recombinant PRR15 as a negative control. Six-week placenta was used to co-localize PRR15 (green fluorescence) with KRT7 (red fluorescence; trophoblast marker; C, E, G) and with CGB (red fluorescence; syncytiotrophoblast marker; D, F, H). Collectively, these data verify that PRR15 is predominantly localized to the nucleus of both cytotrophoblasts and syncytiotrophoblasts of the human placenta. Magnification bars represent: 20 μm for A, B, E, F, G and H; 50 μm for C and D.

**Fig 2:**
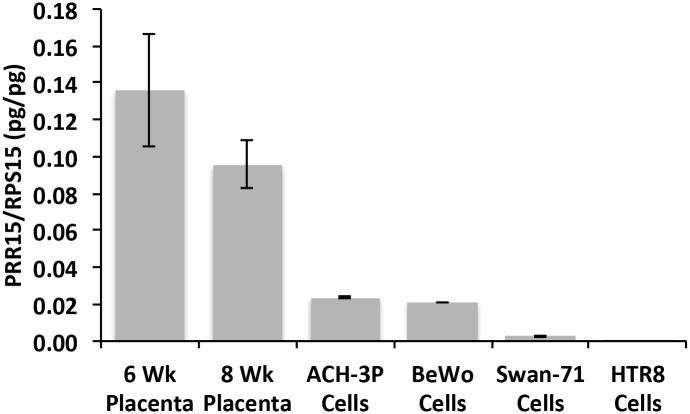
qPCR for *PRR15* in first-trimester human placenta and first-trimester human trophoblast cell lines. *PRR15* mRNA concentrations were normalized to ribosomal protein S15 (*RPS15*) in human placenta at 6 and 8 weeks of gestation, and human trophoblast-derived cell lines ACH-3P, BeWo, Swan-71, and HTR-8.

Immunohistochemistry of 6- and 11-week human placenta tissue localized PRR15 predominantly to the nuclei of both cytotrophoblast cells and the syncytiotrophoblast layer (Fig 1). Immunofluorescent co-localization of PRR15 with KRT7 (Fig 1C, 1E and 1G) verified its cytotrophoblast expression, wherease co-localization with CGB (Fig 1D, 1F and 1H) verified that PRR15 was also expressed in the syncytiotrophoblast layer of the placenta. We then tested the mRNA concentrations of first-trimester human placental tissue and compared this to BeWo cells (choriocarcinoma cell line) and three first-trimester human trophoblast cell lines: ACH-3P, Swan-71, and HTR-8. Both 6- and 8-week placental samples demonstrated significant mRNA expression (Fig 2). ACH-3P and BeWo cells had the highest relative mRNA concentration of the four human cell lines, thus ACH-3P cells were used for subsequent experiments.

### PRR15-deficiency alters trophoblast gene expression

**Fig 3:**
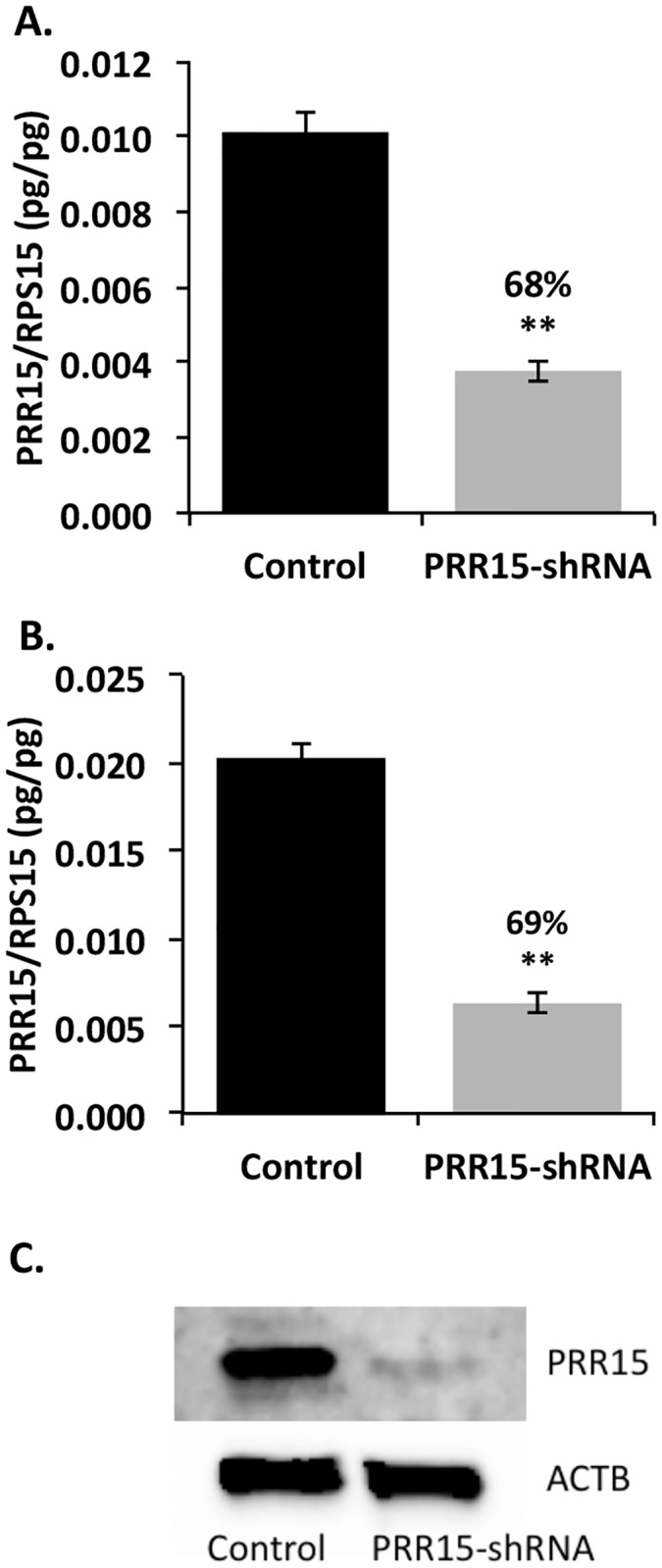
*PRR15* qPCR and Western blot analysis in ACH-3P cells transfected and infected with PRR15*-*shRNA. *PRR15* mRNA concentration decreased significantly in the presence of a *PRR15*-specific shRNA. qPCR for *PRR15* normalized to ribosomal protein S15 (*RPS15*) in (A) cells infected with control or PRR15-shRNA lentivirus or (B) cells transfected with vectors with or without shRNA. (C) Western blot on nuclear extract from transfected cells demonstrating a 92.5% knockdown of PRR15 normalized to β-actin (ACTB). Control indicates cells infected or transfected with control lentilox vector; PRR15-shRNA indicates cells infected or transfected with virus/vector containing *PRR15*-targeting shRNA. ** indicates *p*<0.01 in Student’s t-test.

Transfection and infection of ACH-3P cells with a shRNA to target *PRR15* mRNA for degradation resulted in a comparable decrease in *PRR15* mRNA concentrations for both methods. Lentivirus infection led to a 68% decrease in *PRR15* mRNA (p<0.01, Fig 3A) as measured by qPCR, with a 62% reduction (p<0.06) in the microarray analysis. To guard against integration effects we also assessed *PRR15* mRNA in stably transfected cells, which exhibited a 69% reduction (p<0.01, Fig 3B). Western blot analysis of nuclear protein derived from the stably transfected cells demonstrated a 92.5% decrease in PRR15 within shRNA-expressing cells as compared to the control cells (Fig 3C). In the microarray comparison of control to PRR15-shRNA cells, 1375 genes were differentially expressed with a p<0.05 and greater than 1.3-fold change (S1 Table). Pathway analysis was conducted on these differentially expressed genes using KEGG pathway maps. From the 1375 input genes, 285 genes were found in 155 total pathway maps. Fisher’s exact test revealed significant changes in pathways related to proliferation, cancer, and focal adhesion. Specifically, colorectal cancer, p53 signaling, and focal adhesion were the pathways most affected by *PRR15* deficiency.

From the microarray analysis, we selected genes for validation with qPCR that had the most dramatic changes in the *PRR15*-deficient cells or had known cellular functions potentially related to trophoblast development. Twenty-one genes were selected for validation, 18 (86%) of which gave results consistent with the microarray study. The remaining genes expressed the same trend of up- or down-regulation as in the microarray analysis, but were not statistically significant (p≥0.05) in the qPCR results (Table 1). The genes that were validated by qPCR can be divided into several functional groups, with some genes present in more than one category: regulation of the cell cycle (*CCND1*, *CCNG2*, *CDK6*, *CDNK1A*), cell differentiation (*JAG1*, *OVOL2*, *TWIST1*), cell survival/apoptosis (*CRYAB*, *GDF15*, *MXD1*, *MYC*, *TNFSF10*), cell migration and/or invasion (*CCDC88A*, *PTEN*, *PXN*, *TFPI2*, *TWIST1*), insulin-like growth factor (IGF) signaling (*IGF1R*, *IGFBP3*, *PTEN*, *SOCS2*), and placental function (*LIFR*, *OVOL2*).

**Table 1:** Differentially expressed genes from *PRR15* microarray and qPCR validation*.

Gene Symbol	Gene Name	Microarray		qRT-PCR	
		Fold	p	Fold	p
**Cell Cycle Regulation**					
*CCND1*	cyclin D1	-2.1	0.055	-2.5	0.033
		-1.4	0.052		
*CCNG2*	cyclin G2	+2.4	0.012	+2.1	0.002
		+3.3	0.003		
		+2.8	0.036		
*CDK6*	cyclin-dependent kinase 6	-1.9	0.016	-2.7	0.050
		-2.7	0.012		
		-1.9	0.015		
		-1.7	0.004		
*CDKN1A*	cyclin-dependent kinase inhibitor 1A (p21)	+1.9	0.014	+1.7	0.030
**Cell Differentiation**					
*JAG1*	jagged 1	+2.2	0.008	+2.0	0.001
		+2.3	0.010		
*OVOL2*	ovo-like 2	-2.4	0.030	-2.8	0.034
*TWIST1*	twist homolog 1	-1.8	0.025	-1.5	0.206
**Cell Survival or Apoptosis**					
*CRYAB*	crystallin α B	+4.6	0.009	+3.8	0.002
*GDF15*	growth/differentiation factor 15	+3.6	0.007	+49.0	0.0001
*MXD1*	MAX dimerization protein 1	+2.0	0.007	+1.7	0.025
		+2.2	0.010		
*MYC*	v-myc myelocytomatosis viral oncogene homolog	-1.6	0.016	-2.4	0.003
*TNFSF10*	tumor necrosis factor superfamily member 10 (TRAIL)	+2.8	0.069	+8.1	0.011
		+2.8	0.007		
		+3.0	0.001		
**Cell Migration or Invasion**					
*CCDC88A*	coiled-coil domain containing 88A (girdin)	-3.3	0.043	-3.1	0.047
*PTEN*	phosphatase and tensin homolog	-1.3	0.088	-2.6	0.054
		-1.2	0.002		
		-1.5	0.008		
		-1.4	0.023		
*PXN*	paxillin	-2.1	0.097	-1.4	0.017
*TFPI2*	tissue factor pathway inhibitor 2	-1.9	0.240	-10.5	0.0002
		-39.0	0.002		
**IGF Signaling**					
*IGF1R*	insulin-like growth factor 1 receptor	-1.4	0.300	-1.6	0.185
		-1.4	0.018		
		-2.2	0.005		
*IGFBP3*	insulin-like growth factor binding factor 3	-2.1	0.008	-2.0	0.019
*SOCS2*	suppressor of cytokine signaling 2	-2.9	0.008	-2.8	0.009
**Placental Function**					
*LIFR*	leukemia inhibitory factor receptor	-1.5	0.048	-1.7	0.033
		-2.2	0.008		
		-1.6	0.042		
		-2.3	0.025		

* For each gene, all probe-sets from the microarray analysis are shown.

### Viability decreases and apoptosis increases in PRR15-deficient cells

**Fig 4:**
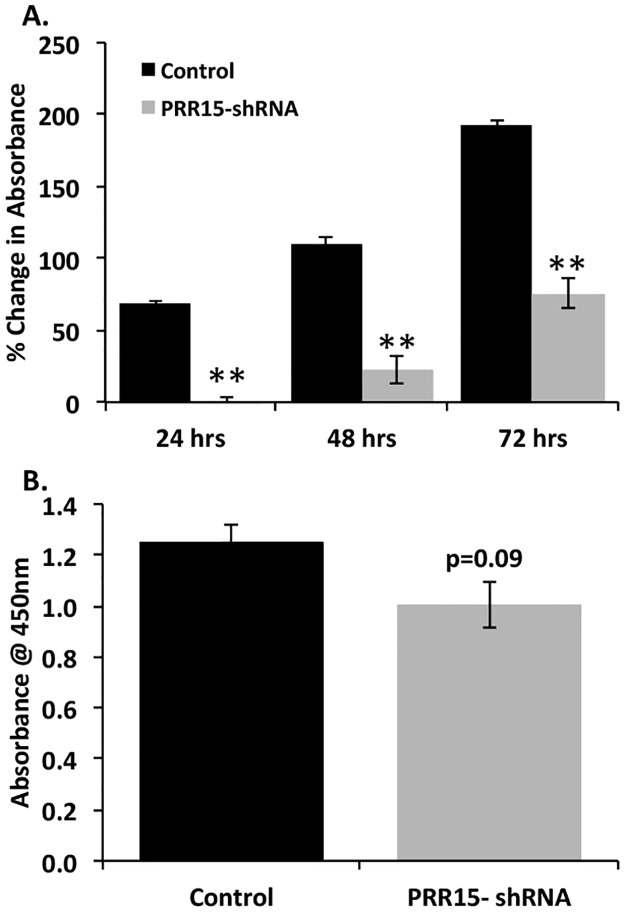
Viability decreases in PRR15-deficient ACH-3P cells. (A) CCK-8 assay presenting change in absorbance over time in culture of stably transfected ACH-3P cells. (B) ELISA for BrdU uptake in stably transfected ACH-3P cells. Control indicates cells transfected with control LL3.7 vector; PRR15-shRNA indicates cells transfected with vector containing *PRR15*-targeting shRNA. ** indicates *p*<0.01.

Because the microarray revealed differentially expressed genes in pathways related to proliferation and cell survival, we opted to measure cell viability, proliferation and apoptosis in PRR15-deficient trophoblast cells. ACH-3P cells transfected with the shRNA-expressing vector to target *PRR15* mRNA for degradation had significantly decreased viability based on the CCK-8 assay (Fig 4A). When DNA synthesis was measured by the uptake of BrdU, the decrease in the PRR15-shRNA cells only tended to be statistically significant (p = 0.09), although the same trend toward decreased cell proliferation in the PRR15-shRNA cells was observed (Fig 4B).

**Fig 5:**
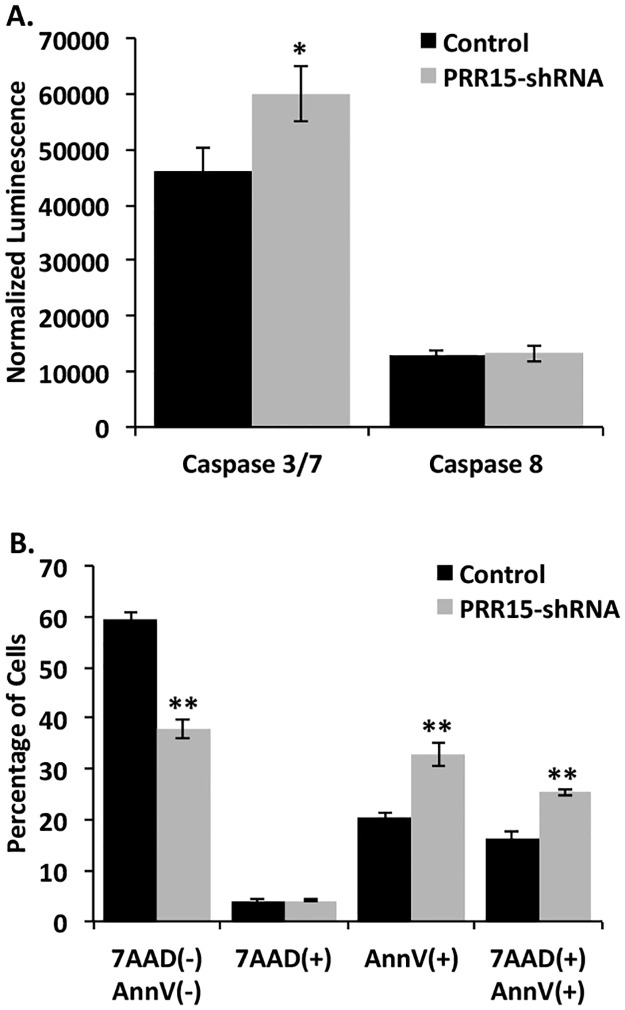
Apoptosis increases in PRR15-deficient ACH-3P cells. (A) Caspase 3/7 and 8 activity was measured using Caspase-Glo Reagent. Luminescence values were normalized to protein concentration in each well. * indicates *p*<0.05 in Student’s t-test. (B) Annexin V staining was quantified by flow cytometry. 7-AAD(-), AnnV(-) indicates cells that were not positive for either stain; 7-AAD(+) indicates necrotic cells; AnnV(+) indicates early apoptotic cells; 7-AAD(+), AnnV(+) indicates late apoptotic and necrotic cells. * indicates *p*<0.05, and ** indicates *p*<0.01 in Student’s t-test.

Apoptosis was measured by the activation of caspases involved in the apoptotic cascade. Caspase 3/7 activity was significantly increased in the PRR15-shRNA cells, while caspase 8 activity was unchanged (Fig 5A). We confirmed the changes in apoptosis by flow cytometric analysis of annexin V staining. In order to distinguish apoptotic from dead cells, 7-AAD was used which binds to nucleic acids in late apoptotic or necrotic cells. The percentage of cells that did not absorb either the annexin V or 7-AAD stains decreased significantly in the PRR15-shRNA cells, while early apoptotic and late apoptotic/necrotic cells increased (Fig 5B). These results demonstrate an increased tendency to undergo apoptosis when *PRR15* mRNA concentration is diminished in ACH-3P cells.

### Trophoblast invasion may be impaired by PRR-15 deficiency

**Fig 6:**
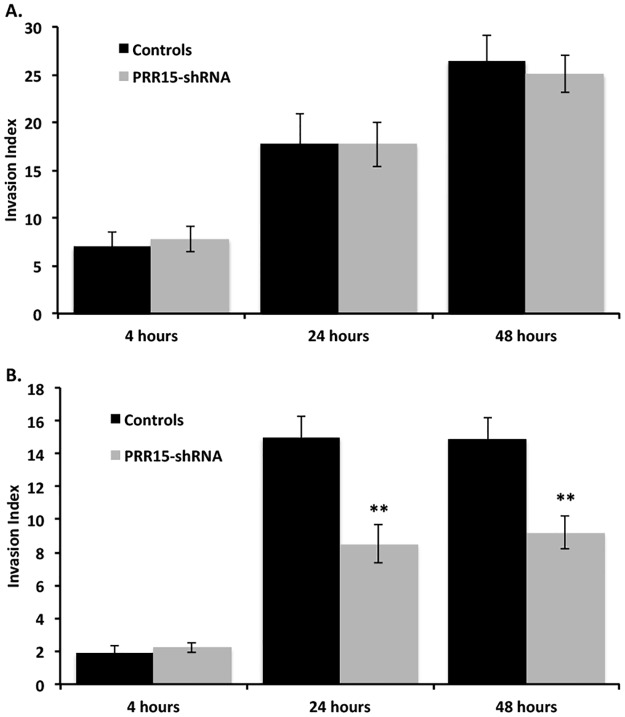
Invasion indices for control and PRR15-deficient ACH-3P and BeWo cells. Invasion was assessed through Matrigel^®^-coated invasion chamber inserts (8 μm) for ACH-3P (A) and BeWo (B) cells. Invasion index was normalized to time 0 for each well. ** indicates *p*<0.01 in Student’s t-test.

Since the expression of genes involved in cell migration and/or invasion was altered in PRR15-deficient cells, we assessed the impact of PRR15 deficiency on ACH-3P invasion. In contrast to the gene expression changes observed, PRR15 deficiency did not alter the invasion index of ACH-3P cells over a 48 hour period (Fig 6A). To determine if the lack of effect on ACH-3P invasion was representative of all trophoblast cells, we infected BeWo cells with our control and PRR15-shRNA virus, since BeWo cells had equivalent expression of *PRR15* mRNA (Fig 2). With BeWo cells, PRR15 deficiency did significantly reduce the invasiveness of these cells, as monitored at 24 and 48 hours (Fig 6B).

## Discussion

The nuclear protein PRR15 is expressed in the first-trimester human placenta tissue and trophoblast-derived cell lines, suggesting it may play a role in early placentation. Diminished expression of *PRR15* in ACH-3P cells led to significant alterations in the trophoblast transcriptome. Differential expression of genes related to proliferation and cell cycle regulation revealed a reduction in pro-proliferative genes (*CCND1*, *CDK6*, *JAG1*, *MYC*, *TWIST1*) and an increase in anti-proliferative genes (*CCNG2*, *CDKN1A*, *MXD1*) in the PRR15-deficient ACH-3P cells. The insulin-like growth factor (IGF) signaling axis also plays a role in cell proliferation: binding of IGF1 and 2 to the IGF Type 1 receptor (IGF1R) promotes cell growth and proliferation [21]. Both *IGF1R* and *IGFBP3* were down-regulated in the PRR15-shRNA cells (1.6-fold, p = 0.185, 2.0-fold, p = 0.019, respectively), suggesting a decrease in IGF-axis activity and a decrease in growth and proliferation in the PRR15-deficient cells. Conversely, suppressor of cytokine signaling 2 (*SOCS2*), a negative regulator of the IGF1 signaling pathway [22], was also reduced in the PRR15-shRNA cells (1.4-fold, p = 0.009), which counteracts the changes observed in *IGF1R* and *IGFBP3*. Furthermore, phosphatase and tensin homolog (*PTEN*), a well-described tumor suppressor, was significantly down-regulated in the PRR15-deficient cells. PTEN has been connected to numerous cellular functions including controlling cell migration through its dephosphorylation of phosphatidylinositol-3,4,5-trisphosphate (PIP3) [23]. The reduction of *PTEN* in the PRR15-deficient cells may affect cell migration in these cells rather than decreasing proliferation. Despite a few discordant results, the majority of qPCR validated genes suggested that trophoblast cell proliferation would be reduced in the PRR15-shRNA cells as compared to the controls.

The cell viability and proliferation assays supported diminished proliferative activity in the PRR15-deficient cells. The CCK-8 assay measures cell viability through the reduction of a tetrazolium salt by cellular dehydrogenases to a yellow-colored dye. The decreased absorbance observed in the PRR15-deficient cells may be due to a reduction in cellular proliferation or an increase in apoptosis, or a combination of these phenotypes. The BrdU assay measures DNA synthesis and though it showed a decrease in the PRR15-shRNA cells, the difference was not as dramatic as that observed in the CCK-8 assay. This suggests that the PRR15-deficient cells may be more susceptible to apoptosis in addition to somewhat diminished proliferative capacity.

Both the caspase assay and the annexin V staining revealed increased apoptosis in the PRR15-shRNA cells. Caspases, or cysteine-dependent aspartate-specific proteases, are enzymes that aid in the execution of programmed cell death or apoptosis. Caspase 8 is known as an “initiator” caspase in the extrinsic pathway of apoptosis, while Caspases 3/7 are “executioner” caspases activated by both the intrinsic and extrinsic apoptotic pathways [24]. Increased Caspase 3/7 activity suggests that the PRR15-deficient cells are more susceptible to apoptosis through the intrinsic pathway. The annexin V staining confirmed an increased susceptibility to apoptosis upon diminished *PRR15* expression in ACH-3P cells.

Apoptosis, or programmed cell death, is a necessary process in normal placental development as trophoblast cells undergo constant turnover and renewal. However, apoptosis increases in placentas suffering from complications such as preeclampsia, intrauterine growth restriction, and hydatidiform moles [25]. In relation to the cell cycle and proliferation, evidence shows that CCND1 is decreased in placentas from IUGR and preeclampsia complicated with IUGR [26], while CDKN1A is increased in IUGR placentas [27]. During normal trophoblast development, PRR15 may protect cells from apoptosis and promote trophoblast cell proliferation and survival.

In the microarray analysis, we observed differential expression of several genes related to apoptosis. *TNFSF10*, also known as *TRAIL*, is a death receptor ligand known to induce apoptosis in transformed and tumor cells [28]; *TFNSF10* was up-regulated in PRR15-deficient cells (8.1-fold, p = 0.011). This ligand could signal to the trophoblast cells themselves or to endometrial cells *in vivo*. In normal placental development, PRR15 likely protects cells from apoptosis and enhances cell survival, aiding in proper remodeling and formation of the placenta. In contrast to *TNFSF10*, *CRYAB*, a small heat shock protein that may protect cells from apoptosis [29,30], was increased 3.8-fold (*p* = 0.002) in the PRR15-shRNA cells. However, recent studies show that CRYAB interacts directly with p53 and is required for p53-dependent apoptosis [31], and its anti-apoptotic function is affected by its phosphorylation status [32]. Furthermore, the role of increased *CRYAB* in PRR15-deficient cells may be related to cellular functions other than apoptosis, such as acting as a chaperone for vascular endothelial growth factor A (VEGFA) during angiogenesis, a process critical for early placentation [30]. The control of cell cycle progression and cell survival is maintained through a delicate balance of a plethora of factors; these data suggest that PRR15-deficiency shifts the balance toward decreased proliferation and increased susceptibility to apoptosis.

Differentially expressed genes from the microarray analysis with known functions in implantation or placentation included *LIFR* and *OVOL2*. Endometrial expression of leukemia inhibitory factor (LIF) is required for implantation in mice [33] and is decreased in women with unexplained infertility and recurrent pregnancy loss [34,35]. Its receptor, LIFR, increases significantly during the period of conceptus elongation in pigs [36]. LIF promotes proliferation of trophoblast cells in culture and invasiveness of JEG3 cells [37,38]. OVOL2 knockout mice exhibit impaired placental labyrinth development and embryonic mortality by day 12.5 of gestation [39]. Down-regulation of both *LIFR* (1.3-fold, p = 0.033) and *OVOL2* (2.8-fold, p = 0.034) in the PRR15-shRNA cells may have contributed to the embryonic loss observed in sheep when PRR15 was depleted *in vivo* [10].

Growth-differentiation factor 15 (GDF15) is a non-canonical member of the transforming growth factor β superfamily of cytokines that is significantly up-regulated during pregnancy [40]. GDF15 peaks in circulation at 12–14 weeks gestation, and again at 33–35 weeks at approximately double the initial concentrations [40]. It is expressed primarily in villous and extravillous cytotrophoblast as well as decidual stroma, but not in the syncytiotrophoblast [41,42]. During the first trimester, Tong *et al*. demonstrated decreased concentrations of GDF15 in maternal serum in pregnancies that ended in miscarriage [43,44]. Furthermore, placental *GDF15* mRNA concentrations were elevated in preeclampsia when compared to control samples from term placentas; this elevation was also observed in maternal and fetal circulation [45,46]. However, Marjono et al. [39] detected no significant differences in serum concentrations of GDF15 associated with either labor or preeclampsia. The discrepancy may be a result of how the authors defined preeclampsia in these studies or the very limited sample size in the study by Marjono *et al*. [40]. Treatment of HTR-8 cells with GDF15 resulted in reduced proliferation and increased apoptosis as GDF15 concentrations increased [45]. This parallels the phenotypic changes observed when we diminish PRR15 in ACH-3P cells, where *GDF15* expression increased nearly 50-fold. The function of GDF15 in early implantation and placentation is not known, though the significant up-regulation observed in the PRR15-shRNA cells may infer a contribution to pregnancy failure when *PRR15* mRNA was targeted for degradation in ovine trophectoderm [10]. Moreover, it may act as a secreted signal of placental dysfunction during early implantation.

The original studies on PRR15 using in situ hybridization suggested that the mRNA was present primarily in post-mitotic cells [8,47]. A recent study demonstrated that stimulation of proliferation of rat pancreatic islet β and acinar cells led to significant down-regulation of *PRR15* mRNA [48]. These results suggest that PRR15 is more abundantly expressed in non-proliferative or post-mitotic cells. However, it has been identified in multiple cancer cells, including murine gastrointestinal tumors and advanced stage human colorectal cancer cells [49], in addition to being strongly co-expressed with the estrogen receptor in breast cancer datasets [50]. We [10] previously demonstrated the necessity of PRR15 for conceptus elongation and survival in sheep, and in the current experiments, PRR15 deficiency resulted in decreased cell survival of human trophoblast cells. Recent work by Yu *et al*. [51] might shed light on this apparent dichotomy in the cell phenotype in which PRR15 is expressed. They identified *PRR15* RNA in endodermal and extraembryonic (placenta) derived tissues, but not in ectodermal or mesodermal tissues [51], and *PRR15* transcription was tied to methylation of CpG islands (CGI) 3’ of the PRR15 gene. In the pancreas, clonal bisulfite sequencing revealed both totally unmethylated as well as heavily methylated DNA molecules, indicating cell-type specific methylation. The positive relationship between *PRR15* 3’ CGI methylation and its expression was also demonstrated with differentiating human embryonic stem cells (hESC) [51]. The undifferentiated hESCs exhibited low *PRR15* expression and 3’ CGI methylation, but during differentiation to fibroblasts, both increased concomitantly initially, but over time *PRR15* mRNA diminished while 3’ CGI methylation was maintained. It appears that *PRR15* expression appears to be cell-type specific and may well be important at least during early stages of differentiation.

If the expression of *PRR15* is tied to cell differentiation, this might also explain the differing response in the invasion index associated with PRR15-deficiency in ACH-3P and BeWo cells. Due to their origin, ACH-3P cells are a more homogenous cytotrophoblast phenotype, whereas BeWo cells are heterogenous with both cytotrophoblast and syncytiotrophoblast phenotypes. If the degree of cytotrophoblast differentiation differs between these two cell lines, this could have led to the varying effect of PRR15 deficiency on basement membrane-induced differentiation into an invasive phenotype. Previous studies [52,53] demonstrate that mRNA and microRNA (miRNA) expression differs between primary cytotrophoblasts, primary extravillous trophoblasts and a variety of immortalized trophoblast-derived cells (e.g., ACH-3P, BeWo, HTR8, etc), such that it is not surprising to see variable responses in a given experimental endpoint amongst various trophoblast cells or trophoblast-derived cells.

While this study provides evidence that PRR15 affects gene expression in human trophoblast cells and enhances trophoblast survival, it does not directly assess the mechanism of PRR15 function. PRR15 may function through a variety of mechanisms in order to directly affect gene expression. Immunohistochemistry and the conserved nuclear localization signal suggest that PRR15 is primarily nuclear, although it lacks a putative DNA- or RNA-binding motif [10]. It may bind to other transcription factors to suppress or activate transcription of other genes, or its effects could be post-transcriptional. Post-transcriptional gene regulation can occur through alternative splicing, modified capping and polyadenylation, restriction of nuclear export, and translational inhibition [54]. Preliminary evidence from our laboratory shows that PRR15 interacts with proteins involved in mRNA processing and transport, such as heterogeneous nuclear ribonucleoprotein (hnRNP) A2/B1, hnRNP D0, lin28 homolog B, and nucleophosmin (JD Cantlon & RV Anthony, unpublished results). These interactions suggest that PRR15 could directly affect mRNA concentrations by modulating processing or splicing of initial transcripts or by sequestering mRNAs in nuclear bodies.

Our analysis was primarily conducted in ACH-3P cells, a fusion of primary first-trimester trophoblast cells with a choriocarcinoma cell line [14]. The fact that these cells are transformed for continuous culture and express some degree of tumorigenic potential could affect the transcriptome [55]. This was clearly borne out when relative expression of mRNA [52] and miRNA [53] were found to differ between primary trophoblast cells and a number of trophoblast-derived cell lines. The reality is that no cell line fully recapitulates the phenotype of primary trophoblast cells, and the phenotype of the latter changes with gestational age. Confirmation of the differentially expressed genes in primary trophoblast cells would reinforce the validity of our results. However, primary first-trimester human trophoblast cells are difficult to obtain and problematic to culture due to their rapid differentiation [56,57]. Limited time is allowed for altering gene expression prior to replicative senescence, making it unlikely that we could recapitulate these studies with primary human cytotrophoblasts. Regardless, our study sheds light on potential pathways involved in early placental development that may be critical to early embryonic survival. Furthermore, the demonstration of early embryonic loss when *PRR15* mRNA was targeted for degradation *in vivo* [10] supports a critical role for PRR15 and the pathways in which it functions for appropriate formation of the placenta during early pregnancy.

## Supporting information

S1 Table Differentially expressed genes from *PRR15* microarray. (DOC)

S2 Table Primers used for qPCR analysis. (DOC)
