# Supplementary material for: Effect of proline rich 15-deficiency on trophoblast viability and survival
Source: PLoS One. 2017 Apr 5;12(4):e0174976. doi: 10.1371/journal.pone.0174976 (PMC5381842; doi:10.1371/journal.pone.0174976)
Supplement: S1 Table — (DOC) [file pone.0174976.s001.doc]

S1 Table. Differentially expressed genes from *PRR15* microarray.

| **Genbank Acc** | **GENENAME** | **REFSEQ** | **P** | **Fold Change grp1/grp2** | **Fold Change** |
| --- | --- | --- | --- | --- | --- |
| L27624 | TFPI2 | NM_006528 | 0.0024 | 0.026 | -38.911 |
| BE883300 | PGBD1 | NM_032507 | 0.0035 | 0.207 | -4.822 |
| NM_002064 | GLRX | NM_002064 | 0.0004 | 0.225 | -4.452 |
| AF333388 | MT1P2 | NM_001039954 | 0.0104 | 0.251 | -3.990 |
| NM_002450 | MT1X | NM_005952 | 0.0041 | 0.252 | -3.973 |
| BF664545 |  |  | 0.0035 | 0.252 | -3.962 |
| T75480 | KCTD6 | NM_153331 | 0.0041 | 0.258 | -3.871 |
| AL162069 | KRT80 | NM_001081492 | 0.0050 | 0.269 | -3.723 |
| BE967019 | SPRED1 | NM_152594 | 0.0211 | 0.271 | -3.690 |
| AF039698 | TSHZ1 | NM_005786 | 0.0421 | 0.271 | -3.690 |
| BE466195 | RBM25 | NM_021239 | 0.0386 | 0.278 | -3.593 |
| AW051379 | LOC790955 | NM_001085372 | 0.0018 | 0.280 | -3.575 |
| NM_013238 | DNAJC15 | NM_013238 | 0.0417 | 0.282 | -3.547 |
| NM_004078 | CSRP1 | NM_004078 | 0.0048 | 0.287 | -3.479 |
| NM_005952 | MT1X | NM_005952 | 0.0259 | 0.291 | -3.438 |
| NM_017571 | CCDC88A | NM_018084 | 0.0429 | 0.300 | -3.331 |
| NM_021963 | NAP1L2 | NM_021963 | 0.0008 | 0.302 | -3.308 |
| N95414 | ITGA2 | NM_002203 | 0.0042 | 0.304 | -3.285 |
| BE222344 |  |  | 0.0008 | 0.318 | -3.147 |
| AW885748 |  |  | 0.0442 | 0.319 | -3.140 |
| C06331 | LOC399818 | NM_212554 | 0.0243 | 0.320 | -3.121 |
| NM_014125 | POLQ | NM_199420 | 0.0173 | 0.320 | -3.121 |
| NM_001394 | DUSP4 | NM_001394 | 0.0001 | 0.324 | -3.085 |
| AI827906 | LOC169834 | NM_001101338 | 0.0165 | 0.334 | -2.998 |
| AI768894 | CGN | NM_020770 | 0.0023 | 0.335 | -2.982 |
| AW069729 | ACPL2 | NM_001037172 | 0.0004 | 0.337 | -2.966 |
| AW963217 | NUDT19 | NM_001105570 | 0.0368 | 0.339 | -2.952 |
| NM_003877 | SOCS2 | NM_003877 | 0.0077 | 0.341 | -2.937 |
| BF593263 | NKAIN4 | NM_152864 | 0.0188 | 0.352 | -2.838 |
| NM_002426 | MMP12 | NM_002426 | 0.0050 | 0.354 | -2.822 |
| AA205660 | TRIM52 | NM_032765 | 0.0055 | 0.354 | -2.822 |
| NM_004328 | BCS1L | NM_001079866 | 0.0145 | 0.356 | -2.811 |
| AW170571 | CPNE2 | NM_152727 | 0.0246 | 0.357 | -2.800 |
| AI888594 | TTL | NM_153712 | 0.0248 | 0.359 | -2.789 |
| AI742551 | XAGE3 | NM_130776 | 0.0302 | 0.362 | -2.762 |
| AI920953 |  |  | 0.0014 | 0.363 | -2.756 |
| NM_001964 | EGR1 | NM_001964 | 0.0287 | 0.366 | -2.731 |
| AK001836 | KLHL5 | NM_001007075 | 0.0348 | 0.367 | -2.724 |
| BF593263 | NKAIN4 | NM_152864 | 0.0349 | 0.368 | -2.719 |
| AL832995 |  |  | 0.0171 | 0.369 | -2.711 |
| AW274756 | CDK6 | NM_001259 | 0.0118 | 0.375 | -2.668 |
| U79277 |  |  | 0.0002 | 0.376 | -2.660 |
| NM_013337 | TIMM22 | NM_013337 | 0.0106 | 0.379 | -2.636 |
| AL117589 | KIF26A | NM_015656 | 0.0021 | 0.381 | -2.623 |
| BE542563 | LOC728342 | XM_001129097 | 0.0068 | 0.384 | -2.608 |
| AI983896 |  |  | 0.0356 | 0.384 | -2.604 |
| AA776892 | LOC399818 | NM_212554 | 0.0128 | 0.385 | -2.599 |
| N74530 |  |  | 0.0193 | 0.385 | -2.599 |
| AI821399 |  |  | 0.0270 | 0.385 | -2.597 |
| AI669235 | ELAC1 | NM_018696 | 0.0373 | 0.385 | -2.596 |
| NM_000499 | CYP1A1 | NM_000499 | 0.0179 | 0.386 | -2.593 |
| AK024255 |  |  | 0.0004 | 0.386 | -2.591 |
| AU155515 | RPL37A | NM_000998 | 0.0314 | 0.388 | -2.577 |
| AW972359 |  |  | 0.0314 | 0.391 | -2.560 |
| AF276659 | MAP1LC3C | NM_001004343 | 0.0404 | 0.391 | -2.556 |
| NM_021083 | XK | NM_021083 | 0.0030 | 0.392 | -2.554 |
| AK023354 | UBQLN4 | NM_020131 | 0.0369 | 0.392 | -2.552 |
| AA835417 |  |  | 0.0024 | 0.392 | -2.552 |
| NM_018584 | CAMK2N1 | NM_018584 | 0.0081 | 0.393 | -2.545 |
| AI655015 |  |  | 0.0007 | 0.394 | -2.538 |
| BE964048 | TTL | NM_153712 | 0.0044 | 0.394 | -2.535 |
| NM_003246 | THBS1 | NM_003246 | 0.0024 | 0.397 | -2.521 |
| BI791845 |  |  | 0.0441 | 0.398 | -2.512 |
| AI021902 |  |  | 0.0008 | 0.400 | -2.499 |
| AJ278150 | AGK | NM_018238 | 0.0024 | 0.401 | -2.496 |
| NM_017542 | POGK | NM_017542 | 0.0224 | 0.401 | -2.491 |
| AW193600 | LOC439949 | XM_001128367 | 0.0007 | 0.404 | -2.478 |
| NM_030781 | COLEC12 | NM_030781 | 0.0150 | 0.405 | -2.470 |
| NM_024669 | ANKRD55 | NM_001039935 | 0.0016 | 0.405 | -2.470 |
| W60810 | TSHZ1 | NM_005786 | 0.0015 | 0.406 | -2.466 |
| AI654093 | LOC645431 | XR_015289 | 0.0175 | 0.406 | -2.462 |
| BC042908 | RRP12 | NM_015179 | 0.0423 | 0.407 | -2.455 |
| AW614120 | TMEM136 | NM_174926 | 0.0050 | 0.408 | -2.451 |
| AF010314 | ENC1 | NM_003633 | 0.0062 | 0.409 | -2.448 |
| AA191336 | ZNF496 | NM_032752 | 0.0190 | 0.410 | -2.437 |
| NM_003979 | GPRC5A | NM_003979 | 0.0341 | 0.411 | -2.435 |
| NM_013245 | VPS4A | NM_013245 | 0.0294 | 0.411 | -2.434 |
| AI928241 | FERMT2 | NM_006832 | 0.0387 | 0.412 | -2.430 |
| AW514267 | LOC202134 /// LOC653316 /// NY-REN-7 | NM_001079527 | 0.0414 | 0.413 | -2.424 |
| AW975638 | HK2 | NM_000189 | 0.0213 | 0.413 | -2.422 |
| BG493862 | TCHP | NM_032300 | 0.0282 | 0.413 | -2.421 |
| NM_005103 | FEZ1 | NM_005103 | 0.0383 | 0.415 | -2.409 |
| NM_000296 | PKD1 | NM_000296 | 0.0075 | 0.416 | -2.403 |
| NM_007240 | DUSP12 | NM_007240 | 0.0150 | 0.417 | -2.398 |
| AA045184 | S100A16 | NM_080388 | 0.0101 | 0.419 | -2.388 |
| NM_001393 | ECM2 | NM_001393 | 0.0082 | 0.419 | -2.386 |
| AV693653 | TNRC6B | NM_001024843 | 0.0381 | 0.420 | -2.383 |
| NM_004395 | DBN1 | NM_004395 | 0.0445 | 0.420 | -2.381 |
| BC006148 | OVOL2 | NM_021220 | 0.0298 | 0.420 | -2.379 |
| NM_005953 | MT2A | NM_005953 | 0.0123 | 0.421 | -2.375 |
| AI870369 | ZNF553 | NM_152652 | 0.0339 | 0.422 | -2.368 |
| BE795648 | SSRP1 | NM_003146 | 0.0180 | 0.423 | -2.365 |
| T68150 | PHLDB2 | NM_145753 | 0.0026 | 0.423 | -2.364 |
| AA468591 | CLK4 | NM_020666 | 0.0005 | 0.425 | -2.355 |
| NM_014724 | ZSCAN12 | NM_001039643 | 0.0304 | 0.425 | -2.353 |
| AI479440 |  |  | 0.0258 | 0.426 | -2.349 |
| AL110225 | DBN1 | NM_004395 | 0.0485 | 0.426 | -2.348 |
| AI884858 | TUSC3 | NM_006765 | 0.0221 | 0.427 | -2.341 |
| NM_002766 | PRPSAP1 | NM_002766 | 0.0019 | 0.430 | -2.328 |
| AW971198 | GRAMD3 | NM_023927 | 0.0500 | 0.430 | -2.326 |
| AW294686 | TTBK2 | NM_173500 | 0.0499 | 0.431 | -2.318 |
| AI613273 | CHD4 | NM_001273 | 0.0001 | 0.435 | -2.300 |
| NM_003146 | SSRP1 | NM_003146 | 0.0122 | 0.436 | -2.293 |
| NM_005416 | SPRR3 | NM_001097589 | 0.0113 | 0.437 | -2.290 |
| D60621 | LPHN3 | NM_015236 | 0.0237 | 0.438 | -2.285 |
| AI948503 | ABCC4 | NM_001105515 | 0.0035 | 0.438 | -2.285 |
| NM_003157 | NEK4 | NM_003157 | 0.0057 | 0.438 | -2.283 |
| NM_014962 | BTBD3 | NM_014962 | 0.0062 | 0.439 | -2.277 |
| AW206286 |  |  | 0.0282 | 0.440 | -2.275 |
| NM_014583 | LMCD1 | NM_014583 | 0.0095 | 0.440 | -2.275 |
| AA701657 | LIFR | NM_002310 | 0.0251 | 0.441 | -2.266 |
| NM_016034 | MRPS2 | NM_016034 | 0.0472 | 0.443 | -2.257 |
| M34421 | PSG9 | NM_002784 | 0.0189 | 0.445 | -2.249 |
| AI692432 | ARID2 | NM_152641 | 0.0325 | 0.445 | -2.247 |
| AI741586 | ZNF720 | NM_001004300 | 0.0116 | 0.446 | -2.244 |
| NM_014344 | FJX1 | NM_014344 | 0.0109 | 0.449 | -2.230 |
| BC014479 | PXK | NM_017771 | 0.0212 | 0.449 | -2.227 |
| AI261467 | IKZF4 | NM_022465 | 0.0309 | 0.450 | -2.222 |
| Z24725 | FERMT2 | NM_006832 | 0.0006 | 0.451 | -2.219 |
| AK022566 | B4GALT7 | NM_007255 | 0.0355 | 0.451 | -2.218 |
| NM_006596 | POLQ | NM_199420 | 0.0126 | 0.451 | -2.217 |
| AK001697 | RIOK2 | NM_018343 | 0.0009 | 0.451 | -2.217 |
| BG291550 | FYTTD1 | NM_001011537 | 0.0011 | 0.452 | -2.214 |
| NM_014950 | ZBTB1 | NM_014950 | 0.0364 | 0.452 | -2.214 |
| AW592684 | LIFR | NM_002310 | 0.0078 | 0.453 | -2.208 |
| AJ003062 | TUBGCP3 | NM_006322 | 0.0136 | 0.453 | -2.206 |
| AL530462 | ZNF364 | NM_014455 | 0.0237 | 0.454 | -2.203 |
| NM_007361 | NID2 | NM_007361 | 0.0250 | 0.454 | -2.201 |
| AY114106 | GEMIN7 | NM_001007269 | 0.0474 | 0.455 | -2.196 |
| H05812 | IGF1R | NM_000875 | 0.0052 | 0.456 | -2.194 |
| NM_004124 | GMFB | NM_004124 | 0.0047 | 0.457 | -2.186 |
| W22690 | C1orf175 /// TTC4 | NM_001039464 | 0.0008 | 0.458 | -2.182 |
| NM_022443 | MLF1 | NM_022443 | 0.0057 | 0.460 | -2.176 |
| BC002791 | FLJ35348 | NR_002800 | 0.0029 | 0.460 | -2.174 |
| AW971205 |  |  | 0.0004 | 0.460 | -2.172 |
| NM_024597 | MAP7D3 | NM_024597 | 0.0493 | 0.460 | -2.172 |
| H17038 | FLJ25076 | XM_059689 | 0.0011 | 0.461 | -2.172 |
| AI701430 | MLL | NM_005933 | 0.0056 | 0.462 | -2.166 |
| NM_007038 | ADAMTS5 | NM_007038 | 0.0203 | 0.462 | -2.165 |
| BF060767 | ADAMTS5 | NM_007038 | 0.0099 | 0.462 | -2.164 |
| NM_014391 | ANKRD1 | NM_014391 | 0.0139 | 0.462 | -2.163 |
| AA527587 | ZNF498 | NM_145115 | 0.0094 | 0.463 | -2.158 |
| NM_014830 | ZBTB39 | NM_014830 | 0.0046 | 0.464 | -2.157 |
| NM_006322 | TUBGCP3 | NM_006322 | 0.0013 | 0.464 | -2.155 |
| NM_018478 | DBNDD2 /// SYS1-DBNDD2 | NM_001048221 | 0.0001 | 0.465 | -2.151 |
| AL050297 | R3HCC1 | XM_114618 | 0.0172 | 0.465 | -2.151 |
| AU145127 | FBXL7 | NM_012304 | 0.0329 | 0.467 | -2.143 |
| BQ944989 | STRAP | NM_007178 | 0.0123 | 0.467 | -2.143 |
| AV726956 | BEX5 | NM_001012978 | 0.0335 | 0.467 | -2.139 |
| N62996 | ZNF70 | NM_021916 | 0.0377 | 0.468 | -2.137 |
| AF087573 | DFFA | NM_004401 | 0.0080 | 0.469 | -2.132 |
| AA789332 | VANGL1 | NM_138959 | 0.0015 | 0.469 | -2.130 |
| AI983428 | COL5A1 | NM_000093 | 0.0372 | 0.470 | -2.130 |
| NM_001036 | RYR3 | NM_001036 | 0.0116 | 0.470 | -2.129 |
| AI937060 | NAV1 | NM_020443 | 0.0440 | 0.470 | -2.129 |
| BE856822 | C3orf39 | NM_032806 | 0.0265 | 0.470 | -2.127 |
| NM_000216 | KAL1 | NM_000216 | 0.0197 | 0.471 | -2.124 |
| AF247167 | TMEM133 | NM_032021 | 0.0058 | 0.471 | -2.122 |
| BG251218 | RBM25 | NM_021239 | 0.0445 | 0.472 | -2.120 |
| AL136932 | KIAA0922 | NM_015196 | 0.0016 | 0.473 | -2.116 |
| AL080170 | TRIM58 | NM_015431 | 0.0307 | 0.473 | -2.115 |
| AW291487 | NHS | NM_198270 | 0.0175 | 0.473 | -2.115 |
| NM_024534 | LOC728193 | XM_001128013 | 0.0032 | 0.475 | -2.104 |
| M31159 | IGFBP3 | NM_000598 | 0.0080 | 0.476 | -2.101 |
| W72455 | ZNF362 | NM_152493 | 0.0068 | 0.476 | -2.101 |
| AA715041 | MLL | NM_005933 | 0.0430 | 0.476 | -2.099 |
| BF684446 | AXIN2 | NM_004655 | 0.0237 | 0.477 | -2.099 |
| AA100793 | LMO7 | NM_005358 | 0.0274 | 0.477 | -2.098 |
| BC006118 | ZKSCAN3 | NM_024493 | 0.0479 | 0.477 | -2.097 |
| AF317887 | CEP290 | NM_025114 | 0.0098 | 0.477 | -2.096 |
| AI695695 |  |  | 0.0062 | 0.478 | -2.094 |
| NM_018238 | AGK | NM_018238 | 0.0260 | 0.479 | -2.087 |
| AK021539 | DSEL | NM_032160 | 0.0027 | 0.480 | -2.086 |
| NM_004494 | HDGF | NM_004494 | 0.0001 | 0.480 | -2.083 |
| NM_004623 | TTC4 | NM_004623 | 0.0358 | 0.481 | -2.081 |
| AI684747 | PXK | NM_017771 | 0.0493 | 0.482 | -2.075 |
| AF033861 | ADCY3 | NM_004036 | 0.0106 | 0.484 | -2.066 |
| AW044606 | TTC5 | NM_138376 | 0.0028 | 0.485 | -2.062 |
| BC030710 | TMEM74 | NM_153015 | 0.0076 | 0.485 | -2.061 |
| X79780 | RAB11B | NM_004218 | 0.0243 | 0.485 | -2.060 |
| BC015881 | STRA6 | NM_022369 | 0.0239 | 0.486 | -2.058 |
| AK024318 | USP46 | NM_022832 | 0.0079 | 0.486 | -2.058 |
| AK022622 | NAV1 | NM_020443 | 0.0005 | 0.487 | -2.055 |
| AA020010 | KLF12 | NM_007249 | 0.0290 | 0.487 | -2.054 |
| NM_005756 | GPR64 | NM_001079858 | 0.0225 | 0.487 | -2.052 |
| AI307763 | VTI1B | NM_006370 | 0.0170 | 0.489 | -2.047 |
| M29277 | MCAM | NM_006500 | 0.0079 | 0.491 | -2.036 |
| NM_012342 | BAMBI | NM_012342 | 0.0045 | 0.491 | -2.036 |
| AY078987 | GTPBP3 | NM_032620 | 0.0195 | 0.492 | -2.034 |
| BF002121 |  |  | 0.0252 | 0.492 | -2.031 |
| AI692880 | GJA5 | NM_005266 | 0.0146 | 0.492 | -2.031 |
| AA361361 | MAP3K1 | NM_005921 | 0.0329 | 0.492 | -2.031 |
| AI521273 |  |  | 0.0382 | 0.493 | -2.030 |
| AI824012 | NRIP1 | NM_003489 | 0.0041 | 0.493 | -2.030 |
| AK021888 |  |  | 0.0455 | 0.493 | -2.028 |
| BF513233 | LOC284952 | XM_001126137 | 0.0324 | 0.493 | -2.027 |
| AA214704 | TNRC6B | NM_001024843 | 0.0094 | 0.494 | -2.026 |
| AI911518 | GPATCH4 | NM_015590 | 0.0241 | 0.494 | -2.025 |
| AW117765 | PEX13 | NM_002618 | 0.0217 | 0.495 | -2.022 |
| BC002671 | DUSP4 | NM_001394 | 0.0081 | 0.495 | -2.021 |
| BC013912 | TTC26 | NM_024926 | 0.0288 | 0.495 | -2.021 |
| NM_018079 | SRBD1 | NM_018079 | 0.0109 | 0.495 | -2.021 |
| AI200443 | MAGEA5 | NM_021049 | 0.0263 | 0.495 | -2.020 |
| BC002827 | TPM4 | NM_003290 | 0.0277 | 0.495 | -2.020 |
| NM_012460 | TIMM9 | NM_012460 | 0.0049 | 0.496 | -2.015 |
| BC038589 |  |  | 0.0123 | 0.497 | -2.014 |
| AK001007 |  |  | 0.0353 | 0.497 | -2.011 |
| AW002876 |  |  | 0.0077 | 0.497 | -2.010 |
| N31717 | RIPK5 | NM_015375 | 0.0208 | 0.498 | -2.008 |
| AW469573 | FERMT2 | NM_006832 | 0.0142 | 0.498 | -2.008 |
| AI183453 | AARS2 | NM_020745 | 0.0138 | 0.499 | -2.005 |
| W31002 | ZNF498 | NM_145115 | 0.0198 | 0.499 | -2.004 |
| BC038557 |  |  | 0.0354 | 0.500 | -1.998 |
| NM_014817 | KIAA0644 | NM_014817 | 0.0398 | 0.501 | -1.998 |
| AI130969 | COL5A1 | NM_000093 | 0.0128 | 0.501 | -1.994 |
| NM_018343 | RIOK2 | NM_018343 | 0.0070 | 0.502 | -1.993 |
| NM_022483 | C5orf28 | NM_022483 | 0.0277 | 0.503 | -1.989 |
| AA524029 | C9orf61 | NM_004816 | 0.0280 | 0.503 | -1.987 |
| BF513384 |  |  | 0.0096 | 0.504 | -1.985 |
| NM_006466 | POLR3F | NM_006466 | 0.0144 | 0.504 | -1.984 |
| AA788946 | COL12A1 | NM_004370 | 0.0115 | 0.506 | -1.976 |
| NM_007018 | CEP110 | NM_007018 | 0.0026 | 0.506 | -1.976 |
| NM_021964 | ZNF148 | NM_021964 | 0.0022 | 0.508 | -1.970 |
| AV724192 | KIAA0644 | NM_014817 | 0.0066 | 0.508 | -1.969 |
| AI679268 | PIK3R1 | NM_181504 | 0.0280 | 0.508 | -1.967 |
| AA976778 | WDR32 | NM_024345 | 0.0045 | 0.509 | -1.966 |
| AI739389 | SF3B1 | NM_001005526 | 0.0312 | 0.510 | -1.960 |
| AL831862 | TNRC6B | NM_001024843 | 0.0035 | 0.512 | -1.955 |
| AV703555 |  |  | 0.0101 | 0.513 | -1.951 |
| AI917328 | WDR75 | NM_032168 | 0.0005 | 0.513 | -1.950 |
| N57538 | NAV1 | NM_020443 | 0.0155 | 0.513 | -1.949 |
| NM_004759 | MAPKAPK2 | NM_004759 | 0.0437 | 0.514 | -1.947 |
| AF124145 | AMFR | NM_001144 | 0.0054 | 0.514 | -1.947 |
| AI277642 | CDCA7 | NM_031942 | 0.0473 | 0.514 | -1.945 |
| AI471723 | RBM45 | NM_152945 | 0.0070 | 0.514 | -1.945 |
| NM_001784 | CD97 | NM_001025160 | 0.0158 | 0.514 | -1.944 |
| BF109381 |  |  | 0.0493 | 0.514 | -1.944 |
| BF446943 |  |  | 0.0102 | 0.515 | -1.941 |
| BC029425 | FILIP1 | NM_015687 | 0.0361 | 0.516 | -1.939 |
| AB033007 | ERGIC1 | NM_001031711 | 0.0188 | 0.516 | -1.938 |
| AW103422 | PCBP2 | NM_001098620 | 0.0415 | 0.516 | -1.937 |
| BC034621 | LPGAT1 | NM_014873 | 0.0240 | 0.517 | -1.936 |
| NM_014159 | SETD2 | NM_014159 | 0.0220 | 0.517 | -1.935 |
| AI174988 |  |  | 0.0189 | 0.518 | -1.930 |
| AF059317 | RSF1 | NM_016578 | 0.0487 | 0.519 | -1.926 |
| NM_018495 | CALD1 | NM_004342 | 0.0301 | 0.519 | -1.926 |
| NM_007066 | PKIG | NM_007066 | 0.0192 | 0.519 | -1.925 |
| AU153412 | OPRK1 | NM_000912 | 0.0083 | 0.519 | -1.925 |
| NM_020354 | ENTPD7 | NM_020354 | 0.0294 | 0.520 | -1.925 |
| AI299467 |  |  | 0.0204 | 0.520 | -1.924 |
| AA580691 | RBM25 | NM_021239 | 0.0130 | 0.520 | -1.923 |
| AU118165 | ZNF37A /// ZNF37B | NM_001007094 | 0.0126 | 0.520 | -1.923 |
| NM_000248 | MITF | NM_000248 | 0.0101 | 0.520 | -1.922 |
| AI094626 | OSBPL6 | NM_032523 | 0.0385 | 0.521 | -1.920 |
| AB033105 | KIAA1279 | NM_015634 | 0.0030 | 0.523 | -1.914 |
| BE379761 | STON2 | NM_033104 | 0.0077 | 0.523 | -1.913 |
| NM_002402 | MEST | NM_002402 | 0.0468 | 0.523 | -1.913 |
| AA765470 |  |  | 0.0114 | 0.524 | -1.909 |
| NM_016010 | C8orf70 | NM_016010 | 0.0067 | 0.524 | -1.909 |
| BF059479 | FLJ14712 | XM_001131663 | 0.0292 | 0.524 | -1.909 |
| AL512725 | MIDN | NM_177401 | 0.0439 | 0.524 | -1.907 |
| AB037776 | IGSF9 | NM_020789 | 0.0016 | 0.524 | -1.907 |
| AI623155 | TRAF3IP1 | NM_015650 | 0.0144 | 0.524 | -1.907 |
| AI377688 | GTF2H1 | NM_005316 | 0.0332 | 0.525 | -1.907 |
| NM_022344 | C17orf75 | NM_022344 | 0.0462 | 0.525 | -1.904 |
| NM_001150 | ANPEP | NM_001150 | 0.0286 | 0.526 | -1.900 |
| AV699825 | LOC145786 |  | 0.0024 | 0.528 | -1.895 |
| AI525402 | LPHN1 | NM_001008701 | 0.0405 | 0.528 | -1.894 |
| NM_002431 | MNAT1 | NM_002431 | 0.0134 | 0.528 | -1.894 |
| AA504356 | PCBP2 | NM_001098620 | 0.0013 | 0.528 | -1.894 |
| BC005359 | GMFB | NM_004124 | 0.0008 | 0.529 | -1.892 |
| AK023585 | NSFL1C | NM_016143 | 0.0193 | 0.529 | -1.890 |
| NM_023008 | KRI1 | NM_023008 | 0.0420 | 0.529 | -1.890 |
| AB002364 | ADAMTS3 | NM_014243 | 0.0017 | 0.529 | -1.889 |
| NM_006227 | PLTP | NM_006227 | 0.0422 | 0.530 | -1.887 |
| BE544096 | UBE2Z | NM_023079 | 0.0096 | 0.530 | -1.885 |
| NM_030952 | C11orf17 /// NUAK2 | NM_020642 | 0.0145 | 0.532 | -1.881 |
| NM_015909 | NAG | NM_015909 | 0.0019 | 0.532 | -1.881 |
| NM_016205 | PDGFC | NM_016205 | 0.0034 | 0.532 | -1.881 |
| AK001574 | GORASP1 | NM_031899 | 0.0016 | 0.532 | -1.879 |
| NM_005414 | SKIL | NM_005414 | 0.0116 | 0.532 | -1.879 |
| AW305097 | OLFML1 | NM_198474 | 0.0007 | 0.533 | -1.878 |
| NM_006738 | AKAP13 | NM_006738 | 0.0365 | 0.533 | -1.877 |
| BC000254 | ACVR1B | NM_004302 | 0.0123 | 0.534 | -1.874 |
| BE676248 | DEF8 | NM_017702 | 0.0478 | 0.534 | -1.872 |
| BE907791 | GTPBP8 | NM_001008235 | 0.0156 | 0.534 | -1.872 |
| AW194766 | CDK6 | NM_001259 | 0.0154 | 0.535 | -1.871 |
| AW409794 | FAM80B | NM_020734 | 0.0003 | 0.535 | -1.869 |
| NM_021813 | BACH2 | NM_021813 | 0.0316 | 0.535 | -1.869 |
| W73820 | KCTD15 | NM_024076 | 0.0163 | 0.537 | -1.863 |
| NM_144990 | SLFNL1 | NM_144990 | 0.0356 | 0.537 | -1.862 |
| AA740754 | BCLAF1 | NM_001077440 | 0.0077 | 0.538 | -1.858 |
| AW025928 |  |  | 0.0206 | 0.538 | -1.858 |
| BE271180 |  |  | 0.0187 | 0.539 | -1.855 |
| U79297 | ANKRD46 | NM_198401 | 0.0013 | 0.539 | -1.855 |
| AL578102 | IL20RB | NM_144717 | 0.0147 | 0.540 | -1.851 |
| AW051349 | CDK6 | NM_001259 | 0.0160 | 0.540 | -1.850 |
| AK024273 | COPS7B | NM_022730 | 0.0212 | 0.541 | -1.850 |
| BF197274 |  |  | 0.0029 | 0.541 | -1.850 |
| AF070571 | EXT1 |  | 0.0428 | 0.542 | -1.844 |
| AK095622 | C1orf61 | NM_006365 | 0.0280 | 0.544 | -1.838 |
| AA259174 | TMED5 | NM_016040 | 0.0029 | 0.544 | -1.837 |
| AK094821 | ATAD2B | NM_017552 | 0.0368 | 0.545 | -1.836 |
| AU144734 | NASP | NM_002482 | 0.0247 | 0.545 | -1.834 |
| AA872583 | SERINC2 | NM_178865 | 0.0454 | 0.545 | -1.834 |
| AB028957 | SATB2 | NM_015265 | 0.0310 | 0.546 | -1.833 |
| AL138455 | SHROOM1 | NM_133456 | 0.0033 | 0.547 | -1.830 |
| AI040029 | B4GALT7 | NM_007255 | 0.0041 | 0.547 | -1.829 |
| NM_017745 | BCOR | NM_017745 | 0.0260 | 0.547 | -1.828 |
| NM_024724 | ZBTB38 | NM_001080412 | 0.0007 | 0.547 | -1.827 |
| AI766311 | LOC162073 | NM_001034841 | 0.0455 | 0.548 | -1.823 |
| AK026220 | MRPL35 | NM_016622 | 0.0031 | 0.549 | -1.822 |
| AW297204 | NHLRC2 | NM_198514 | 0.0202 | 0.549 | -1.821 |
| BC001247 | LIMA1 | NM_016357 | 0.0040 | 0.550 | -1.819 |
| AW265065 |  |  | 0.0010 | 0.550 | -1.818 |
| NM_024513 | FYCO1 | NM_024513 | 0.0117 | 0.551 | -1.815 |
| NM_003633 | ENC1 | NM_003633 | 0.0034 | 0.552 | -1.811 |
| NM_024010 | MTRR | NM_002454 | 0.0031 | 0.552 | -1.810 |
| U20165 | BMPR2 | NM_001204 | 0.0181 | 0.553 | -1.810 |
| AI219740 | LSG1 | NM_018385 | 0.0465 | 0.553 | -1.809 |
| N32508 | GNG12 | NM_018841 | 0.0163 | 0.554 | -1.807 |
| NM_005211 | CSF1R | NM_005211 | 0.0129 | 0.554 | -1.807 |
| NM_017651 | AHI1 | NM_017651 | 0.0171 | 0.556 | -1.799 |
| AI814644 | WDR22 | NM_003861 | 0.0446 | 0.556 | -1.798 |
| AI917716 | LOXL3 | NM_032603 | 0.0021 | 0.556 | -1.798 |
| NM_018650 | MARK1 | NM_018650 | 0.0100 | 0.557 | -1.796 |
| NM_007173 | PRSS23 | NM_007173 | 0.0329 | 0.557 | -1.794 |
| AW104509 | ARID2 | NM_152641 | 0.0124 | 0.557 | -1.794 |
| AF117234 | FLOT1 | NM_005803 | 0.0082 | 0.559 | -1.791 |
| AI953362 | EIF2AK4 | NM_001013703 | 0.0304 | 0.560 | -1.786 |
| BF749719 |  |  | 0.0224 | 0.560 | -1.786 |
| AA905942 | TEAD2 | NM_003598 | 0.0299 | 0.560 | -1.785 |
| N30339 | COL5A1 | NM_000093 | 0.0107 | 0.561 | -1.784 |
| AB032983 | PPM1H | NM_020700 | 0.0353 | 0.561 | -1.783 |
| AA207013 | CLUAP1 | NM_015041 | 0.0212 | 0.561 | -1.782 |
| NM_018178 | GOLPH3L | NM_018178 | 0.0043 | 0.562 | -1.780 |
| BF696912 | EXOC5 | NM_006544 | 0.0140 | 0.562 | -1.778 |
| NM_014840 | NUAK1 | NM_014840 | 0.0068 | 0.564 | -1.773 |
| NM_015322 | FEM1B | NM_015322 | 0.0308 | 0.565 | -1.771 |
| AI743612 | FAM80B | NM_020734 | 0.0161 | 0.565 | -1.771 |
| BE083088 | SSFA2 | NM_006751 | 0.0455 | 0.565 | -1.771 |
| NM_004672 | MAP3K6 | NM_004672 | 0.0191 | 0.565 | -1.770 |
| AI796536 |  |  | 0.0492 | 0.565 | -1.770 |
| AL578583 | APITD1 | NM_198544 | 0.0305 | 0.565 | -1.769 |
| AF057354 | MTMR1 | NM_003828 | 0.0009 | 0.566 | -1.767 |
| BE963370 | BCLAF1 | NM_001077440 | 0.0176 | 0.566 | -1.767 |
| NM_017687 | NHLRC2 | NM_198514 | 0.0291 | 0.567 | -1.765 |
| AI167164 | MTMR1 | NM_003828 | 0.0053 | 0.567 | -1.763 |
| BM987612 |  |  | 0.0448 | 0.568 | -1.761 |
| U32645 | ELF4 | NM_001421 | 0.0013 | 0.569 | -1.759 |
| AW467480 |  |  | 0.0204 | 0.570 | -1.756 |
| BF526978 |  |  | 0.0291 | 0.570 | -1.755 |
| C18965 |  |  | 0.0404 | 0.570 | -1.755 |
| AW003030 | SF3B1 | NM_001005526 | 0.0137 | 0.570 | -1.754 |
| AF245505 | MXRA5 | NM_015419 | 0.0189 | 0.570 | -1.754 |
| X99268 | TWIST1 | NM_000474 | 0.0252 | 0.570 | -1.754 |
| AA704766 | MLL | NM_005933 | 0.0456 | 0.571 | -1.751 |
| BG170743 | EXOC5 | NM_006544 | 0.0000 | 0.571 | -1.751 |
| AI936517 | NEK1 | NM_012224 | 0.0240 | 0.571 | -1.750 |
| AW117498 | FOXO1 | NM_002015 | 0.0087 | 0.571 | -1.750 |
| AW165979 | ZNF609 | NM_015042 | 0.0047 | 0.571 | -1.750 |
| BF878343 | COX15 | NM_004376 | 0.0307 | 0.572 | -1.749 |
| AW304871 |  |  | 0.0289 | 0.572 | -1.749 |
| NM_014478 | RCP9 | NM_001040647 | 0.0364 | 0.572 | -1.748 |
| NM_000633 | BCL2 | NM_000633 | 0.0474 | 0.573 | -1.746 |
| AW139179 | FEM1B | NM_015322 | 0.0038 | 0.573 | -1.744 |
| NM_024926 | TTC26 | NM_024926 | 0.0161 | 0.574 | -1.743 |
| BE620457 | NRP1 | NM_001024628 | 0.0295 | 0.574 | -1.742 |
| BC019922 | ZNF252 |  | 0.0043 | 0.574 | -1.742 |
| BC032757 | LOC219731 |  | 0.0223 | 0.574 | -1.742 |
| L04282 | ZNF148 | NM_021964 | 0.0037 | 0.574 | -1.741 |
| AA536004 | RNF169 | NM_001098638 | 0.0076 | 0.575 | -1.740 |
| BF215996 | MYO1B | NM_012223 | 0.0007 | 0.575 | -1.739 |
| NM_015062 | PPRC1 | NM_015062 | 0.0203 | 0.575 | -1.739 |
| AI807026 | CBL | NM_005188 | 0.0438 | 0.575 | -1.739 |
| NM_017650 | PPP1R9A | NM_017650 | 0.0038 | 0.577 | -1.734 |
| NM_024773 | JMJD5 | NM_024773 | 0.0108 | 0.577 | -1.734 |
| BF940043 | NID1 | NM_002508 | 0.0196 | 0.577 | -1.733 |
| AA977481 |  |  | 0.0241 | 0.577 | -1.732 |
| AB020719 | CEP152 | NM_014985 | 0.0348 | 0.578 | -1.732 |
| AF231056 | ARID1A | NM_006015 | 0.0228 | 0.578 | -1.731 |
| AA551075 | KCTD12 | NM_138444 | 0.0040 | 0.578 | -1.731 |
| AK002174 | KLHL5 | NM_001007075 | 0.0018 | 0.578 | -1.730 |
| AA912476 | LOC145786 |  | 0.0005 | 0.578 | -1.730 |
| NM_005558 | LAD1 | NM_005558 | 0.0436 | 0.578 | -1.729 |
| AF465843 | ZAK | NM_016653 | 0.0062 | 0.578 | -1.729 |
| AI041854 | SFRS15 | NM_020706 | 0.0004 | 0.580 | -1.725 |
| NM_003607 | CDC42BPA | NM_003607 | 0.0488 | 0.580 | -1.724 |
| AI282485 | BAT1 | NM_004640 | 0.0331 | 0.580 | -1.723 |
| AA777641 | KIAA0157 | NM_032182 | 0.0200 | 0.581 | -1.722 |
| NM_004401 | DFFA | NM_004401 | 0.0263 | 0.581 | -1.721 |
| N40199 | LOC729810 | XM_001131395 | 0.0140 | 0.581 | -1.720 |
| N92507 | HMGB1 | NM_002128 | 0.0272 | 0.581 | -1.720 |
| AL033538 | TTC28 | XM_929318 | 0.0107 | 0.582 | -1.719 |
| AF263462 | CGN | NM_020770 | 0.0058 | 0.582 | -1.719 |
| AA922068 | CDK6 | NM_001259 | 0.0035 | 0.582 | -1.718 |
| AW242125 | USP54 | NM_152586 | 0.0123 | 0.582 | -1.717 |
| AK022838 |  |  | 0.0317 | 0.583 | -1.716 |
| NM_016129 | COPS4 | NM_016129 | 0.0292 | 0.583 | -1.716 |
| NM_006942 | SOX15 | NM_006942 | 0.0151 | 0.583 | -1.715 |
| NM_007203 | AKAP2 /// PALM2-AKAP2 | NM_001004065 | 0.0303 | 0.583 | -1.715 |
| AB033831 | PDGFC | NM_016205 | 0.0168 | 0.583 | -1.715 |
| AU146891 | SMAD1 | NM_001003688 | 0.0243 | 0.583 | -1.714 |
| BG054922 | CCDC113 | NM_014157 | 0.0407 | 0.584 | -1.714 |
| BC000822 | C16orf58 | NM_022744 | 0.0074 | 0.584 | -1.714 |
| AF155117 | KIF21A | NM_017641 | 0.0164 | 0.584 | -1.713 |
| D42044 | KIAA0090 | NM_015047 | 0.0131 | 0.584 | -1.713 |
| NM_016561 | BFAR | NM_016561 | 0.0186 | 0.584 | -1.711 |
| D79987 | ESPL1 | NM_012291 | 0.0243 | 0.584 | -1.711 |
| AI040432 | TM9SF3 | NM_020123 | 0.0067 | 0.585 | -1.711 |
| AV700132 | SIAH1 | NM_001006610 | 0.0364 | 0.585 | -1.710 |
| NM_015694 | ZNF777 | NM_015694 | 0.0280 | 0.585 | -1.709 |
| NM_003370 | VASP | NM_001008736 | 0.0272 | 0.586 | -1.708 |
| AW055205 | ARL6IP2 | NM_022374 | 0.0483 | 0.586 | -1.707 |
| NM_002015 | FOXO1 | NM_002015 | 0.0073 | 0.586 | -1.707 |
| AI700188 | ZNF30 | NM_001099437 | 0.0214 | 0.586 | -1.706 |
| NM_000305 | PON2 | NM_000305 | 0.0015 | 0.586 | -1.705 |
| U87460 | GPR37 | NM_005302 | 0.0328 | 0.586 | -1.705 |
| BE878463 |  |  | 0.0252 | 0.587 | -1.705 |
| AW264082 | FAM110B | NM_147189 | 0.0250 | 0.587 | -1.704 |
| BG288755 |  |  | 0.0184 | 0.587 | -1.704 |
| NM_014783 | ARHGAP11A | NM_014783 | 0.0178 | 0.587 | -1.704 |
| AW264273 | ZNF445 | NM_181489 | 0.0398 | 0.587 | -1.703 |
| BC000761 | SNAPIN | NM_012437 | 0.0391 | 0.587 | -1.703 |
| AK026898 | FOXP1 | NM_001012505 | 0.0380 | 0.587 | -1.702 |
| AW612461 |  |  | 0.0163 | 0.588 | -1.702 |
| U20489 | PTPRO | NM_002848 | 0.0061 | 0.588 | -1.702 |
| AC004770 | C11orf9 | NM_013279 | 0.0327 | 0.588 | -1.701 |
| BC029890 | LOC653110 /// LOC728449 | XM_001128973 | 0.0008 | 0.588 | -1.701 |
| BC007934 | ARMC8 | NM_014154 | 0.0461 | 0.588 | -1.701 |
| BF062828 | BRWD2 | NM_018117 | 0.0477 | 0.588 | -1.701 |
| H43976 | MORF4L2 | NM_012286 | 0.0109 | 0.589 | -1.699 |
| AK026659 |  |  | 0.0072 | 0.589 | -1.697 |
| AI580162 | BTBD7 | NM_001002860 | 0.0161 | 0.590 | -1.696 |
| T16257 | GPR37 | NM_005302 | 0.0274 | 0.590 | -1.696 |
| M10943 | MT1F | NM_005949 | 0.0343 | 0.590 | -1.696 |
| BF432550 | MYO1B | NM_012223 | 0.0389 | 0.591 | -1.693 |
| N51597 | SFRS12 | NM_001077199 | 0.0119 | 0.591 | -1.693 |
| BC002836 | EFCAB2 | NM_032328 | 0.0007 | 0.592 | -1.690 |
| BC012090 | HNRPA3 | NM_194247 | 0.0322 | 0.592 | -1.689 |
| NM_018321 | BXDC2 | NM_018321 | 0.0416 | 0.592 | -1.689 |
| T16443 | SNHG5 /// SNORD50A /// SNORD50B | NR_002743 | 0.0444 | 0.593 | -1.686 |
| BE502826 |  |  | 0.0095 | 0.593 | -1.686 |
| NM_006599 | NFAT5 | NM_001113178 | 0.0350 | 0.593 | -1.685 |
| AW135003 |  |  | 0.0188 | 0.594 | -1.685 |
| BG285417 | SH3D19 | NM_001009555 | 0.0156 | 0.594 | -1.684 |
| NM_005983 | SKP2 | NM_005983 | 0.0178 | 0.594 | -1.684 |
| AA037483 | HIST1H2BC | NM_003526 | 0.0297 | 0.594 | -1.683 |
| AB023179 | DNAJC16 | NM_015291 | 0.0337 | 0.594 | -1.683 |
| AK002200 | SMC4 | NM_001002799 | 0.0112 | 0.595 | -1.681 |
| BF057682 | C14orf131 | NM_018335 | 0.0010 | 0.595 | -1.680 |
| L24521 | HDGF | NM_004494 | 0.0122 | 0.596 | -1.679 |
| H97931 | SPRED2 | NM_181784 | 0.0233 | 0.596 | -1.678 |
| NM_024520 | C2orf47 | NM_024520 | 0.0420 | 0.596 | -1.678 |
| BF594371 | TNRC6B | NM_001024843 | 0.0464 | 0.596 | -1.678 |
| NM_001273 | CHD4 | NM_001273 | 0.0272 | 0.596 | -1.677 |
| AW242920 |  |  | 0.0058 | 0.596 | -1.677 |
| NM_022838 | ARMCX5 | NM_022838 | 0.0152 | 0.597 | -1.676 |
| AF234161 | CIZ1 | NM_012127 | 0.0026 | 0.597 | -1.674 |
| NM_004523 | KIF11 | NM_004523 | 0.0124 | 0.598 | -1.673 |
| AI380704 | BOLA3 | NM_001035505 | 0.0350 | 0.598 | -1.673 |
| AK024480 | LOC126917 | XM_928886 | 0.0252 | 0.598 | -1.673 |
| AA535128 | C11orf74 | NM_138787 | 0.0378 | 0.599 | -1.671 |
| NM_002228 | JUN | NM_002228 | 0.0150 | 0.599 | -1.671 |
| NM_012302 | LPHN2 | NM_012302 | 0.0236 | 0.599 | -1.671 |
| AW009638 | LOC728377 | XM_001127355 | 0.0202 | 0.599 | -1.671 |
| AI073984 | IRF8 | NM_002163 | 0.0441 | 0.599 | -1.669 |
| N33174 |  |  | 0.0351 | 0.600 | -1.668 |
| NM_032773 | LRCH3 | NM_032773 | 0.0200 | 0.600 | -1.668 |
| NM_000173 | GP1BA | NM_000173 | 0.0261 | 0.600 | -1.667 |
| AV709406 | TMEM125 | NM_144626 | 0.0207 | 0.600 | -1.667 |
| AU149503 | G3BP2 | NM_012297 | 0.0113 | 0.600 | -1.667 |
| NM_025243 | SLC19A3 | NM_025243 | 0.0354 | 0.600 | -1.667 |
| AI628573 | FGFBP3 | NM_152429 | 0.0008 | 0.600 | -1.666 |
| BC017275 |  |  | 0.0324 | 0.600 | -1.666 |
| NM_018082 | POLR3B | NM_018082 | 0.0003 | 0.601 | -1.665 |
| BE538424 | WDR68 | NM_005828 | 0.0226 | 0.601 | -1.664 |
| AF249273 | BCLAF1 | NM_001077440 | 0.0193 | 0.601 | -1.663 |
| AI375486 | APC | NM_000038 | 0.0199 | 0.602 | -1.662 |
| AY034482 | SYNCRIP | NM_006372 | 0.0290 | 0.602 | -1.662 |
| N66622 |  |  | 0.0197 | 0.602 | -1.660 |
| AL031602 | MT1M | NM_176870 | 0.0303 | 0.603 | -1.659 |
| NM_001065 | TNFRSF1A | NM_001065 | 0.0314 | 0.604 | -1.657 |
| AA422049 | WIZ | NM_021241 | 0.0140 | 0.604 | -1.656 |
| AI375916 | TCF7L2 | NM_030756 | 0.0302 | 0.604 | -1.656 |
| BF576458 | NCOA1 | NM_003743 | 0.0012 | 0.605 | -1.652 |
| AI721172 | AARS2 | NM_020745 | 0.0328 | 0.605 | -1.652 |
| AW471145 | PRSS23 | NM_007173 | 0.0231 | 0.606 | -1.651 |
| M23254 | CAPN2 | NM_001748 | 0.0002 | 0.606 | -1.651 |
| AA527515 | FAM86B1 | NM_001083537 | 0.0451 | 0.606 | -1.651 |
| NM_025099 | C17orf68 | NM_025099 | 0.0402 | 0.606 | -1.651 |
| NM_005649 | ZNF354A | NM_005649 | 0.0207 | 0.606 | -1.651 |
| NM_030912 | TRIM8 | NM_030912 | 0.0259 | 0.606 | -1.650 |
| BF436101 |  |  | 0.0385 | 0.606 | -1.650 |
| AI743109 | TRIM41 | NM_033549 | 0.0310 | 0.606 | -1.650 |
| AW236976 | ZNF770 | NM_014106 | 0.0113 | 0.606 | -1.650 |
| AI829721 | LOC647859 | XM_001127102 | 0.0131 | 0.606 | -1.650 |
| AI022089 | CSNK2A2 | NM_001896 | 0.0254 | 0.607 | -1.649 |
| BC029474 |  |  | 0.0337 | 0.607 | -1.648 |
| BI832220 | C1orf53 | NM_001024594 | 0.0382 | 0.607 | -1.648 |
| NM_016357 | LIMA1 | NM_016357 | 0.0065 | 0.607 | -1.647 |
| AI028241 | DGCR8 | NM_022720 | 0.0248 | 0.607 | -1.647 |
| NM_021731 | C19orf28 | NM_001042680 | 0.0089 | 0.608 | -1.645 |
| AF161419 | ING3 | NM_019071 | 0.0252 | 0.608 | -1.644 |
| U66065 | GRB10 | NM_001001549 | 0.0182 | 0.609 | -1.643 |
| AI498126 | BTBD14B | NM_052876 | 0.0143 | 0.609 | -1.643 |
| AA121502 | HEG1 | NM_020733 | 0.0207 | 0.609 | -1.642 |
| AF085357 | FLOT1 | NM_005803 | 0.0230 | 0.610 | -1.640 |
| BC004988 | FEM1A | NM_018708 | 0.0193 | 0.610 | -1.639 |
| BF242537 | ALKBH8 | NM_138775 | 0.0029 | 0.610 | -1.638 |
| BG250721 | KLF6 | NM_001008490 | 0.0199 | 0.611 | -1.638 |
| AW263497 | SYTL5 |  | 0.0171 | 0.611 | -1.638 |
| AK025925 | WDR68 | NM_005828 | 0.0124 | 0.611 | -1.637 |
| W74620 | HNRPD | NM_001003810 | 0.0393 | 0.612 | -1.635 |
| AL354612 | TMEM48 | NM_018087 | 0.0096 | 0.612 | -1.635 |
| NM_022841 | RFXDC2 | NM_022841 | 0.0156 | 0.612 | -1.633 |
| AI458417 | LOC162073 | NM_001034841 | 0.0341 | 0.613 | -1.632 |
| T79216 | OTUD4 | NM_001102653 | 0.0368 | 0.613 | -1.632 |
| AK000752 | ERGIC1 | NM_001031711 | 0.0322 | 0.613 | -1.631 |
| AI458128 | CBX6 | NM_014292 | 0.0325 | 0.613 | -1.631 |
| AI272805 | SNX24 | NM_014035 | 0.0420 | 0.613 | -1.630 |
| AA541479 | MAP3K1 | NM_005921 | 0.0075 | 0.614 | -1.629 |
| AW629515 | VCPIP1 | NM_025054 | 0.0018 | 0.614 | -1.629 |
| BG260337 |  |  | 0.0164 | 0.614 | -1.628 |
| BC010363 | LINS1 | NM_001040614 | 0.0358 | 0.614 | -1.628 |
| NM_004841 | RASAL2 | NM_004841 | 0.0338 | 0.615 | -1.627 |
| NM_003567 | BCAR3 | NM_003567 | 0.0026 | 0.615 | -1.627 |
| BG168471 | MGLL | NM_001003794 | 0.0014 | 0.615 | -1.626 |
| AA728758 | C14orf65 |  | 0.0010 | 0.615 | -1.626 |
| AK091107 | C15orf37 | NM_175898 | 0.0050 | 0.615 | -1.625 |
| NM_024658 | IPO4 | NM_024658 | 0.0066 | 0.616 | -1.625 |
| BF131947 | WDR51B | NM_172240 | 0.0030 | 0.616 | -1.624 |
| NM_014289 | CAPN6 | NM_014289 | 0.0352 | 0.616 | -1.624 |
| BF439533 | FLJ32810 | XM_001127587 | 0.0189 | 0.616 | -1.623 |
| NM_025128 | MUS81 | NM_025128 | 0.0126 | 0.616 | -1.623 |
| BF196642 | UBE2D2 | NM_003339 | 0.0073 | 0.617 | -1.622 |
| NM_006506 | RASA2 | NM_006506 | 0.0105 | 0.617 | -1.620 |
| AF132818 | KLF5 | NM_001730 | 0.0001 | 0.618 | -1.618 |
| BG230586 | SLC7A6 | NM_001076785 | 0.0090 | 0.619 | -1.615 |
| R50822 | LPHN3 | NM_015236 | 0.0442 | 0.619 | -1.615 |
| NM_014016 | SACM1L | NM_014016 | 0.0032 | 0.619 | -1.614 |
| AL567808 | ZNF19 /// ZNF23 | NM_006961 | 0.0251 | 0.620 | -1.613 |
| NM_004635 | MAPKAPK3 | NM_004635 | 0.0404 | 0.620 | -1.613 |
| NM_001982 | ERBB3 | NM_001005915 | 0.0053 | 0.621 | -1.612 |
| NM_012297 | G3BP2 | NM_012297 | 0.0120 | 0.621 | -1.611 |
| NM_002428 | MMP15 | NM_002428 | 0.0038 | 0.621 | -1.611 |
| NM_002310 | LIFR | NM_002310 | 0.0417 | 0.621 | -1.610 |
| AF049103 | SETD2 | NM_014159 | 0.0044 | 0.622 | -1.609 |
| NM_018090 | NECAP2 | NM_018090 | 0.0275 | 0.622 | -1.608 |
| BC041094 | TAF5L | NM_001025247 | 0.0199 | 0.622 | -1.608 |
| R61374 | HEY1 | NM_001040708 | 0.0108 | 0.622 | -1.608 |
| NM_018324 | OLAH | NM_001039702 | 0.0486 | 0.622 | -1.607 |
| AF116707 | KIAA1147 | NM_001080392 | 0.0485 | 0.622 | -1.607 |
| AW967916 |  |  | 0.0017 | 0.622 | -1.607 |
| AK024051 | LRRC41 | NM_006369 | 0.0474 | 0.623 | -1.606 |
| BF184089 | ZDHHC21 | NM_178566 | 0.0256 | 0.623 | -1.605 |
| AI625235 | C20orf199 | NR_003604 | 0.0245 | 0.623 | -1.604 |
| AW576195 |  |  | 0.0105 | 0.624 | -1.604 |
| BF447954 | DOCK5 | NM_024940 | 0.0053 | 0.624 | -1.603 |
| AW235608 | TTC9 | NM_015351 | 0.0007 | 0.624 | -1.602 |
| AA706895 | ADAT2 | NM_182503 | 0.0109 | 0.624 | -1.602 |
| NM_018695 | ERBB2IP | NM_001006600 | 0.0030 | 0.624 | -1.602 |
| AW235061 | SLC1A1 | NM_004170 | 0.0194 | 0.624 | -1.602 |
| AA605121 |  |  | 0.0212 | 0.625 | -1.601 |
| BF576005 | FYTTD1 | NM_001011537 | 0.0032 | 0.625 | -1.600 |
| AF037448 | SYNCRIP | NM_006372 | 0.0341 | 0.625 | -1.599 |
| BG223334 | C9orf114 | NM_016390 | 0.0256 | 0.625 | -1.599 |
| AV722693 | ETV6 | NM_001987 | 0.0331 | 0.626 | -1.598 |
| AL038092 | ZNF134 | NM_003435 | 0.0306 | 0.626 | -1.598 |
| BC006236 | MAG1 | NM_032717 | 0.0034 | 0.626 | -1.598 |
| NM_024584 | CCDC121 | NM_024584 | 0.0462 | 0.627 | -1.596 |
| NM_017742 | ZCCHC2 | NM_017742 | 0.0397 | 0.627 | -1.595 |
| BC004995 | MARVELD1 | NM_031484 | 0.0086 | 0.627 | -1.594 |
| NM_004380 | CREBBP | NM_001079846 | 0.0253 | 0.628 | -1.594 |
| NM_004184 | WARS | NM_004184 | 0.0257 | 0.628 | -1.593 |
| AA001052 | RAB12 | NM_001025300 | 0.0095 | 0.628 | -1.592 |
| BE676543 | ZCCHC2 | NM_017742 | 0.0215 | 0.628 | -1.592 |
| NM_030672 | ARHGAP28 | NM_001010000 | 0.0426 | 0.628 | -1.592 |
| BF435769 | LOC646214 | XR_016124 | 0.0084 | 0.628 | -1.591 |
| BF064224 |  |  | 0.0286 | 0.629 | -1.591 |
| AW052119 | HOMER1 | NM_004272 | 0.0043 | 0.629 | -1.590 |
| BF512491 |  |  | 0.0331 | 0.629 | -1.590 |
| AF007217 | TRIP11 | NM_004239 | 0.0298 | 0.629 | -1.589 |
| AW294869 |  |  | 0.0043 | 0.629 | -1.589 |
| AL110209 | LYPLA3 | NM_012320 | 0.0005 | 0.629 | -1.589 |
| AL110131 | ANKRD50 | NM_020337 | 0.0187 | 0.629 | -1.589 |
| NM_016955 | SEPSECS | NM_016955 | 0.0487 | 0.630 | -1.588 |
| BC031487 | MGAT4A | NM_012214 | 0.0244 | 0.630 | -1.588 |
| R71157 | TRIM62 | NM_018207 | 0.0163 | 0.630 | -1.588 |
| BE564430 |  |  | 0.0009 | 0.630 | -1.587 |
| BF508843 | KIAA0907 | NM_014949 | 0.0322 | 0.630 | -1.586 |
| AI949549 | FGD4 | NM_139241 | 0.0220 | 0.630 | -1.586 |
| AL109658 | NSFL1C | NM_016143 | 0.0043 | 0.631 | -1.586 |
| AB028980 | USP24 | NM_015306 | 0.0188 | 0.631 | -1.586 |
| AB011113 | WDR7 | NM_015285 | 0.0288 | 0.631 | -1.586 |
| AV733308 | ITGA6 | NM_000210 | 0.0228 | 0.631 | -1.585 |
| AB047005 | MAST2 | NM_015112 | 0.0484 | 0.631 | -1.584 |
| AA988769 |  |  | 0.0335 | 0.632 | -1.584 |
| U35004 | MAPK8 | NM_002750 | 0.0004 | 0.632 | -1.583 |
| AK026691 | GUSBL2 | NM_206908 | 0.0482 | 0.632 | -1.583 |
| NM_017702 | DEF8 | NM_017702 | 0.0492 | 0.632 | -1.583 |
| BG260069 |  |  | 0.0044 | 0.632 | -1.583 |
| BG427809 | BMS1P5 | NR_003611 | 0.0218 | 0.633 | -1.581 |
| AL117653 | MITF | NM_000248 | 0.0220 | 0.633 | -1.580 |
| AI467947 | C3orf21 | NM_152531 | 0.0469 | 0.633 | -1.580 |
| NM_006717 | SPIN1 | NM_006717 | 0.0410 | 0.633 | -1.580 |
| AI521618 | TPM1 | NM_000366 | 0.0447 | 0.633 | -1.579 |
| AK027217 | PDLIM5 | NM_001011513 | 0.0493 | 0.633 | -1.579 |
| NM_005886 | KATNB1 | NM_005886 | 0.0067 | 0.633 | -1.579 |
| AA504249 |  |  | 0.0473 | 0.634 | -1.579 |
| AI016784 | ZNF148 | NM_021964 | 0.0007 | 0.634 | -1.578 |
| AL574660 | ABCD4 | NM_005050 | 0.0369 | 0.634 | -1.577 |
| NM_018087 | TMEM48 | NM_018087 | 0.0013 | 0.634 | -1.577 |
| AK074354 | BTBD7 | NM_001002860 | 0.0133 | 0.635 | -1.576 |
| NM_024772 | ZMYM1 | NM_024772 | 0.0062 | 0.635 | -1.576 |
| NM_002467 | MYC | NM_002467 | 0.0159 | 0.635 | -1.576 |
| D87811 | GATA6 | NM_005257 | 0.0117 | 0.635 | -1.575 |
| BC005369 | EGLN1 | NM_022051 | 0.0344 | 0.636 | -1.573 |
| AI703074 | TCF7L2 | NM_030756 | 0.0309 | 0.636 | -1.572 |
| BE856374 | USP46 | NM_022832 | 0.0065 | 0.636 | -1.572 |
| U41815 | NUP98 | NM_005387 | 0.0369 | 0.637 | -1.571 |
| AB007830 | SCARA3 | NM_016240 | 0.0104 | 0.637 | -1.570 |
| AF081567 | PRKRIR | NM_004705 | 0.0159 | 0.637 | -1.570 |
| NM_024790 | CSPP1 | NM_001077204 | 0.0030 | 0.637 | -1.570 |
| NM_003387 | WIPF1 | NM_001077269 | 0.0385 | 0.637 | -1.569 |
| NM_005716 | GIPC1 | NM_005716 | 0.0331 | 0.638 | -1.569 |
| AA417970 | ZNF621 | NM_001098414 | 0.0048 | 0.638 | -1.568 |
| AA609488 | CHDH | NM_018397 | 0.0040 | 0.638 | -1.567 |
| BE881219 | ATPAF1 | NM_001042546 | 0.0078 | 0.638 | -1.567 |
| N35244 | ZNF782 | NM_001001662 | 0.0242 | 0.638 | -1.567 |
| BC031345 |  |  | 0.0398 | 0.638 | -1.567 |
| NM_016358 | IRX4 | NM_016358 | 0.0167 | 0.638 | -1.566 |
| N79601 |  |  | 0.0248 | 0.639 | -1.566 |
| AA706480 | LOC286260 | XM_926851 | 0.0288 | 0.639 | -1.566 |
| N21008 | ZYG11B | NM_024646 | 0.0227 | 0.639 | -1.565 |
| BF574430 | RAB12 | NM_001025300 | 0.0358 | 0.639 | -1.564 |
| AK002110 | NDUFS8 | NM_002496 | 0.0061 | 0.640 | -1.564 |
| NM_032876 | JUB | NM_032876 | 0.0368 | 0.640 | -1.563 |
| BE207758 | ARRB1 | NM_004041 | 0.0139 | 0.640 | -1.563 |
| NM_031283 | TCF7L1 | NM_031283 | 0.0431 | 0.640 | -1.562 |
| AK024516 |  |  | 0.0473 | 0.640 | -1.562 |
| BE000929 | MSI2 | NM_138962 | 0.0335 | 0.641 | -1.561 |
| AW079553 |  |  | 0.0098 | 0.641 | -1.560 |
| BQ899060 |  |  | 0.0453 | 0.641 | -1.559 |
| NM_004834 | MAP4K4 | NM_004834 | 0.0128 | 0.642 | -1.559 |
| NM_004353 | SERPINH1 | NM_001235 | 0.0467 | 0.642 | -1.558 |
| AK025567 | JUB | NM_032876 | 0.0008 | 0.642 | -1.557 |
| AW102941 | FARP1 | NM_001001715 | 0.0310 | 0.643 | -1.556 |
| BQ433060 | ZNF642 | NM_198494 | 0.0023 | 0.643 | -1.555 |
| AI888256 | RNF217 | NM_152553 | 0.0026 | 0.643 | -1.555 |
| NM_014160 | MKRN2 | NM_014160 | 0.0223 | 0.644 | -1.554 |
| AI872645 | DNAH5 | NM_001369 | 0.0261 | 0.644 | -1.554 |
| BG036203 | LOC203547 | NM_001017980 | 0.0328 | 0.644 | -1.554 |
| NM_017515 | SLC35F2 | NM_017515 | 0.0018 | 0.644 | -1.553 |
| BC015343 | LOC162073 | NM_001034841 | 0.0380 | 0.645 | -1.550 |
| NM_001566 | INPP4A | NM_001566 | 0.0320 | 0.645 | -1.550 |
| AA678492 | C9orf100 | NM_032818 | 0.0115 | 0.646 | -1.549 |
| BF696931 | CCDC50 | NM_174908 | 0.0166 | 0.646 | -1.548 |
| AW204088 | DCP1B | NM_152640 | 0.0341 | 0.646 | -1.548 |
| AI916242 | EEA1 | NM_003566 | 0.0331 | 0.646 | -1.547 |
| NM_007357 | COG2 | NM_007357 | 0.0204 | 0.647 | -1.546 |
| BC000050 | NOB1 | NM_014062 | 0.0215 | 0.647 | -1.545 |
| U47635 | MTMR6 | NM_004685 | 0.0185 | 0.647 | -1.545 |
| AK025444 | PHLDB2 | NM_145753 | 0.0139 | 0.648 | -1.544 |
| BF339566 | NAV1 | NM_020443 | 0.0152 | 0.648 | -1.543 |
| NM_003489 | NRIP1 | NM_003489 | 0.0138 | 0.648 | -1.542 |
| AI660619 | SLC7A6 | NM_001076785 | 0.0346 | 0.649 | -1.542 |
| AA631242 | RAB15 | NM_198686 | 0.0276 | 0.649 | -1.542 |
| BE671084 | ARHGAP26 | NM_015071 | 0.0049 | 0.649 | -1.541 |
| AI743903 | FLJ39051 |  | 0.0086 | 0.649 | -1.540 |
| AI912523 | KIAA1430 | NM_020827 | 0.0022 | 0.650 | -1.539 |
| BE550452 | HOMER1 | NM_004272 | 0.0055 | 0.650 | -1.539 |
| AA129776 | SUOX | NM_000456 | 0.0254 | 0.650 | -1.538 |
| AA278233 | LOC286052 |  | 0.0246 | 0.650 | -1.538 |
| BC000915 | PDLIM1 | NM_020992 | 0.0045 | 0.650 | -1.538 |
| NM_006584 | CCT6B | NM_006584 | 0.0275 | 0.650 | -1.538 |
| NM_003878 | GGH | NM_003878 | 0.0067 | 0.651 | -1.537 |
| AW138594 | KLHL9 | NM_018847 | 0.0350 | 0.651 | -1.536 |
| NM_024834 | C10orf119 | NM_024834 | 0.0156 | 0.651 | -1.536 |
| BF213575 | EPS15 | NM_001981 | 0.0264 | 0.651 | -1.536 |
| BC000251 | GSK3B | NM_002093 | 0.0200 | 0.651 | -1.536 |
| AW500220 | KCTD20 | NM_173562 | 0.0106 | 0.651 | -1.536 |
| NM_012201 | GLG1 | NM_012201 | 0.0383 | 0.651 | -1.535 |
| AW029619 | CKAP4 | NM_006825 | 0.0184 | 0.652 | -1.534 |
| BE927772 | SFRS3 | NM_003017 | 0.0304 | 0.652 | -1.533 |
| AI652633 |  |  | 0.0414 | 0.652 | -1.533 |
| BE379393 | C6orf132 | XM_371820 | 0.0479 | 0.652 | -1.533 |
| D21851 | LARS2 | NM_015340 | 0.0188 | 0.652 | -1.533 |
| AL832682 | PARVA | NM_018222 | 0.0020 | 0.653 | -1.532 |
| BF438203 | ZXDC | NM_001040653 | 0.0499 | 0.653 | -1.532 |
| NM_000188 | HK1 | NM_000188 | 0.0348 | 0.653 | -1.531 |
| AA531337 | CRIPAK | NM_175918 | 0.0324 | 0.654 | -1.530 |
| BC001327 | IFRD2 | NM_006764 | 0.0163 | 0.654 | -1.530 |
| AI743880 |  |  | 0.0235 | 0.654 | -1.529 |
| R85437 | VANGL1 | NM_138959 | 0.0044 | 0.654 | -1.528 |
| BC013077 |  |  | 0.0015 | 0.655 | -1.528 |
| BC040723 | AFAP1L1 | NM_152406 | 0.0216 | 0.655 | -1.528 |
| BC004490 | FOS | NM_005252 | 0.0239 | 0.655 | -1.527 |
| AL562733 | ERAL1 | NM_005702 | 0.0341 | 0.655 | -1.527 |
| N58163 | WDR32 | NM_024345 | 0.0078 | 0.655 | -1.526 |
| BE879367 | AKAP2 /// PALM2-AKAP2 | NM_001004065 | 0.0256 | 0.655 | -1.526 |
| AI652645 | IQSEC1 | NM_014869 | 0.0135 | 0.656 | -1.525 |
| AI184512 | THEM4 | NM_053055 | 0.0167 | 0.656 | -1.525 |
| BU683415 | KLF6 | NM_001008490 | 0.0154 | 0.656 | -1.524 |
| NM_024121 | TMEM185B | NR_000034 | 0.0092 | 0.657 | -1.523 |
| AI742039 | OGT | NM_181672 | 0.0455 | 0.657 | -1.522 |
| AA582932 | RAB15 | NM_198686 | 0.0309 | 0.657 | -1.522 |
| AU157304 | C3orf59 | NM_178496 | 0.0044 | 0.658 | -1.520 |
| BF001312 | EEF2K | NM_013302 | 0.0173 | 0.658 | -1.520 |
| BE501789 | NSL1 | NM_001042549 | 0.0253 | 0.658 | -1.520 |
| BE858180 | PEG10 | NM_001040152 | 0.0024 | 0.659 | -1.519 |
| AW952547 | MDH1 | NM_005917 | 0.0422 | 0.659 | -1.519 |
| AW593330 |  |  | 0.0038 | 0.659 | -1.518 |
| AA960804 | LOC728613 | NR_003713 | 0.0150 | 0.659 | -1.518 |
| AA481141 | VAV2 | NM_003371 | 0.0365 | 0.659 | -1.517 |
| NM_018117 | BRWD2 | NM_018117 | 0.0114 | 0.659 | -1.517 |
| AI769569 | MAML2 | NM_032427 | 0.0130 | 0.660 | -1.516 |
| BE552097 | PWWP2A | NM_052927 | 0.0491 | 0.660 | -1.516 |
| AA148301 | COMMD7 | NM_001099339 | 0.0273 | 0.660 | -1.516 |
| AL046979 | TNS1 | NM_022648 | 0.0096 | 0.660 | -1.515 |
| AA829836 | C9orf126 | NM_173690 | 0.0123 | 0.660 | -1.515 |
| BC005821 | PTEN | NM_000314 | 0.0076 | 0.660 | -1.514 |
| NM_012179 | FBXO7 | NM_001033024 | 0.0329 | 0.661 | -1.514 |
| NM_004125 | GNG10 /// LOC552891 | NM_001017998 | 0.0261 | 0.661 | -1.513 |
| AV704797 | KIAA1549 | NM_020910 | 0.0460 | 0.661 | -1.513 |
| T58129 | HUNK | NM_014586 | 0.0330 | 0.662 | -1.511 |
| NM_015623 | TANC2 | NM_025185 | 0.0279 | 0.662 | -1.511 |
| NM_004034 | ANXA7 | NM_001156 | 0.0260 | 0.662 | -1.511 |
| NM_004865 | TBPL1 | NM_004865 | 0.0124 | 0.663 | -1.509 |
| BG286920 | RSF1 | NM_016578 | 0.0270 | 0.663 | -1.509 |
| AF119841 | PECR | NM_018441 | 0.0219 | 0.663 | -1.508 |
| BE049621 | LUC7L | NM_018032 | 0.0456 | 0.663 | -1.507 |
| AL117352 | EGLN1 | NM_022051 | 0.0300 | 0.664 | -1.507 |
| AA234096 | MGC16121 | XM_001128419 | 0.0406 | 0.664 | -1.507 |
| AK023513 | SNAPC4 | NM_003086 | 0.0417 | 0.664 | -1.507 |
| BG540494 | AKAP2 /// PALM2-AKAP2 | NM_001004065 | 0.0063 | 0.664 | -1.506 |
| AI623211 | LOC645166 | XM_001129441 | 0.0498 | 0.664 | -1.506 |
| AW593801 |  |  | 0.0466 | 0.664 | -1.506 |
| AI949095 | FAM83H | NM_198488 | 0.0320 | 0.665 | -1.505 |
| AF272663 | GPHN | NM_001024218 | 0.0325 | 0.665 | -1.504 |
| NM_014612 | FAM120A | NM_014612 | 0.0469 | 0.665 | -1.504 |
| AF084513 | RAD1 | NM_002853 | 0.0319 | 0.665 | -1.503 |
| NM_005842 | SPRY2 | NM_005842 | 0.0349 | 0.666 | -1.502 |
| BE048857 | VPS13B | NM_015243 | 0.0084 | 0.666 | -1.501 |
| NM_017615 | NSMCE4A | NM_017615 | 0.0108 | 0.666 | -1.501 |
| AF083105 | SOX13 | NM_005686 | 0.0098 | 0.666 | -1.501 |
| AA741090 | CALML4 | NM_001031733 | 0.0234 | 0.667 | -1.500 |
| BC013132 | GDAP2 | NM_017686 | 0.0209 | 0.667 | -1.500 |
| AL078459 | DDAH1 | NM_012137 | 0.0028 | 0.667 | -1.499 |
| BG289967 | RAD21 | NM_006265 | 0.0164 | 0.667 | -1.499 |
| NM_024098 | CCDC86 | NM_024098 | 0.0063 | 0.667 | -1.499 |
| NM_001270 | CHD1 | NM_001270 | 0.0214 | 0.668 | -1.498 |
| AI459274 | ZFR | NM_016107 | 0.0436 | 0.668 | -1.497 |
| AW469181 | TMC5 | NM_001105248 | 0.0275 | 0.668 | -1.497 |
| NM_001323 | CST6 | NM_001323 | 0.0449 | 0.669 | -1.496 |
| AA526844 | MYLK | NM_005965 | 0.0404 | 0.669 | -1.494 |
| AF161528 | NIP7 | NM_016101 | 0.0346 | 0.670 | -1.493 |
| NM_000698 | ALOX5 | NM_000698 | 0.0347 | 0.670 | -1.493 |
| AI672489 |  |  | 0.0365 | 0.670 | -1.492 |
| NM_001386 | DPYSL2 | NM_001386 | 0.0123 | 0.671 | -1.491 |
| AI241810 |  |  | 0.0266 | 0.671 | -1.491 |
| AI807206 | ZDHHC21 | NM_178566 | 0.0164 | 0.671 | -1.490 |
| AI130715 | CEP152 | NM_014985 | 0.0478 | 0.672 | -1.489 |
| AL832823 | HS3ST3B1 | NM_006041 | 0.0270 | 0.672 | -1.489 |
| AI356895 | RHBDD1 | NM_032276 | 0.0174 | 0.672 | -1.489 |
| AA909035 | COL4A2 | NM_001846 | 0.0145 | 0.672 | -1.488 |
| BE549973 | UBA5 | NM_024818 | 0.0307 | 0.672 | -1.488 |
| AU147713 | SRRM1 | NM_005839 | 0.0119 | 0.672 | -1.488 |
| NM_005808 | CTDSPL | NM_001008392 | 0.0065 | 0.673 | -1.486 |
| M58596 | FUT4 | NM_002033 | 0.0205 | 0.673 | -1.486 |
| H37943 |  |  | 0.0369 | 0.673 | -1.485 |
| AL136782 | KBTBD7 | NM_032138 | 0.0376 | 0.674 | -1.485 |
| U90902 | TIAM1 | NM_003253 | 0.0233 | 0.674 | -1.483 |
| AF305057 | ENOSF1 | NM_017512 | 0.0151 | 0.674 | -1.483 |
| AF020543 | PPT2 | NM_005155 | 0.0482 | 0.674 | -1.483 |
| AI590926 | SLC35B4 | NM_032826 | 0.0111 | 0.674 | -1.483 |
| AB011100 | KIAA0528 | NM_014802 | 0.0107 | 0.675 | -1.481 |
| AI762884 |  |  | 0.0306 | 0.675 | -1.481 |
| NM_001821 | CHML | NM_001821 | 0.0118 | 0.676 | -1.480 |
| AW151538 | C21orf45 | NM_018944 | 0.0428 | 0.676 | -1.480 |
| NM_004504 | HRB | NM_004504 | 0.0029 | 0.676 | -1.480 |
| AW195407 | C10orf30 | NM_001100912 | 0.0378 | 0.676 | -1.479 |
| AL045882 | PCGF5 | NM_032373 | 0.0300 | 0.676 | -1.479 |
| AK027737 | PRMT5 | NM_001039619 | 0.0072 | 0.677 | -1.477 |
| NM_001649 | SHROOM2 | NM_001649 | 0.0074 | 0.677 | -1.477 |
| BC003697 | KCTD20 | NM_173562 | 0.0134 | 0.677 | -1.477 |
| BE328496 | MBNL2 | NM_144778 | 0.0099 | 0.677 | -1.477 |
| BF214358 | FAM76B | NM_144664 | 0.0393 | 0.677 | -1.477 |
| NM_005544 | IRS1 | NM_005544 | 0.0195 | 0.678 | -1.475 |
| AI680541 | LIFR | NM_002310 | 0.0480 | 0.678 | -1.475 |
| AL359939 | VPS54 | NM_001005739 | 0.0055 | 0.678 | -1.475 |
| AF362887 | TPM4 | NM_003290 | 0.0419 | 0.678 | -1.474 |
| Y11162 | SNORA68 | NR_000012 | 0.0489 | 0.678 | -1.474 |
| AA007367 | FAM109A | NM_144671 | 0.0047 | 0.678 | -1.474 |
| AA195024 | LONP2 | NM_031490 | 0.0477 | 0.679 | -1.474 |
| BF439282 | RAPGEF2 | NM_014247 | 0.0371 | 0.679 | -1.472 |
| AV721430 | TCF7L2 | NM_030756 | 0.0119 | 0.680 | -1.471 |
| AU152194 | LOC388720 /// RPS27A | NM_002954 | 0.0321 | 0.681 | -1.468 |
| BF217861 | MT1E | NM_175617 | 0.0321 | 0.681 | -1.468 |
| AF085969 |  |  | 0.0255 | 0.682 | -1.467 |
| BF197122 | KIAA0090 | NM_015047 | 0.0128 | 0.682 | -1.466 |
| AI708524 | LOC440552 |  | 0.0213 | 0.682 | -1.466 |
| BG532405 | C13orf1 | NM_020456 | 0.0163 | 0.682 | -1.466 |
| AI400463 | CTGLF3 |  | 0.0068 | 0.682 | -1.465 |
| BC001161 | ZNF174 | NM_001032292 | 0.0343 | 0.683 | -1.465 |
| N64780 | ASXL1 | NM_015338 | 0.0242 | 0.683 | -1.465 |
| BE221212 | COL1A1 | NM_000088 | 0.0090 | 0.683 | -1.465 |
| AI554467 | LOC388344 /// RPL13 /// SNORD68 | NM_000977 | 0.0302 | 0.683 | -1.465 |
| AK026630 | C10orf84 | NM_022063 | 0.0212 | 0.683 | -1.464 |
| NM_006090 | CEPT1 | NM_001007794 | 0.0277 | 0.683 | -1.464 |
| NM_152484 | ZNF569 | NM_152484 | 0.0221 | 0.684 | -1.463 |
| NM_014942 | ANKRD6 | NM_014942 | 0.0440 | 0.684 | -1.463 |
| U94363 | GYG2 | NM_001079855 | 0.0482 | 0.684 | -1.463 |
| AK001380 | ASPM | NM_018136 | 0.0324 | 0.684 | -1.462 |
| AI382123 | MYH10 | NM_005964 | 0.0071 | 0.684 | -1.462 |
| NM_006296 | VRK2 | NM_006296 | 0.0043 | 0.684 | -1.462 |
| BC004234 | LONP2 | NM_031490 | 0.0168 | 0.685 | -1.460 |
| AL136776 | MED23 | NM_004830 | 0.0201 | 0.685 | -1.460 |
| NM_000951 | PRRG2 | NM_000951 | 0.0127 | 0.685 | -1.460 |
| AB014560 | G3BP2 | NM_012297 | 0.0475 | 0.685 | -1.460 |
| NM_001943 | DSG2 | NM_001943 | 0.0323 | 0.685 | -1.460 |
| NM_020674 | CYP20A1 | NM_020674 | 0.0266 | 0.685 | -1.459 |
| BF590317 | CTDSPL | NM_001008392 | 0.0356 | 0.686 | -1.459 |
| BE503981 |  |  | 0.0473 | 0.686 | -1.458 |
| NM_018127 | ELAC2 | NM_018127 | 0.0067 | 0.687 | -1.456 |
| AF033861 | ADCY3 | NM_004036 | 0.0405 | 0.687 | -1.456 |
| BF058944 | SCAMP1 | NM_004866 | 0.0276 | 0.687 | -1.455 |
| BF001666 | FBXL14 | NM_152441 | 0.0061 | 0.687 | -1.455 |
| AA121673 | ZNF281 | NM_012482 | 0.0091 | 0.688 | -1.454 |
| AU137607 | NAV2 | NM_001111018 | 0.0474 | 0.688 | -1.454 |
| BF432532 | TIAL1 | NM_001033925 | 0.0280 | 0.688 | -1.453 |
| NM_014873 | LPGAT1 | NM_014873 | 0.0256 | 0.689 | -1.452 |
| BC032942 | GSTCD | NM_001031720 | 0.0144 | 0.689 | -1.452 |
| NM_003144 | SSR1 | NM_003144 | 0.0203 | 0.689 | -1.451 |
| NM_002184 | IL6ST | NM_002184 | 0.0155 | 0.689 | -1.451 |
| AU155298 | CHD1 | NM_001270 | 0.0064 | 0.690 | -1.449 |
| AL117518 | ASXL1 | NM_015338 | 0.0293 | 0.690 | -1.449 |
| NM_173709 |  |  | 0.0047 | 0.690 | -1.449 |
| AI636233 | TMEM8 | NM_021259 | 0.0464 | 0.691 | -1.447 |
| AI291720 | DPH5 | NM_001077394 | 0.0236 | 0.691 | -1.447 |
| AK024300 | MGC45800 | XR_017723 | 0.0038 | 0.691 | -1.447 |
| BE621259 | UBE2D2 | NM_003339 | 0.0219 | 0.691 | -1.447 |
| NM_006470 | TRIM16 /// TRIM16L | NM_001037330 | 0.0320 | 0.691 | -1.447 |
| NM_020380 | CASC5 | NM_144508 | 0.0449 | 0.692 | -1.446 |
| NM_021930 | RINT1 | NM_021930 | 0.0321 | 0.692 | -1.445 |
| AW271106 | IQGAP3 | NM_178229 | 0.0315 | 0.692 | -1.444 |
| NM_012204 | GTF3C4 | NM_012204 | 0.0363 | 0.693 | -1.444 |
| AI694332 | ARIH1 | NM_005744 | 0.0284 | 0.693 | -1.443 |
| AW188170 |  |  | 0.0017 | 0.693 | -1.443 |
| AB051513 | ZC3H12C | NM_033390 | 0.0065 | 0.693 | -1.443 |
| AK027737 | PRMT5 | NM_001039619 | 0.0166 | 0.694 | -1.442 |
| AI912190 |  |  | 0.0255 | 0.694 | -1.442 |
| AA807529 | MCM5 | NM_006739 | 0.0174 | 0.694 | -1.442 |
| NM_005246 | FER | NM_005246 | 0.0030 | 0.694 | -1.441 |
| BF732413 | SEC22C | NM_004206 | 0.0476 | 0.694 | -1.441 |
| AL122121 | PAPD1 | NM_018109 | 0.0241 | 0.694 | -1.441 |
| AI650892 | NSUN4 | NM_199044 | 0.0330 | 0.694 | -1.441 |
| BF223021 | B4GALT4 | NM_003778 | 0.0160 | 0.695 | -1.440 |
| AI334297 | KLHDC5 | NM_020782 | 0.0324 | 0.695 | -1.439 |
| NM_004901 | ENTPD4 | NM_004901 | 0.0199 | 0.695 | -1.439 |
| BG026723 | QSER1 | NM_001076786 | 0.0283 | 0.695 | -1.439 |
| AI761110 | SETD2 | NM_014159 | 0.0500 | 0.695 | -1.438 |
| AK022897 | RECK | NM_021111 | 0.0343 | 0.695 | -1.438 |
| BG257762 | CDV3 | NM_017548 | 0.0315 | 0.696 | -1.438 |
| NM_006747 | SIPA1 | NM_006747 | 0.0465 | 0.696 | -1.438 |
| AI769587 | ARHGAP27 | NM_199282 | 0.0002 | 0.696 | -1.436 |
| BE892293 |  |  | 0.0409 | 0.697 | -1.436 |
| AK000776 |  |  | 0.0463 | 0.697 | -1.436 |
| AF167343 | IL1RAP | NM_002182 | 0.0247 | 0.697 | -1.436 |
| AV700030 | IL6R | NM_000565 | 0.0484 | 0.697 | -1.435 |
| AB032977 | NAV1 | NM_020443 | 0.0002 | 0.697 | -1.435 |
| AW613203 | PAIP1 | NM_006451 | 0.0067 | 0.697 | -1.434 |
| NM_012230 | POMZP3 /// ZP3 | NM_001110354 | 0.0339 | 0.698 | -1.433 |
| NM_014924 | KIAA0831 | NM_014924 | 0.0206 | 0.698 | -1.432 |
| BC041487 |  |  | 0.0168 | 0.698 | -1.432 |
| AI359368 | LETM1 | NM_012318 | 0.0252 | 0.698 | -1.432 |
| N93774 | C21orf45 | NM_018944 | 0.0183 | 0.698 | -1.432 |
| AF320070 | EHD4 | NM_139265 | 0.0403 | 0.699 | -1.431 |
| NM_024622 | FASTKD1 | NM_024622 | 0.0160 | 0.699 | -1.431 |
| BC000376 | ZFR | NM_016107 | 0.0384 | 0.699 | -1.430 |
| AI082078 | ACTN1 | NM_001102 | 0.0152 | 0.699 | -1.430 |
| AI379751 |  |  | 0.0364 | 0.700 | -1.429 |
| BG289456 | USP31 | NM_020718 | 0.0423 | 0.700 | -1.429 |
| NM_000268 | NF2 | NM_000268 | 0.0398 | 0.700 | -1.429 |
| AA664258 | HNRNPC | NM_001077442 | 0.0104 | 0.701 | -1.427 |
| AK001821 | GNPTAB | NM_024312 | 0.0128 | 0.702 | -1.425 |
| NM_018256 | WDR12 | NM_018256 | 0.0261 | 0.702 | -1.424 |
| AU157049 | LOC153346 |  | 0.0488 | 0.702 | -1.424 |
| NM_012343 | NNT | NM_012343 | 0.0049 | 0.702 | -1.424 |
| AK094809 | RASGRF2 | NM_006909 | 0.0064 | 0.703 | -1.423 |
| BF435773 | SHANK2 | NM_012309 | 0.0223 | 0.703 | -1.423 |
| AW515443 | NUCKS1 | NM_022731 | 0.0219 | 0.703 | -1.423 |
| AK074161 | SLC46A1 | NM_080669 | 0.0072 | 0.703 | -1.423 |
| AA740875 | GSTCD | NM_001031720 | 0.0498 | 0.703 | -1.423 |
| BF966015 | ZNF18 | NM_144680 | 0.0150 | 0.703 | -1.423 |
| NM_144692 | C19orf55 | NM_001039887 | 0.0222 | 0.703 | -1.423 |
| AW014593 | GBP1 | NM_002053 | 0.0212 | 0.703 | -1.422 |
| NM_016653 | ZAK | NM_016653 | 0.0490 | 0.704 | -1.421 |
| U13261 | METAP2 | NM_006838 | 0.0202 | 0.704 | -1.420 |
| AI391443 | SRFBP1 | NM_152546 | 0.0108 | 0.705 | -1.418 |
| BF444916 | FNDC3B | NM_022763 | 0.0157 | 0.706 | -1.417 |
| AI830698 | IGF1R | NM_000875 | 0.0184 | 0.707 | -1.415 |
| AL044018 | LPP | NM_005578 | 0.0203 | 0.707 | -1.415 |
| AF212224 | CLK4 | NM_020666 | 0.0051 | 0.707 | -1.414 |
| NM_005195 | CEBPD | NM_005195 | 0.0061 | 0.707 | -1.414 |
| AJ278245 | LANCL2 | NM_018697 | 0.0104 | 0.707 | -1.414 |
| AL049285 |  |  | 0.0241 | 0.707 | -1.414 |
| BC002666 | GBP1 | NM_002053 | 0.0476 | 0.708 | -1.413 |
| AI821787 |  |  | 0.0381 | 0.708 | -1.412 |
| AA209239 | ABHD6 | NM_020676 | 0.0214 | 0.708 | -1.412 |
| AL031313 | EIF3J /// LOC730021 | NM_003758 | 0.0321 | 0.709 | -1.411 |
| AL080111 | NEK7 | NM_133494 | 0.0272 | 0.709 | -1.411 |
| AI049962 | ZCCHC11 | NM_001009881 | 0.0143 | 0.709 | -1.411 |
| AI832363 |  |  | 0.0305 | 0.710 | -1.409 |
| AI888150 | PPP1R9A | NM_017650 | 0.0148 | 0.710 | -1.408 |
| BC000212 | GTF3C2 | NM_001035521 | 0.0402 | 0.710 | -1.408 |
| BC000853 | C2orf3 | NM_003203 | 0.0214 | 0.711 | -1.407 |
| NM_017555 | EGLN2 | NM_017555 | 0.0271 | 0.711 | -1.407 |
| AA648913 | BIRC5 | NM_001012270 | 0.0259 | 0.711 | -1.407 |
| D26069 | CENTB2 | NM_012287 | 0.0185 | 0.712 | -1.405 |
| AW001101 | KIAA0368 | NM_001080398 | 0.0137 | 0.712 | -1.405 |
| AL575922 | SPARC | NM_003118 | 0.0344 | 0.712 | -1.404 |
| AU157155 | AMOTL1 | NM_130847 | 0.0010 | 0.712 | -1.404 |
| AB044661 | XAB1 | NM_007266 | 0.0235 | 0.712 | -1.404 |
| AA524536 | LGR6 | NM_001017403 | 0.0133 | 0.713 | -1.403 |
| BC004517 | MRPL9 | NM_031420 | 0.0108 | 0.713 | -1.403 |
| AL359571 | NIN | NM_016350 | 0.0288 | 0.713 | -1.403 |
| BG105365 | SKP2 | NM_005983 | 0.0098 | 0.713 | -1.403 |
| AU149225 | MGA | NM_001080541 | 0.0150 | 0.714 | -1.401 |
| AW009330 | C13orf1 | NM_020456 | 0.0315 | 0.714 | -1.401 |
| AL045513 | POFUT1 | NM_015352 | 0.0048 | 0.714 | -1.400 |
| NM_024657 | MORC4 | NM_001085354 | 0.0098 | 0.714 | -1.400 |
| AV699857 | RPE | NM_006916 | 0.0024 | 0.714 | -1.400 |
| AI431597 | WDR22 | NM_003861 | 0.0096 | 0.715 | -1.400 |
| AK024986 | PTEN | NM_000314 | 0.0232 | 0.715 | -1.399 |
| NM_018443 | ZNF302 | NM_001012320 | 0.0089 | 0.715 | -1.399 |
| NM_022766 | CERK | NM_022766 | 0.0009 | 0.715 | -1.399 |
| NM_006800 | MSL3L1 | NM_006800 | 0.0478 | 0.715 | -1.399 |
| NM_003079 | SMARCE1 | NM_003079 | 0.0359 | 0.715 | -1.399 |
| H95263 | STX2 | NM_001980 | 0.0431 | 0.715 | -1.398 |
| AI674915 |  |  | 0.0429 | 0.715 | -1.398 |
| AL575509 | ETS2 | NM_005239 | 0.0121 | 0.715 | -1.398 |
| NM_017688 | BSPRY | NM_017688 | 0.0185 | 0.715 | -1.398 |
| NM_015198 | COBL | NM_015198 | 0.0336 | 0.715 | -1.398 |
| NM_014832 | TBC1D4 | NM_014832 | 0.0346 | 0.716 | -1.397 |
| BF515963 | WARS2 | NM_015836 | 0.0081 | 0.716 | -1.397 |
| AL552001 | PRKAB2 | NM_005399 | 0.0283 | 0.716 | -1.397 |
| AF044286 | H2AFY | NM_001040158 | 0.0092 | 0.716 | -1.396 |
| AB037732 | RBM27 |  | 0.0205 | 0.718 | -1.394 |
| NM_012338 | TSPAN12 | NM_012338 | 0.0017 | 0.718 | -1.394 |
| AI800609 | STX17 | NM_017919 | 0.0414 | 0.718 | -1.393 |
| BC004558 | RTKN | NM_001015055 | 0.0020 | 0.718 | -1.392 |
| AA528140 | DDIT4L | NM_145244 | 0.0210 | 0.718 | -1.392 |
| NM_016056 | TMBIM4 | NM_016056 | 0.0191 | 0.719 | -1.392 |
| AI346432 | MINA | NM_001042533 | 0.0203 | 0.719 | -1.390 |
| AW157070 | EGFR | NM_005228 | 0.0409 | 0.720 | -1.389 |
| NM_005754 | G3BP1 | NM_005754 | 0.0285 | 0.720 | -1.389 |
| AL046696 | BMPR2 | NM_001204 | 0.0414 | 0.720 | -1.388 |
| NM_001450 | FHL2 | NM_001039492 | 0.0499 | 0.720 | -1.388 |
| AL117643 | ACVR1B | NM_004302 | 0.0067 | 0.720 | -1.388 |
| NM_005154 | USP8 | NM_005154 | 0.0150 | 0.721 | -1.388 |
| AK022530 | DNAJC16 | NM_015291 | 0.0440 | 0.721 | -1.387 |
| BC005107 | C21orf105 |  | 0.0334 | 0.722 | -1.385 |
| NM_003630 | PEX3 | NM_003630 | 0.0184 | 0.724 | -1.382 |
| AU151801 | C1QBP | NM_001212 | 0.0437 | 0.724 | -1.382 |
| BG168720 | ZDHHC18 | NM_032283 | 0.0030 | 0.724 | -1.381 |
| AV724783 | PRDM2 | NM_001007257 | 0.0376 | 0.724 | -1.381 |
| AL390164 | GATAD2A | NM_017660 | 0.0303 | 0.725 | -1.380 |
| W93584 | POLR1A | NM_015425 | 0.0470 | 0.725 | -1.380 |
| AA235202 | WDR36 | NM_139281 | 0.0419 | 0.725 | -1.380 |
| AA521508 | ZMYM4 | NM_005095 | 0.0172 | 0.725 | -1.380 |
| AA825925 |  |  | 0.0371 | 0.725 | -1.379 |
| AI935647 | ARHGAP28 | NM_001010000 | 0.0230 | 0.726 | -1.378 |
| AK002205 | VPS54 | NM_001005739 | 0.0212 | 0.726 | -1.377 |
| BC003525 | MAX | NM_002382 | 0.0179 | 0.726 | -1.377 |
| BC001745 | D4S234E | NM_001040101 | 0.0067 | 0.727 | -1.376 |
| N90719 |  |  | 0.0123 | 0.727 | -1.376 |
| AI167592 |  |  | 0.0437 | 0.727 | -1.375 |
| NM_005463 | HNRPDL | NM_005463 | 0.0421 | 0.728 | -1.375 |
| AI160440 | USP7 | NM_003470 | 0.0088 | 0.729 | -1.373 |
| AA126793 | HNRNPC | NM_001077442 | 0.0259 | 0.729 | -1.372 |
| BC004862 | UBE2R2 | NM_017811 | 0.0433 | 0.729 | -1.372 |
| AU150752 | ZNF281 | NM_012482 | 0.0117 | 0.730 | -1.370 |
| NM_003746 | DYNLL1 | NM_001037494 | 0.0313 | 0.730 | -1.370 |
| NM_016265 | ZNF12 | NM_006956 | 0.0049 | 0.730 | -1.370 |
| AU144413 | SP3 | NM_001017371 | 0.0326 | 0.730 | -1.370 |
| AA251906 | METT5D1 | NM_152636 | 0.0208 | 0.731 | -1.369 |
| NM_025103 | IFT74 | NM_001099222 | 0.0122 | 0.731 | -1.368 |
| AI707721 |  |  | 0.0175 | 0.731 | -1.368 |
| BF678497 | LIN54 | NM_194282 | 0.0115 | 0.731 | -1.368 |
| AI672159 |  |  | 0.0011 | 0.731 | -1.368 |
| AV700302 | ZNF641 | NM_152320 | 0.0496 | 0.731 | -1.368 |
| AB023215 | TTLL5 | NM_015072 | 0.0406 | 0.731 | -1.367 |
| NM_002398 | MEIS1 | NM_002398 | 0.0214 | 0.731 | -1.367 |
| NM_002533 | NVL | NM_002533 | 0.0142 | 0.732 | -1.366 |
| AB007930 | POGZ | NM_015100 | 0.0368 | 0.732 | -1.366 |
| AL136736 | KIAA1549 | NM_020910 | 0.0475 | 0.732 | -1.366 |
| NM_005077 | TLE1 | NM_005077 | 0.0240 | 0.733 | -1.364 |
| AI088843 | C7orf30 | NM_138446 | 0.0322 | 0.733 | -1.364 |
| T53175 | C7orf38 | NM_145111 | 0.0241 | 0.733 | -1.364 |
| BE856541 | CXorf39 | NM_207318 | 0.0081 | 0.733 | -1.364 |
| AW467472 | APPL1 | NM_012096 | 0.0414 | 0.733 | -1.364 |
| NM_016229 | CYB5R2 | NM_016229 | 0.0460 | 0.733 | -1.364 |
| BC004902 | KIAA0947 | NM_015325 | 0.0468 | 0.734 | -1.363 |
| NM_005012 | ROR1 | NM_001083592 | 0.0375 | 0.734 | -1.363 |
| BC004183 | C10orf119 | NM_024834 | 0.0369 | 0.734 | -1.362 |
| AF179221 | FBXL11 | NM_012308 | 0.0222 | 0.734 | -1.362 |
| BE670307 |  |  | 0.0286 | 0.735 | -1.361 |
| AJ278112 | DEPDC1 | NM_017779 | 0.0138 | 0.735 | -1.360 |
| BF059159 | ROBO1 | NM_002941 | 0.0128 | 0.736 | -1.360 |
| AU116818 | FAM120A | NM_014612 | 0.0134 | 0.736 | -1.359 |
| AB051499 | KIAA1712 | NM_001040157 | 0.0356 | 0.736 | -1.359 |
| AA495988 | C9orf5 | NM_001099734 | 0.0381 | 0.736 | -1.359 |
| AI350995 |  |  | 0.0244 | 0.736 | -1.359 |
| BE620258 |  |  | 0.0436 | 0.736 | -1.359 |
| AB024703 | RNF11 | NM_014372 | 0.0389 | 0.737 | -1.358 |
| NM_018229 | C14orf108 | NM_018229 | 0.0209 | 0.737 | -1.357 |
| AU147399 | CAV1 | NM_001753 | 0.0441 | 0.737 | -1.357 |
| AI936976 | KIAA0562 | NM_014704 | 0.0037 | 0.737 | -1.357 |
| AI765051 | DARS2 | NM_018122 | 0.0320 | 0.737 | -1.357 |
| BF978647 | GFM1 | NM_024996 | 0.0364 | 0.738 | -1.356 |
| U30894 | SGSH | NM_000199 | 0.0299 | 0.739 | -1.354 |
| AI571166 |  |  | 0.0249 | 0.739 | -1.353 |
| AF059318 | USP47 | NM_017944 | 0.0441 | 0.740 | -1.352 |
| AW204564 | CREBZF | NM_001039618 | 0.0259 | 0.740 | -1.352 |
| AV724508 | SDCCAG1 | NM_004713 | 0.0491 | 0.741 | -1.350 |
| NM_003592 | CUL1 | NM_003592 | 0.0196 | 0.741 | -1.349 |
| N38985 | C3orf63 | NM_001112736 | 0.0362 | 0.741 | -1.349 |
| NM_006965 | ZNF24 | NM_006965 | 0.0190 | 0.741 | -1.349 |
| AY137580 | CDC25A | NM_001789 | 0.0170 | 0.742 | -1.348 |
| BF056507 | NSMAF | NM_003580 | 0.0202 | 0.742 | -1.348 |
| AI763123 | ADD3 | NM_001121 | 0.0426 | 0.743 | -1.346 |
| AF131850 | EI24 | NM_001007277 | 0.0191 | 0.743 | -1.346 |
| BF435513 | RASAL2 | NM_004841 | 0.0209 | 0.743 | -1.346 |
| NM_014669 | NUP93 | NM_014669 | 0.0104 | 0.743 | -1.346 |
| AK002054 | COBLL1 | NM_014900 | 0.0265 | 0.744 | -1.344 |
| BF843343 |  |  | 0.0281 | 0.745 | -1.343 |
| AK027184 | BPTF | NM_004459 | 0.0049 | 0.745 | -1.342 |
| AA456955 | ANKRD38 | NM_181712 | 0.0464 | 0.745 | -1.342 |
| NM_019005 | FLJ20323 | NM_019005 | 0.0241 | 0.746 | -1.341 |
| BC038440 | GALNT1 | NM_020474 | 0.0388 | 0.746 | -1.341 |
| NM_004713 | SDCCAG1 | NM_004713 | 0.0136 | 0.746 | -1.340 |
| AK023637 | AMMECR1 | NM_001025580 | 0.0234 | 0.747 | -1.340 |
| NM_002901 | RCN1 | NM_002901 | 0.0029 | 0.747 | -1.339 |
| BC001002 | TUBB | NM_178014 | 0.0345 | 0.747 | -1.339 |
| BE999967 |  |  | 0.0023 | 0.747 | -1.339 |
| BC013009 | ZMYM3 | NM_005096 | 0.0354 | 0.748 | -1.338 |
| BE672408 |  |  | 0.0332 | 0.748 | -1.337 |
| AI339586 | ZNF420 | NM_144689 | 0.0462 | 0.748 | -1.337 |
| NM_006825 | CKAP4 | NM_006825 | 0.0062 | 0.748 | -1.337 |
| NM_017810 | ZNF434 | NM_017810 | 0.0264 | 0.748 | -1.337 |
| NM_021078 | GCN5L2 | NM_021078 | 0.0249 | 0.748 | -1.337 |
| NM_006307 | SRPX | NM_006307 | 0.0207 | 0.748 | -1.337 |
| AI742925 | RAD1 | NM_002853 | 0.0439 | 0.749 | -1.336 |
| BE737620 | PPP1R12A | NM_002480 | 0.0036 | 0.750 | -1.334 |
| AF262027 | RAD23B | NM_002874 | 0.0139 | 0.751 | -1.332 |
| AL561281 | MAP4K4 | NM_004834 | 0.0473 | 0.751 | -1.332 |
| AV707142 | KCTD20 | NM_173562 | 0.0244 | 0.751 | -1.332 |
| AI277617 | FGD4 | NM_139241 | 0.0212 | 0.751 | -1.332 |
| BE858199 | RPL7L1 | NM_198486 | 0.0053 | 0.751 | -1.331 |
| AI803633 | TSR1 | NM_018128 | 0.0159 | 0.751 | -1.331 |
| AI652872 | EPB41L5 | NM_020909 | 0.0068 | 0.752 | -1.330 |
| NM_017681 | NUP62CL | NM_017681 | 0.0239 | 0.752 | -1.329 |
| D84109 | RBPMS | NM_001008710 | 0.0423 | 0.752 | -1.329 |
| BC041481 | FLJ35848 | NM_001033659 | 0.0328 | 0.752 | -1.329 |
| AU157441 | WDR32 | NM_024345 | 0.0303 | 0.753 | -1.328 |
| BF062139 | POLR3G | NM_006467 | 0.0278 | 0.754 | -1.327 |
| AI651265 | CRKRS | NM_015083 | 0.0295 | 0.754 | -1.327 |
| AL514547 | RBM12 | NM_006047 | 0.0198 | 0.754 | -1.327 |
| BQ022804 | LAYN | NM_178834 | 0.0262 | 0.754 | -1.327 |
| AI150690 |  |  | 0.0172 | 0.754 | -1.327 |
| AK025482 | TMEM168 | NM_022484 | 0.0433 | 0.754 | -1.326 |
| AK023184 | KIF1B | NM_015074 | 0.0464 | 0.754 | -1.326 |
| AI744451 |  |  | 0.0264 | 0.755 | -1.325 |
| U49844 | ATR | NM_001184 | 0.0131 | 0.755 | -1.325 |
| AA541758 | CPNE3 | NM_003909 | 0.0442 | 0.755 | -1.324 |
| H48840 | FXR1 | NM_001013438 | 0.0491 | 0.756 | -1.323 |
| AB020712 | SEC31A | NM_001077206 | 0.0277 | 0.756 | -1.322 |
| BE503800 | DDX31 |  | 0.0460 | 0.756 | -1.322 |
| AA973551 |  |  | 0.0071 | 0.757 | -1.321 |
| AL136872 | COMMD4 | NM_017828 | 0.0174 | 0.757 | -1.321 |
| U62325 | APBB2 | NM_173075 | 0.0288 | 0.757 | -1.321 |
| AA448956 | CAMK2D | NM_001221 | 0.0279 | 0.757 | -1.321 |
| NM_004390 | CTSH | NM_004390 | 0.0045 | 0.757 | -1.321 |
| NM_005134 | PPP4R1 | NM_001042388 | 0.0160 | 0.757 | -1.321 |
| W84421 | LOC647121 | NR_003955 | 0.0215 | 0.758 | -1.320 |
| AA479290 |  |  | 0.0355 | 0.758 | -1.319 |
| AB049740 | FUT8 | NM_004480 | 0.0380 | 0.759 | -1.318 |
| AI796010 | RAD1 | NM_002853 | 0.0095 | 0.759 | -1.318 |
| NM_003685 | KHSRP | NM_003685 | 0.0360 | 0.759 | -1.317 |
| NM_002874 | RAD23B | NM_002874 | 0.0080 | 0.759 | -1.317 |
| N24868 | PIAS1 | NM_016166 | 0.0040 | 0.760 | -1.316 |
| BG025078 | FXR1 | NM_001013438 | 0.0377 | 0.760 | -1.316 |
| AF272898 | PRDM6 | XM_927647 | 0.0128 | 0.760 | -1.316 |
| AB018284 | EIF5B | NM_015904 | 0.0124 | 0.761 | -1.314 |
| NM_017735 | TTC27 | NM_017735 | 0.0001 | 0.761 | -1.314 |
| AA026388 |  |  | 0.0128 | 0.761 | -1.314 |
| AI828221 | SHPRH | NM_001042683 | 0.0362 | 0.761 | -1.314 |
| AI340241 | DKFZp686E2433 | XM_293828 | 0.0488 | 0.762 | -1.312 |
| BF966540 | PPP1R2 | NM_006241 | 0.0337 | 0.762 | -1.312 |
| AA594937 | COBL | NM_015198 | 0.0040 | 0.763 | -1.310 |
| NM_018844 | BCAP29 | NM_001008405 | 0.0365 | 0.764 | -1.310 |
| D84109 | RBPMS | NM_001008710 | 0.0053 | 0.764 | -1.309 |
| BG403361 |  |  | 0.0138 | 0.764 | -1.309 |
| AI300168 | ZNF746 | NM_152557 | 0.0386 | 0.764 | -1.309 |
| BG341906 | ARF3 | NM_001659 | 0.0111 | 0.765 | -1.308 |
| AL548941 | KDELC2 | NM_153705 | 0.0123 | 0.765 | -1.307 |
| NM_024615 | PARP8 | NM_024615 | 0.0095 | 0.765 | -1.307 |
| NM_012482 | ZNF281 | NM_012482 | 0.0053 | 0.765 | -1.307 |
| AF268193 | TBL1XR1 | NM_024665 | 0.0255 | 0.766 | -1.306 |
| AV712577 | ANP32B | NM_006401 | 0.0122 | 0.767 | -1.304 |
| BE250417 | ZMYND11 | NM_006624 | 0.0283 | 0.767 | -1.304 |
| AA551784 | CARM1 | NM_199141 | 0.0296 | 0.767 | -1.304 |
| AW026194 | PDCD11 | NM_014976 | 0.0385 | 0.768 | -1.302 |
| AF054589 | MDFIC | NM_199072 | 0.0000 | 0.769 | -1.301 |
| AL136770 | CLDN12 | NM_012129 | 0.0329 | 0.769 | -1.300 |
| NM_006048 | UBE4B | NM_001105562 | 0.0242 | 1.301 | 1.301 |
| AA573502 | TAP2 | NM_000544 | 0.0024 | 1.303 | 1.303 |
| AL527334 |  |  | 0.0442 | 1.303 | 1.303 |
| AV727934 |  |  | 0.0255 | 1.304 | 1.304 |
| AL117612 | MAL2 | NM_052886 | 0.0396 | 1.304 | 1.304 |
| AL080220 | C2CD3 | NM_015531 | 0.0268 | 1.308 | 1.308 |
| NM_001033 | RRM1 | NM_001033 | 0.0392 | 1.310 | 1.310 |
| NM_022067 | C14orf133 | NM_022067 | 0.0408 | 1.311 | 1.311 |
| AU145019 | FRMD4B | NM_015123 | 0.0167 | 1.311 | 1.311 |
| NM_000057 | BLM | NM_000057 | 0.0047 | 1.314 | 1.314 |
| D89678 | HNRPDL | NM_005463 | 0.0280 | 1.314 | 1.314 |
| AW296028 |  |  | 0.0197 | 1.314 | 1.314 |
| AW270158 |  |  | 0.0072 | 1.315 | 1.315 |
| AJ131244 | SEC24A | NM_021982 | 0.0398 | 1.316 | 1.316 |
| NM_018062 | FANCL | NM_018062 | 0.0386 | 1.316 | 1.316 |
| AI587307 | MANEA | NM_024641 | 0.0002 | 1.318 | 1.318 |
| AL573951 | LOC732402 /// PTPLAD1 | NM_016395 | 0.0075 | 1.318 | 1.318 |
| NM_004969 | IDE | NM_004969 | 0.0055 | 1.319 | 1.319 |
| NM_022969 | FGFR2 | NM_000141 | 0.0115 | 1.320 | 1.320 |
| NM_003136 | SRP54 | NM_003136 | 0.0249 | 1.320 | 1.320 |
| BF033242 | CES2 | NM_003869 | 0.0489 | 1.320 | 1.320 |
| NM_005667 | RNF103 | NM_005667 | 0.0174 | 1.321 | 1.321 |
| AF052094 | EPAS1 | NM_001430 | 0.0101 | 1.321 | 1.321 |
| NM_021140 | UTX | NM_021140 | 0.0251 | 1.322 | 1.322 |
| AL047650 | ACBD5 | NM_001042473 | 0.0084 | 1.322 | 1.322 |
| NM_022445 | TPK1 | NM_001042482 | 0.0336 | 1.323 | 1.323 |
| NM_005038 | PPID | NM_005038 | 0.0019 | 1.323 | 1.323 |
| AA206016 |  |  | 0.0406 | 1.325 | 1.325 |
| BE503392 |  |  | 0.0268 | 1.325 | 1.325 |
| BC002447 | PHTF1 | NM_006608 | 0.0281 | 1.326 | 1.326 |
| NM_002945 | RPA1 | NM_002945 | 0.0285 | 1.326 | 1.326 |
| AF112216 | CMPK | NM_016308 | 0.0311 | 1.326 | 1.326 |
| D83485 | PDIA3 | NM_005313 | 0.0338 | 1.326 | 1.326 |
| AI624156 |  |  | 0.0439 | 1.327 | 1.327 |
| AA227879 |  |  | 0.0405 | 1.327 | 1.327 |
| BF055171 | ACOX3 | NM_001101667 | 0.0454 | 1.329 | 1.329 |
| BG054844 | RND3 | NM_005168 | 0.0012 | 1.331 | 1.331 |
| AK055438 |  |  | 0.0363 | 1.332 | 1.332 |
| BG548811 | ZRANB3 | NM_032143 | 0.0133 | 1.333 | 1.333 |
| NM_000161 | GCH1 | NM_000161 | 0.0206 | 1.333 | 1.333 |
| AI478300 | NFATC2IP | NM_032815 | 0.0173 | 1.334 | 1.334 |
| NM_018318 | CCDC91 | NM_018318 | 0.0425 | 1.334 | 1.334 |
| AV734793 | ZDBF2 | NM_020923 | 0.0192 | 1.336 | 1.336 |
| AI889160 | CABLES1 | NM_001100619 | 0.0314 | 1.337 | 1.337 |
| M87771 | FGFR2 | NM_000141 | 0.0310 | 1.338 | 1.338 |
| AA886888 |  |  | 0.0376 | 1.338 | 1.338 |
| AF210057 | C3orf1 | NM_016589 | 0.0272 | 1.338 | 1.338 |
| BG501219 | TMEM167 | NM_174909 | 0.0331 | 1.338 | 1.338 |
| AA779684 | BRMS1L | NM_032352 | 0.0122 | 1.339 | 1.339 |
| NM_022735 | ACBD3 | NM_022735 | 0.0229 | 1.340 | 1.340 |
| NM_002013 | FKBP3 | NM_002013 | 0.0290 | 1.341 | 1.341 |
| AK025872 | TNRC8 |  | 0.0498 | 1.341 | 1.341 |
| AI990326 | MPHOSPH9 | NM_022782 | 0.0230 | 1.344 | 1.344 |
| AA634272 | STAT3 | NM_003150 | 0.0228 | 1.345 | 1.345 |
| X14174 | ALPL | NM_000478 | 0.0098 | 1.345 | 1.345 |
| W93554 | SH3PXD2A | NM_014631 | 0.0347 | 1.346 | 1.346 |
| AA129773 | MAPK1 | NM_002745 | 0.0063 | 1.348 | 1.348 |
| NM_003330 | TXNRD1 | NM_001093771 | 0.0012 | 1.348 | 1.348 |
| X57348 | SFN | NM_006142 | 0.0375 | 1.348 | 1.348 |
| BF223370 |  |  | 0.0357 | 1.349 | 1.349 |
| BF516305 |  |  | 0.0404 | 1.350 | 1.350 |
| BC036200 | C1orf71 | NM_152609 | 0.0171 | 1.350 | 1.350 |
| AA843238 | SLU7 | NM_006425 | 0.0043 | 1.350 | 1.350 |
| AF072098 | TPT1 | NM_003295 | 0.0215 | 1.351 | 1.351 |
| NM_015542 | UPF2 | NM_015542 | 0.0411 | 1.352 | 1.352 |
| AL110136 | LOC440944 | XR_017845 | 0.0285 | 1.352 | 1.352 |
| NM_014711 | CP110 | NM_014711 | 0.0047 | 1.352 | 1.352 |
| AL133267 | LOC442175 | XM_001130492 | 0.0242 | 1.353 | 1.353 |
| NM_015986 | CRLF3 | NM_015986 | 0.0405 | 1.353 | 1.353 |
| NM_003133 | SRP9 | NM_003133 | 0.0063 | 1.353 | 1.353 |
| AI927993 | OSBP | NM_002556 | 0.0117 | 1.355 | 1.355 |
| NM_018023 | YEATS2 | NM_018023 | 0.0064 | 1.356 | 1.356 |
| BF114745 |  |  | 0.0437 | 1.356 | 1.356 |
| BG109855 | SEMA5A | NM_003966 | 0.0322 | 1.356 | 1.356 |
| BC001393 | C2orf24 | NM_015680 | 0.0271 | 1.358 | 1.358 |
| H40020 |  |  | 0.0395 | 1.360 | 1.360 |
| AI991996 | KIAA1211 | NM_020722 | 0.0403 | 1.360 | 1.360 |
| AU145642 | C16orf52 | NM_173501 | 0.0468 | 1.361 | 1.361 |
| AL031295 | hCG_2003956 /// LYPLA2 /// LYPLA2P1 | NM_007260 | 0.0395 | 1.362 | 1.362 |
| NM_016399 | TRIAP1 | NM_016399 | 0.0480 | 1.362 | 1.362 |
| NM_006353 | HMGN4 | NM_006353 | 0.0060 | 1.364 | 1.364 |
| AW006290 | RIOK3 | NM_003831 | 0.0014 | 1.364 | 1.364 |
| BE677844 |  |  | 0.0283 | 1.366 | 1.366 |
| AA975427 |  |  | 0.0384 | 1.367 | 1.367 |
| H09470 | FLJ31958 |  | 0.0368 | 1.367 | 1.367 |
| BC005176 | TM7SF3 | NM_016551 | 0.0075 | 1.368 | 1.368 |
| NM_025201 | PLEKHO2 | NM_025201 | 0.0089 | 1.368 | 1.368 |
| BG104860 | CSNK1G1 | NM_022048 | 0.0252 | 1.368 | 1.368 |
| BG496998 | FAM33A | NM_001100595 | 0.0138 | 1.369 | 1.369 |
| AI949179 | BCL2L11 | NM_006538 | 0.0113 | 1.370 | 1.370 |
| NM_017998 | C9orf40 | NM_017998 | 0.0040 | 1.370 | 1.370 |
| NM_030801 | MAGED4 /// MAGED4B | NM_001098800 | 0.0373 | 1.371 | 1.371 |
| NM_006810 | PDIA5 | NM_006810 | 0.0453 | 1.371 | 1.371 |
| NM_002318 | LOXL2 | NM_002318 | 0.0278 | 1.372 | 1.372 |
| AI378406 | EGLN3 | NM_022073 | 0.0097 | 1.372 | 1.372 |
| AW188940 | B2M | NM_004048 | 0.0314 | 1.374 | 1.374 |
| BF666293 | FVT1 | NM_002035 | 0.0106 | 1.374 | 1.374 |
| BC001362 | CNP | NM_033133 | 0.0205 | 1.375 | 1.375 |
| NM_021947 | SRR | NM_021947 | 0.0395 | 1.375 | 1.375 |
| NM_005475 | SH2B3 | NM_005475 | 0.0243 | 1.376 | 1.376 |
| AL542358 | SLC36A4 | NM_152313 | 0.0134 | 1.377 | 1.377 |
| AI674647 | SPPL2A | NM_032802 | 0.0300 | 1.377 | 1.377 |
| BE890365 | WWC2 | NM_024949 | 0.0284 | 1.378 | 1.378 |
| AI190287 | ZNF788 | XR_015208 | 0.0477 | 1.380 | 1.380 |
| AK057473 | LOC339260 |  | 0.0263 | 1.382 | 1.382 |
| U62317 | LMF2 | NM_033200 | 0.0401 | 1.382 | 1.382 |
| NM_014736 | KIAA0101 | NM_001029989 | 0.0035 | 1.383 | 1.383 |
| AB007899 | NEDD4L | NM_015277 | 0.0310 | 1.384 | 1.384 |
| NM_022471 | GMCL1 | NM_178439 | 0.0426 | 1.384 | 1.384 |
| AI807211 |  |  | 0.0043 | 1.385 | 1.385 |
| NM_018639 | WSB2 | NM_018639 | 0.0146 | 1.385 | 1.385 |
| BF540749 |  |  | 0.0300 | 1.386 | 1.386 |
| BG391282 |  |  | 0.0257 | 1.387 | 1.387 |
| N51717 |  |  | 0.0329 | 1.387 | 1.387 |
| NM_015710 | GLTSCR2 | NM_015710 | 0.0036 | 1.388 | 1.388 |
| NM_013257 | SGK3 | NM_001033578 | 0.0482 | 1.388 | 1.388 |
| BE897866 | ACADSB | NM_001609 | 0.0033 | 1.388 | 1.388 |
| AI354864 | GPC1 | NM_002081 | 0.0299 | 1.388 | 1.388 |
| BG537190 | FTL | NM_000146 | 0.0001 | 1.393 | 1.393 |
| BC005997 | C1orf97 | NM_032705 | 0.0280 | 1.393 | 1.393 |
| NM_003610 | RAE1 | NM_001015885 | 0.0025 | 1.393 | 1.393 |
| AI701170 |  |  | 0.0198 | 1.395 | 1.395 |
| BE645144 | FAM73A | NM_198549 | 0.0181 | 1.395 | 1.395 |
| AV758242 | CCDC111 | NM_152683 | 0.0400 | 1.395 | 1.395 |
| AI816243 | STX12 | NM_177424 | 0.0129 | 1.396 | 1.396 |
| NM_016371 | HSD17B7 /// HSD17B7P2 /// LOC730412 | NM_016371 | 0.0379 | 1.396 | 1.396 |
| AI749451 | CISD2 | NM_001008388 | 0.0382 | 1.396 | 1.396 |
| AI831738 | DDX59 | NM_001031725 | 0.0232 | 1.396 | 1.396 |
| BC000873 | GNB4 | NM_021629 | 0.0448 | 1.396 | 1.396 |
| AL519710 | CADM1 | NM_001098517 | 0.0100 | 1.396 | 1.396 |
| AB020681 | ANKRD12 | NM_001083625 | 0.0212 | 1.397 | 1.397 |
| AF072506 | ERVWE1 | NM_014590 | 0.0172 | 1.400 | 1.400 |
| AI032786 | EDG5 | NM_004230 | 0.0075 | 1.401 | 1.401 |
| BU846215 |  |  | 0.0242 | 1.401 | 1.401 |
| NM_012382 | TTC33 | NM_012382 | 0.0031 | 1.402 | 1.402 |
| AI754404 | PLOD2 | NM_000935 | 0.0097 | 1.402 | 1.402 |
| AI744083 | MOSPD2 | NM_152581 | 0.0438 | 1.402 | 1.402 |
| AI760772 | RFFL | NM_001017368 | 0.0233 | 1.403 | 1.403 |
| NM_019114 | EPB41L4B | NM_018424 | 0.0082 | 1.405 | 1.405 |
| D85181 | SC5DL | NM_001024956 | 0.0306 | 1.407 | 1.407 |
| R43205 | GUSBL2 | NM_206908 | 0.0250 | 1.408 | 1.408 |
| BC000268 | PSMB2 | NM_002794 | 0.0029 | 1.408 | 1.408 |
| AL513583 | GM2A | NM_000405 | 0.0359 | 1.408 | 1.408 |
| NM_013229 | APAF1 | NM_001160 | 0.0407 | 1.409 | 1.409 |
| AV683529 | C2orf49 |  | 0.0349 | 1.411 | 1.411 |
| BF185904 | GRPEL2 | NM_152407 | 0.0005 | 1.411 | 1.411 |
| AI627666 | FCHO2 | NM_138782 | 0.0242 | 1.411 | 1.411 |
| BC011119 | SPIRE2 | NM_032451 | 0.0136 | 1.412 | 1.412 |
| AW504458 | GNB4 | NM_021629 | 0.0433 | 1.412 | 1.412 |
| N51405 | DXS542 |  | 0.0217 | 1.413 | 1.413 |
| BC004185 | C16orf35 | NM_001039476 | 0.0145 | 1.413 | 1.413 |
| NM_023948 | MOSPD3 | NM_001040097 | 0.0116 | 1.413 | 1.413 |
| NM_024310 | PLEKHF1 | NM_024310 | 0.0226 | 1.415 | 1.415 |
| BG284827 |  |  | 0.0420 | 1.415 | 1.415 |
| AL136827 | WDR37 | NM_014023 | 0.0216 | 1.417 | 1.417 |
| AB037793 | USP35 | NM_020798 | 0.0255 | 1.417 | 1.417 |
| AV704232 |  |  | 0.0090 | 1.418 | 1.418 |
| N51263 | PHCA | NM_018367 | 0.0385 | 1.419 | 1.419 |
| AB033058 | DLG3 | NM_020730 | 0.0326 | 1.420 | 1.420 |
| BC039551 |  |  | 0.0473 | 1.422 | 1.422 |
| AI307586 |  |  | 0.0177 | 1.423 | 1.423 |
| D83243 | NPAT | NM_002519 | 0.0123 | 1.423 | 1.423 |
| M31659 | SLC25A16 | NM_152707 | 0.0319 | 1.428 | 1.428 |
| AI355709 | ZNF789 | NM_001013258 | 0.0070 | 1.428 | 1.428 |
| AV702692 |  |  | 0.0113 | 1.429 | 1.429 |
| AF158185 | POLH | NM_006502 | 0.0256 | 1.429 | 1.429 |
| BF038366 | TMEM97 | NM_014573 | 0.0137 | 1.429 | 1.429 |
| NM_152327 | AK7 | NM_152327 | 0.0362 | 1.430 | 1.430 |
| AK001393 | EFCAB2 | NM_032328 | 0.0460 | 1.431 | 1.431 |
| BF219240 | ZNF655 | NM_001009956 | 0.0147 | 1.432 | 1.432 |
| NM_001755 | CBFB | NM_001755 | 0.0022 | 1.433 | 1.433 |
| BE256900 | JMJD2B | NM_015015 | 0.0320 | 1.435 | 1.435 |
| NM_001294 | CLPTM1 | NM_001294 | 0.0214 | 1.435 | 1.435 |
| AW150236 | SNX16 | NM_022133 | 0.0151 | 1.437 | 1.437 |
| BG035985 | HMGCS1 | NM_001098272 | 0.0166 | 1.438 | 1.438 |
| AI341146 | E2F7 | NM_203394 | 0.0066 | 1.439 | 1.439 |
| NM_003422 | MZF1 | NM_003422 | 0.0319 | 1.439 | 1.439 |
| AW237290 |  |  | 0.0032 | 1.440 | 1.440 |
| BC000143 | ELMO2 | NM_133171 | 0.0342 | 1.440 | 1.440 |
| NM_004855 | PIGB | NM_004855 | 0.0397 | 1.441 | 1.441 |
| NM_023923 | PHACTR4 | NM_001048183 | 0.0026 | 1.441 | 1.441 |
| NM_000617 | SLC11A2 | NM_000617 | 0.0422 | 1.441 | 1.441 |
| AA453163 | PCMTD1 | NM_052937 | 0.0040 | 1.442 | 1.442 |
| BF979497 | SQLE | NM_003129 | 0.0485 | 1.442 | 1.442 |
| NM_006564 | CXCR6 | NM_006564 | 0.0344 | 1.443 | 1.443 |
| NM_024899 | CEP76 | NM_024899 | 0.0243 | 1.445 | 1.445 |
| AI659800 | C13orf31 | NM_153218 | 0.0127 | 1.445 | 1.445 |
| NM_005561 | LAMP1 | NM_005561 | 0.0024 | 1.446 | 1.446 |
| AA653638 |  |  | 0.0404 | 1.446 | 1.446 |
| BG393032 | LOC641845 /// LOC647087 /// SLC13A4 | NM_012450 | 0.0075 | 1.446 | 1.446 |
| AL049452 | LOC144874 |  | 0.0485 | 1.447 | 1.447 |
| AV690866 | SGK3 | NM_001033578 | 0.0203 | 1.448 | 1.448 |
| AW291187 | C1orf71 | NM_152609 | 0.0151 | 1.448 | 1.448 |
| AW611729 | CEP27 | NM_018097 | 0.0071 | 1.448 | 1.448 |
| NM_006493 | CLN5 | NM_006493 | 0.0309 | 1.449 | 1.449 |
| AI148567 | USP32 | NM_032582 | 0.0186 | 1.449 | 1.449 |
| AL534095 | GPR177 | NM_001002292 | 0.0309 | 1.451 | 1.451 |
| AI796222 |  |  | 0.0476 | 1.457 | 1.457 |
| BF223300 | ENAH | NM_001008493 | 0.0115 | 1.457 | 1.457 |
| BC004162 | PPARA | NM_001001928 | 0.0384 | 1.457 | 1.457 |
| AL534095 | GPR177 | NM_001002292 | 0.0303 | 1.458 | 1.458 |
| AL569476 | ANKRD13A | NM_033121 | 0.0369 | 1.458 | 1.458 |
| AK023732 | RBM41 | NM_018301 | 0.0311 | 1.459 | 1.459 |
| NM_018986 | SH3TC1 | NM_018986 | 0.0459 | 1.460 | 1.460 |
| AI890529 |  |  | 0.0484 | 1.461 | 1.461 |
| NM_024854 | PYROXD1 | NM_024854 | 0.0036 | 1.461 | 1.461 |
| Y16521 | CDS2 | NM_003818 | 0.0360 | 1.462 | 1.462 |
| AL037450 | RIT1 | NM_006912 | 0.0328 | 1.463 | 1.463 |
| AI539710 | ABCC1 | NM_004996 | 0.0068 | 1.463 | 1.463 |
| BC005127 | ADFP | NM_001122 | 0.0116 | 1.464 | 1.464 |
| AA514384 | PHPT1 | NM_014172 | 0.0295 | 1.465 | 1.465 |
| AB050049 | MCCC2 | NM_022132 | 0.0198 | 1.466 | 1.466 |
| AF272036 | RRAGD | NM_021244 | 0.0220 | 1.467 | 1.467 |
| NM_031296 | RAB33B | NM_031296 | 0.0277 | 1.469 | 1.469 |
| AI675308 |  |  | 0.0138 | 1.469 | 1.469 |
| AA574240 | LOC90826 | NM_138364 | 0.0065 | 1.470 | 1.470 |
| BG285017 | HDGFRP3 | NM_016073 | 0.0049 | 1.471 | 1.471 |
| AB032261 | SCD | NM_005063 | 0.0003 | 1.471 | 1.471 |
| N38751 | KLHL22 | NM_032775 | 0.0230 | 1.471 | 1.471 |
| AW084510 | LSS | NM_001001438 | 0.0303 | 1.472 | 1.472 |
| AF225425 | SEMA6A | NM_020796 | 0.0008 | 1.472 | 1.472 |
| AW444944 |  |  | 0.0465 | 1.473 | 1.473 |
| AI761250 | MBOAT2 | NM_138799 | 0.0383 | 1.473 | 1.473 |
| AK098125 | RETSAT | NM_017750 | 0.0428 | 1.473 | 1.473 |
| AI246590 | IRAK2 | NM_001570 | 0.0249 | 1.474 | 1.474 |
| NM_021183 | RAP2C | NM_021183 | 0.0213 | 1.475 | 1.475 |
| AI140985 |  |  | 0.0127 | 1.475 | 1.475 |
| NM_021729 | VPS11 | NM_021729 | 0.0460 | 1.476 | 1.476 |
| NM_014959 | CARD8 | NM_014959 | 0.0032 | 1.477 | 1.477 |
| NM_004688 | NMI | NM_004688 | 0.0430 | 1.477 | 1.477 |
| AF021834 | TFPI | NM_001032281 | 0.0292 | 1.477 | 1.477 |
| BC005979 | UBE2B | NM_003337 | 0.0261 | 1.477 | 1.477 |
| X57348 | SFN | NM_006142 | 0.0012 | 1.480 | 1.480 |
| AA743462 |  |  | 0.0114 | 1.480 | 1.480 |
| NM_016325 | ZNF274 | NM_016324 | 0.0353 | 1.481 | 1.481 |
| NM_001673 | ASNS | NM_001673 | 0.0238 | 1.482 | 1.482 |
| AB028951 | CDC2L6 | NM_015076 | 0.0399 | 1.485 | 1.485 |
| NM_017911 | FAM118A | NM_001104595 | 0.0013 | 1.485 | 1.485 |
| BE645154 |  |  | 0.0435 | 1.486 | 1.486 |
| AI684281 | P15RS | NM_018170 | 0.0148 | 1.487 | 1.487 |
| AI910842 |  |  | 0.0415 | 1.487 | 1.487 |
| AA219354 | HPS3 | NM_032383 | 0.0153 | 1.488 | 1.488 |
| BE962615 | SNX3 | NM_003795 | 0.0382 | 1.489 | 1.489 |
| BC004419 | VPS24 | NM_001005753 | 0.0246 | 1.489 | 1.489 |
| AI339606 | C10orf88 | NM_024942 | 0.0011 | 1.489 | 1.489 |
| BC001282 | HMGN4 | NM_006353 | 0.0101 | 1.490 | 1.490 |
| NM_080867 | SOCS4 | NM_080867 | 0.0093 | 1.492 | 1.492 |
| NM_014905 | GLS | NM_014905 | 0.0092 | 1.492 | 1.492 |
| AW575737 | CCDC32 | NM_001080791 | 0.0477 | 1.493 | 1.493 |
| AL049942 | ZNF337 | NM_015655 | 0.0331 | 1.494 | 1.494 |
| NM_003692 | TMEFF1 | NM_003692 | 0.0303 | 1.494 | 1.494 |
| AI635131 | C1orf136 |  | 0.0087 | 1.495 | 1.495 |
| AW276572 | SBF2 | NM_030962 | 0.0038 | 1.495 | 1.495 |
| AF126181 | MAGED2 | NM_014599 | 0.0172 | 1.497 | 1.497 |
| AF088033 | VCPIP1 |  | 0.0090 | 1.498 | 1.498 |
| BF000047 |  |  | 0.0234 | 1.498 | 1.498 |
| BC000282 | TMEM116 | NM_138341 | 0.0032 | 1.498 | 1.498 |
| NM_016061 | YPEL5 | NM_016061 | 0.0123 | 1.499 | 1.499 |
| NM_024942 | C10orf88 | NM_024942 | 0.0311 | 1.500 | 1.500 |
| AA460299 | MLF1IP | NM_024629 | 0.0287 | 1.500 | 1.500 |
| NM_007034 | DNAJB4 | NM_007034 | 0.0230 | 1.501 | 1.501 |
| AL564683 | CEBPB | NM_005194 | 0.0388 | 1.502 | 1.502 |
| NM_002032 | FTH1 | NM_002032 | 0.0157 | 1.505 | 1.505 |
| AI339732 | CIAO1 | NM_004804 | 0.0364 | 1.506 | 1.506 |
| AI766279 |  |  | 0.0145 | 1.506 | 1.506 |
| BF115203 | MPP5 | NM_022474 | 0.0123 | 1.506 | 1.506 |
| NM_000259 | MYO5A | NM_000259 | 0.0120 | 1.507 | 1.507 |
| AA777752 | ELOVL6 |  | 0.0049 | 1.511 | 1.511 |
| AW993257 |  |  | 0.0210 | 1.512 | 1.512 |
| AI742358 | SVIP | NM_148893 | 0.0095 | 1.513 | 1.513 |
| AW136032 |  |  | 0.0160 | 1.513 | 1.513 |
| BG028765 | LIN52 | NM_001024674 | 0.0197 | 1.515 | 1.515 |
| AB033024 | ZNF490 | NM_020714 | 0.0322 | 1.518 | 1.518 |
| J04755 | FTHP1 |  | 0.0034 | 1.519 | 1.519 |
| AF016266 | TNFRSF10B | NM_003842 | 0.0069 | 1.519 | 1.519 |
| AL122088 | LYSMD1 | NM_212551 | 0.0462 | 1.519 | 1.519 |
| AK092760 | ZNF564 | NM_144976 | 0.0085 | 1.520 | 1.520 |
| NM_024498 | ZNF117 | NM_015852 | 0.0305 | 1.522 | 1.522 |
| AF288392 | C1orf26 | NM_001105518 | 0.0397 | 1.522 | 1.522 |
| NM_002946 | RPA2 | NM_002946 | 0.0438 | 1.525 | 1.525 |
| AI768723 | UBE2B | NM_003337 | 0.0091 | 1.526 | 1.526 |
| AI800025 |  |  | 0.0102 | 1.526 | 1.526 |
| AA648506 | FAM149B1 |  | 0.0055 | 1.527 | 1.527 |
| AW299507 | GGPS1 | NM_001037277 | 0.0188 | 1.528 | 1.528 |
| AI458208 |  |  | 0.0228 | 1.530 | 1.530 |
| AW592266 | MYBL1 | NM_001080416 | 0.0076 | 1.532 | 1.532 |
| NM_004294 | MTRF1 | NM_004294 | 0.0098 | 1.533 | 1.533 |
| NM_014665 | LRRC14 | NM_014665 | 0.0448 | 1.534 | 1.534 |
| NM_018456 | EAF2 | NM_018456 | 0.0211 | 1.534 | 1.534 |
| BM980001 | APOL6 | NM_030641 | 0.0447 | 1.534 | 1.534 |
| AI625741 | UBE2W | NM_001001481 | 0.0382 | 1.535 | 1.535 |
| BC043596 | FANCB | NM_001018113 | 0.0081 | 1.536 | 1.536 |
| AW131553 | C21orf86 | NM_153454 | 0.0340 | 1.536 | 1.536 |
| BE674103 | CROT | NM_021151 | 0.0290 | 1.536 | 1.536 |
| AW612407 | PHF20L1 | NM_016018 | 0.0014 | 1.538 | 1.538 |
| NM_001935 | DPP4 | NM_001935 | 0.0203 | 1.538 | 1.538 |
| AF098865 | SQLE | NM_003129 | 0.0173 | 1.540 | 1.540 |
| BE857704 |  |  | 0.0485 | 1.540 | 1.540 |
| AF070448 | CTSL2 | NM_001333 | 0.0348 | 1.541 | 1.541 |
| AI090331 | PPP1R7 |  | 0.0168 | 1.542 | 1.542 |
| BF593252 | ADSSL1 | NM_152328 | 0.0369 | 1.542 | 1.542 |
| AW295547 | WIPF2 | NM_133264 | 0.0227 | 1.542 | 1.542 |
| AV734646 | FAM26F | NM_001010919 | 0.0409 | 1.543 | 1.543 |
| AF111804 | CAMTA1 | NM_015215 | 0.0143 | 1.544 | 1.544 |
| BE856302 |  |  | 0.0429 | 1.546 | 1.546 |
| AA553722 | SPIRE2 | NM_032451 | 0.0061 | 1.547 | 1.547 |
| NM_004260 | RECQL4 | NM_004260 | 0.0436 | 1.549 | 1.549 |
| NM_003563 | SPOP | NM_001007226 | 0.0430 | 1.550 | 1.550 |
| NM_024610 | HSPBAP1 | NM_024610 | 0.0160 | 1.554 | 1.554 |
| AW024656 |  |  | 0.0321 | 1.556 | 1.556 |
| BE620598 | LOC201725 | NM_001008393 | 0.0057 | 1.557 | 1.557 |
| AB051515 | TANC1 | NM_033394 | 0.0278 | 1.559 | 1.559 |
| AA744682 | LOC653256 /// RABL3 | NM_173825 | 0.0077 | 1.561 | 1.561 |
| NM_005044 | PRKX | NM_005044 | 0.0465 | 1.562 | 1.562 |
| NM_004431 | EPHA2 | NM_004431 | 0.0081 | 1.564 | 1.564 |
| AI765445 | BTG3 | NM_006806 | 0.0331 | 1.564 | 1.564 |
| AW070229 | IQCK | NM_153208 | 0.0043 | 1.566 | 1.566 |
| NM_005213 | CSTA | NM_005213 | 0.0453 | 1.567 | 1.567 |
| NM_000235 | LIPA | NM_000235 | 0.0431 | 1.568 | 1.568 |
| AL577866 | ZNF615 | NM_198480 | 0.0341 | 1.569 | 1.569 |
| NM_003408 | ZFP37 | NM_003408 | 0.0209 | 1.570 | 1.570 |
| NM_014872 | ZBTB5 | NM_014872 | 0.0180 | 1.570 | 1.570 |
| AL365375 | SIRT6 | NM_016539 | 0.0487 | 1.572 | 1.572 |
| AA460299 | MLF1IP | NM_024629 | 0.0401 | 1.572 | 1.572 |
| NM_000950 | PRRG1 | NM_000950 | 0.0123 | 1.574 | 1.574 |
| NM_018656 | SLC35E3 | NM_018656 | 0.0262 | 1.575 | 1.575 |
| AL133609 | CCDC21 | NM_022778 | 0.0262 | 1.575 | 1.575 |
| AW162758 |  |  | 0.0261 | 1.575 | 1.575 |
| BC038383 | TMEM80 | NM_001042463 | 0.0348 | 1.579 | 1.579 |
| AI760332 |  |  | 0.0361 | 1.581 | 1.581 |
| AK026921 | SLC17A5 | NM_012434 | 0.0206 | 1.583 | 1.583 |
| BE217882 | JHDM1D | NM_030647 | 0.0241 | 1.583 | 1.583 |
| AK056852 | LOC144571 |  | 0.0027 | 1.584 | 1.584 |
| NM_000935 | PLOD2 | NM_000935 | 0.0035 | 1.584 | 1.584 |
| BF131886 | SESN2 | NM_031459 | 0.0070 | 1.584 | 1.584 |
| AW449169 | SPOP | NM_001007226 | 0.0437 | 1.585 | 1.585 |
| AF022375 | VEGFA | NM_001025366 | 0.0087 | 1.587 | 1.587 |
| AB041261 | PNPLA8 | NM_015723 | 0.0215 | 1.588 | 1.588 |
| AA417878 | RIT1 | NM_006912 | 0.0175 | 1.589 | 1.589 |
| AW188087 | FLJ30428 /// LOC730024 | XM_496597 | 0.0494 | 1.589 | 1.589 |
| NM_014399 | TSPAN13 | NM_014399 | 0.0153 | 1.591 | 1.591 |
| AI761561 | HK2 | NM_000189 | 0.0189 | 1.591 | 1.591 |
| X16354 | CEACAM1 | NM_001024912 | 0.0232 | 1.592 | 1.592 |
| NM_024810 | CXorf45 | NM_001039210 | 0.0456 | 1.592 | 1.592 |
| AI433712 | MUT | NM_000255 | 0.0240 | 1.595 | 1.595 |
| AF217519 | PNPLA8 | NM_015723 | 0.0320 | 1.597 | 1.597 |
| NM_022168 | IFIH1 | NM_022168 | 0.0305 | 1.597 | 1.597 |
| NM_016508 | CDKL3 | NM_016508 | 0.0182 | 1.599 | 1.599 |
| NM_004779 | CNOT8 | NM_004779 | 0.0372 | 1.600 | 1.600 |
| AI633652 |  |  | 0.0475 | 1.600 | 1.600 |
| AI962276 | PCMTD1 | NM_052937 | 0.0245 | 1.600 | 1.600 |
| AA488687 | SLC7A11 | NM_014331 | 0.0299 | 1.601 | 1.601 |
| NM_001107 | ACYP1 | NM_001107 | 0.0304 | 1.602 | 1.602 |
| NM_014314 | DDX58 | NM_014314 | 0.0034 | 1.602 | 1.602 |
| NM_021249 | SNX6 | NM_021249 | 0.0374 | 1.603 | 1.603 |
| AI934828 |  |  | 0.0484 | 1.603 | 1.603 |
| AI819043 | CREB5 | NM_001011666 | 0.0475 | 1.604 | 1.604 |
| AW263542 |  |  | 0.0078 | 1.614 | 1.614 |
| AV734646 | FAM26F | NM_001010919 | 0.0212 | 1.616 | 1.616 |
| AW189097 |  |  | 0.0073 | 1.616 | 1.616 |
| AI335509 |  |  | 0.0439 | 1.621 | 1.621 |
| AF059274 | CSPG5 | NM_006574 | 0.0361 | 1.622 | 1.622 |
| U80737 | NCOA3 | NM_006534 | 0.0228 | 1.622 | 1.622 |
| BG432350 | C20orf108 | NM_080821 | 0.0016 | 1.622 | 1.622 |
| AA166617 | WDR37 | NM_014023 | 0.0202 | 1.623 | 1.623 |
| AA992936 |  |  | 0.0089 | 1.624 | 1.624 |
| NM_003447 | ZNF165 | NM_003447 | 0.0240 | 1.626 | 1.626 |
| BC005832 | KIAA0101 | NM_001029989 | 0.0059 | 1.626 | 1.626 |
| BC004973 | STAT6 | NM_003153 | 0.0232 | 1.627 | 1.627 |
| AA812232 | TXNIP | NM_006472 | 0.0305 | 1.627 | 1.627 |
| NM_032763 | MGC16142 |  | 0.0149 | 1.627 | 1.627 |
| AL121936 | BTN2A1 | NM_007049 | 0.0500 | 1.627 | 1.627 |
| AI928037 | RUNDC3B | NM_138290 | 0.0134 | 1.630 | 1.630 |
| BE562742 |  |  | 0.0027 | 1.631 | 1.631 |
| N48315 | PPARA | NM_001001928 | 0.0029 | 1.631 | 1.631 |
| AB040883 | KIAA1450 | NM_020840 | 0.0027 | 1.631 | 1.631 |
| NM_022840 | METTL4 | NM_022840 | 0.0150 | 1.632 | 1.632 |
| AI263909 | RHOB | NM_004040 | 0.0401 | 1.633 | 1.633 |
| AL080081 | DNAJB9 | NM_012328 | 0.0060 | 1.633 | 1.633 |
| AL031714 | UBE2I | NM_003345 | 0.0257 | 1.633 | 1.633 |
| AB047006 | PCGF6 | NM_001011663 | 0.0161 | 1.637 | 1.637 |
| NM_016217 | HECA | NM_016217 | 0.0380 | 1.638 | 1.638 |
| AW242220 | EIF4E2 | NM_004846 | 0.0370 | 1.639 | 1.639 |
| AW515645 | FRMD4A | NM_018027 | 0.0088 | 1.639 | 1.639 |
| NM_018665 | DDX43 | NM_018665 | 0.0413 | 1.640 | 1.640 |
| NM_017917 | PPP2R3C | NM_017917 | 0.0091 | 1.643 | 1.643 |
| D80480 | TMTC4 | NM_001079669 | 0.0139 | 1.645 | 1.645 |
| AI979261 | LOC202451 | XM_928403 | 0.0069 | 1.645 | 1.645 |
| AL136944 | SLC40A1 | NM_014585 | 0.0288 | 1.647 | 1.647 |
| NM_018336 |  |  | 0.0128 | 1.648 | 1.648 |
| BE439987 | GAS7 | NM_003644 | 0.0239 | 1.649 | 1.649 |
| AA160474 | C20orf111 | NM_016470 | 0.0140 | 1.650 | 1.650 |
| NM_014278 | HSPA4L | NM_014278 | 0.0364 | 1.650 | 1.650 |
| BC001188 | TFRC | NM_003234 | 0.0041 | 1.653 | 1.653 |
| AF115515 | C3orf33 | NM_173657 | 0.0344 | 1.654 | 1.654 |
| N51479 | ATXN3 | NM_001024631 | 0.0340 | 1.655 | 1.655 |
| AB051511 | SELI | NM_033505 | 0.0058 | 1.656 | 1.656 |
| AU144102 | SNRPE | NM_003094 | 0.0337 | 1.657 | 1.657 |
| AB037741 | HACE1 | NM_020771 | 0.0013 | 1.658 | 1.658 |
| AI335267 |  |  | 0.0216 | 1.658 | 1.658 |
| AW511135 | NUDT4 | NM_019094 | 0.0245 | 1.658 | 1.658 |
| BE880245 | GNS | NM_002076 | 0.0286 | 1.659 | 1.659 |
| AF060922 | BNIP3L | NM_004331 | 0.0240 | 1.660 | 1.660 |
| AI652845 | LRRC51 | NM_145309 | 0.0389 | 1.667 | 1.667 |
| BE855799 | KIAA1211 | NM_020722 | 0.0292 | 1.668 | 1.668 |
| AI978623 | OBSL1 | NM_015311 | 0.0433 | 1.669 | 1.669 |
| AI797063 | KIAA1377 | NM_020802 | 0.0051 | 1.671 | 1.671 |
| M80536 | DPP4 | NM_001935 | 0.0138 | 1.674 | 1.674 |
| BF970855 | MED12L | NM_053002 | 0.0389 | 1.676 | 1.676 |
| AA764787 | METTL4 | NM_022840 | 0.0197 | 1.676 | 1.676 |
| AV727336 | LOC401152 | NM_001001701 | 0.0296 | 1.678 | 1.678 |
| AF126163 | HHLA3 | NM_001031693 | 0.0068 | 1.678 | 1.678 |
| BC000586 | SCLY | NM_016510 | 0.0336 | 1.678 | 1.678 |
| NM_014762 | DHCR24 | NM_014762 | 0.0345 | 1.679 | 1.679 |
| AW080025 |  |  | 0.0221 | 1.679 | 1.679 |
| AU146105 | ATXN3 | NM_001024631 | 0.0234 | 1.679 | 1.679 |
| AA573449 | MTRF1 | NM_004294 | 0.0230 | 1.682 | 1.682 |
| BG028320 |  |  | 0.0019 | 1.682 | 1.682 |
| D83768 | UBXD6 | NM_005671 | 0.0419 | 1.682 | 1.682 |
| AV704962 | SC4MOL | NM_001017369 | 0.0392 | 1.684 | 1.684 |
| NM_006536 | CLCA2 | NM_006536 | 0.0175 | 1.685 | 1.685 |
| BC001305 | ELOVL6 | NM_024090 | 0.0024 | 1.686 | 1.686 |
| AA708470 |  |  | 0.0408 | 1.687 | 1.687 |
| BC005247 | IDI1 | NM_004508 | 0.0015 | 1.688 | 1.688 |
| BC034316 |  |  | 0.0493 | 1.693 | 1.693 |
| AW298070 |  |  | 0.0297 | 1.695 | 1.695 |
| NM_002130 | HMGCS1 | NM_001098272 | 0.0067 | 1.696 | 1.696 |
| BC034248 | NBR2 | NM_005821 | 0.0048 | 1.699 | 1.699 |
| NM_173503 | EFCAB3 | NM_173503 | 0.0493 | 1.699 | 1.699 |
| AL136597 | KLHL7 | NM_001031710 | 0.0243 | 1.700 | 1.700 |
| AA779795 | TEF | NM_003216 | 0.0235 | 1.702 | 1.702 |
| BE645222 | ZSWIM7 | NM_001042697 | 0.0463 | 1.702 | 1.702 |
| NM_005896 | IDH1 | NM_005896 | 0.0263 | 1.702 | 1.702 |
| AA083483 | FTH1 | NM_002032 | 0.0124 | 1.702 | 1.702 |
| NM_005044 | PRKX /// PRKY | NM_002760 | 0.0147 | 1.703 | 1.703 |
| NM_022157 | RRAGC | NM_022157 | 0.0063 | 1.704 | 1.704 |
| AI972146 | LOC401577 | XM_379694 | 0.0440 | 1.705 | 1.705 |
| NM_005346 | HSPA1B | NM_005346 | 0.0397 | 1.705 | 1.705 |
| NM_024589 | ROGDI | NM_024589 | 0.0443 | 1.708 | 1.708 |
| L14611 | RORA | NM_002943 | 0.0062 | 1.709 | 1.709 |
| AA628398 | STARD4 | NM_139164 | 0.0394 | 1.709 | 1.709 |
| AF112204 | ATP6V1H | NM_015941 | 0.0223 | 1.712 | 1.712 |
| BC001727 | ANKRD10 | NM_017664 | 0.0275 | 1.713 | 1.713 |
| W93847 | MUC15 | NM_145650 | 0.0454 | 1.713 | 1.713 |
| NM_022912 | REEP1 | NM_022912 | 0.0006 | 1.716 | 1.716 |
| NM_003620 | PPM1D | NM_003620 | 0.0300 | 1.716 | 1.716 |
| AA886870 | ANKRD37 | NM_181726 | 0.0444 | 1.718 | 1.718 |
| AL359652 | LOC92497 | XM_931850 | 0.0187 | 1.718 | 1.718 |
| NM_144707 | PROM2 | NM_144707 | 0.0109 | 1.719 | 1.719 |
| CA313430 |  |  | 0.0239 | 1.723 | 1.723 |
| Y13786 | ADAM19 | NM_023038 | 0.0246 | 1.723 | 1.723 |
| AA284532 | C9orf19 | NM_022343 | 0.0268 | 1.723 | 1.723 |
| AI439556 | TXNIP | NM_006472 | 0.0024 | 1.725 | 1.725 |
| AV686514 | EMP2 | NM_001424 | 0.0176 | 1.726 | 1.726 |
| AW138827 | TAF5 | NM_006951 | 0.0122 | 1.726 | 1.726 |
| NM_006350 | FST | NM_006350 | 0.0146 | 1.727 | 1.727 |
| NM_024094 | DCC1 | NM_024094 | 0.0079 | 1.728 | 1.728 |
| AI863954 |  |  | 0.0142 | 1.730 | 1.730 |
| AF216962 | CNNM2 | NM_017649 | 0.0478 | 1.733 | 1.733 |
| BE550599 | CACNA1D | NM_000720 | 0.0163 | 1.735 | 1.735 |
| AI305170 | SLC25A16 | NM_152707 | 0.0394 | 1.740 | 1.740 |
| AB037791 | KIAA1370 | NM_019600 | 0.0008 | 1.741 | 1.741 |
| AI923944 |  |  | 0.0119 | 1.741 | 1.741 |
| AW241813 | H2AFJ | NM_018267 | 0.0220 | 1.744 | 1.744 |
| AA770596 | MARCKS | NM_002356 | 0.0297 | 1.746 | 1.746 |
| AI273692 |  |  | 0.0378 | 1.746 | 1.746 |
| AI671172 | TMEM68 | NM_152417 | 0.0270 | 1.746 | 1.746 |
| NM_003864 | SAP30 | NM_003864 | 0.0079 | 1.747 | 1.747 |
| AF116709 | GAPDH |  | 0.0405 | 1.749 | 1.749 |
| AI932618 |  |  | 0.0490 | 1.752 | 1.752 |
| AL049215 | DST | NM_001723 | 0.0486 | 1.752 | 1.752 |
| AI810767 |  |  | 0.0320 | 1.754 | 1.754 |
| AI361034 |  |  | 0.0243 | 1.755 | 1.755 |
| BF589251 |  |  | 0.0178 | 1.755 | 1.755 |
| AI122770 | FBXL20 | NM_032875 | 0.0255 | 1.756 | 1.756 |
| NM_001458 | FLNC | NM_001458 | 0.0155 | 1.763 | 1.763 |
| AL524643 | TMEM198 | NM_001005209 | 0.0180 | 1.763 | 1.763 |
| AK002152 | STAU2 | NM_014393 | 0.0053 | 1.769 | 1.769 |
| NM_024578 | OCEL1 | NM_024578 | 0.0011 | 1.773 | 1.773 |
| AI925316 |  |  | 0.0327 | 1.774 | 1.774 |
| NM_017729 | EPS8L1 | NM_017729 | 0.0066 | 1.779 | 1.779 |
| AI632214 |  |  | 0.0190 | 1.779 | 1.779 |
| NM_017818 | WDR8 | NM_017818 | 0.0492 | 1.780 | 1.780 |
| AL138431 | MTHFR | NM_005957 | 0.0210 | 1.780 | 1.780 |
| AK023754 | HES2 | NM_019089 | 0.0414 | 1.784 | 1.784 |
| AU145356 | AGPAT5 | NM_018361 | 0.0261 | 1.785 | 1.785 |
| AI146450 | NANP | NM_152667 | 0.0083 | 1.792 | 1.792 |
| AI817041 | CXCR7 | NM_020311 | 0.0477 | 1.797 | 1.797 |
| BC001282 | HMGN4 | NM_006353 | 0.0042 | 1.800 | 1.800 |
| AI743092 |  |  | 0.0068 | 1.801 | 1.801 |
| AI141670 | FAM131A | NM_144635 | 0.0022 | 1.803 | 1.803 |
| AI810669 |  |  | 0.0191 | 1.805 | 1.805 |
| NM_018370 | DRAM | NM_018370 | 0.0067 | 1.809 | 1.809 |
| M68956 | MARCKS | NM_002356 | 0.0457 | 1.814 | 1.814 |
| NM_019081 | KIAA0430 | NM_014647 | 0.0222 | 1.815 | 1.815 |
| AA649070 | DKFZp667E0512 |  | 0.0266 | 1.820 | 1.820 |
| NM_004403 | DFNA5 | NM_004403 | 0.0005 | 1.822 | 1.822 |
| AA551090 | AP1S2 | NM_003916 | 0.0004 | 1.825 | 1.825 |
| AL136820 | FAM135A | NM_001105531 | 0.0089 | 1.826 | 1.826 |
| AB040875 | SLC7A11 | NM_014331 | 0.0195 | 1.827 | 1.827 |
| AI028528 |  |  | 0.0485 | 1.828 | 1.828 |
| AF251050 | TIGD7 | NM_033208 | 0.0259 | 1.829 | 1.829 |
| BF516341 |  |  | 0.0002 | 1.833 | 1.833 |
| AA702248 | UCA1 |  | 0.0285 | 1.837 | 1.837 |
| NM_014454 | SESN1 | NM_014454 | 0.0196 | 1.841 | 1.841 |
| NM_015385 | SORBS1 | NM_001034954 | 0.0446 | 1.842 | 1.842 |
| NM_004772 | C5orf13 | NM_004772 | 0.0345 | 1.845 | 1.845 |
| NM_018267 | H2AFJ | NM_018267 | 0.0263 | 1.846 | 1.846 |
| BC016828 | ASAH1 | NM_004315 | 0.0222 | 1.848 | 1.848 |
| BC043594 | TCTE3 | NM_174910 | 0.0111 | 1.850 | 1.850 |
| AI991328 | CHKA | NM_001277 | 0.0023 | 1.851 | 1.851 |
| BC005202 | NIPSNAP3B | NM_018376 | 0.0488 | 1.852 | 1.852 |
| AA573901 | CCDC57 /// LOC732476 | NM_198082 | 0.0211 | 1.854 | 1.854 |
| NM_015515 | KRT23 | NM_015515 | 0.0106 | 1.855 | 1.855 |
| AL132665 | BNIP3L | NM_004331 | 0.0221 | 1.859 | 1.859 |
| BC003073 | ARHGEF10L | NM_001011722 | 0.0013 | 1.859 | 1.859 |
| AV703731 |  |  | 0.0045 | 1.864 | 1.864 |
| AV648364 | CBX7 | NM_175709 | 0.0482 | 1.872 | 1.872 |
| AL558164 | TMEM143 | NM_018273 | 0.0417 | 1.872 | 1.872 |
| AI803010 |  |  | 0.0062 | 1.875 | 1.875 |
| AI014470 | LOC728485 | XM_001130518 | 0.0015 | 1.876 | 1.876 |
| NM_005689 | ABCB6 | NM_005689 | 0.0020 | 1.876 | 1.876 |
| NM_024090 | ELOVL6 | NM_024090 | 0.0218 | 1.877 | 1.877 |
| AI761748 | NCOA3 | NM_006534 | 0.0002 | 1.881 | 1.881 |
| BE858194 |  |  | 0.0198 | 1.883 | 1.883 |
| AI538394 | NSUN7 | NM_024677 | 0.0067 | 1.889 | 1.889 |
| NM_014155 | ZBTB44 | NM_014155 | 0.0420 | 1.890 | 1.890 |
| NM_004508 | IDI1 | NM_004508 | 0.0061 | 1.891 | 1.891 |
| AK001947 | RP5-1022P6.2 | NM_019593 | 0.0041 | 1.894 | 1.894 |
| AF019214 | HBP1 | NM_012257 | 0.0039 | 1.906 | 1.906 |
| NM_000389 | CDKN1A | NM_000389 | 0.0138 | 1.906 | 1.906 |
| H27948 | MGC33894 | NM_152914 | 0.0040 | 1.907 | 1.907 |
| BF569051 | H19 | NR_002196 | 0.0146 | 1.911 | 1.911 |
| NM_004849 | ATG5 | NM_004849 | 0.0373 | 1.913 | 1.913 |
| AW241910 | COL22A1 | NM_152888 | 0.0304 | 1.915 | 1.915 |
| NM_006763 | BTG2 | NM_006763 | 0.0101 | 1.917 | 1.917 |
| AA401492 | GNAS | NM_000516 | 0.0268 | 1.917 | 1.917 |
| NM_024702 | ZNF750 | NM_024702 | 0.0135 | 1.921 | 1.921 |
| AA776810 | ZNF610 | NM_173530 | 0.0027 | 1.926 | 1.926 |
| AI758317 |  |  | 0.0149 | 1.926 | 1.926 |
| AI817264 | SP6 | NM_199262 | 0.0056 | 1.931 | 1.931 |
| AI242583 | MYCT1 | NM_025107 | 0.0164 | 1.931 | 1.931 |
| BC039154 | C16orf79 | NM_182563 | 0.0281 | 1.932 | 1.932 |
| AI817388 | GNPDA2 | NM_138335 | 0.0288 | 1.935 | 1.935 |
| NM_018593 | SLC16A10 | NM_018593 | 0.0195 | 1.935 | 1.935 |
| AF147782 | ETV7 | NM_016135 | 0.0035 | 1.937 | 1.937 |
| BC024748 |  |  | 0.0258 | 1.937 | 1.937 |
| AL133001 | SULF2 | NM_018837 | 0.0050 | 1.938 | 1.938 |
| BG031897 | AMN1 | NM_207337 | 0.0221 | 1.938 | 1.938 |
| AI553933 | SLC30A1 | NM_021194 | 0.0173 | 1.940 | 1.940 |
| BC003177 | CALCOCO1 | NM_020898 | 0.0442 | 1.947 | 1.947 |
| AI738556 | TNFRSF10D | NM_003840 | 0.0050 | 1.947 | 1.947 |
| AW006935 | ATP10B | NM_025153 | 0.0351 | 1.948 | 1.948 |
| AI188653 | MXD1 | NM_002357 | 0.0066 | 1.952 | 1.952 |
| H63435 | C11orf54 | NM_014039 | 0.0094 | 1.959 | 1.959 |
| AW235548 | MYO5A | NM_000259 | 0.0073 | 1.963 | 1.963 |
| NM_003234 | TFRC | NM_003234 | 0.0001 | 1.964 | 1.964 |
| AA502768 | C5orf34 | NM_198566 | 0.0284 | 1.968 | 1.968 |
| BE540552 | FADS1 | NM_013402 | 0.0037 | 1.975 | 1.975 |
| NM_018050 | MANSC1 | NM_018050 | 0.0125 | 1.979 | 1.979 |
| NM_003151 | STAT4 | NM_003151 | 0.0167 | 1.986 | 1.986 |
| AA669336 | COCH | NM_004086 | 0.0074 | 1.989 | 1.989 |
| NM_014398 | LAMP3 | NM_014398 | 0.0242 | 2.002 | 2.002 |
| BF001786 | SCML1 | NM_001037535 | 0.0306 | 2.004 | 2.004 |
| BF438173 | FST | NM_006350 | 0.0180 | 2.009 | 2.009 |
| AA811371 |  |  | 0.0441 | 2.014 | 2.014 |
| NM_025001 | MTHFD2L | NM_001004346 | 0.0021 | 2.016 | 2.016 |
| BC040700 |  |  | 0.0292 | 2.020 | 2.020 |
| AL042588 | PEG3 | NM_006210 | 0.0207 | 2.022 | 2.022 |
| AI440495 | LOC284702 |  | 0.0492 | 2.023 | 2.023 |
| AI934569 | ASAH1 | NM_004315 | 0.0110 | 2.025 | 2.025 |
| BE513006 | PROM2 | NM_144707 | 0.0115 | 2.026 | 2.026 |
| M76742 | CEACAM1 | NM_001024912 | 0.0088 | 2.026 | 2.026 |
| AL571684 | LOC401152 | NM_001001701 | 0.0158 | 2.029 | 2.029 |
| AK096683 | ZNF33B | NM_006955 | 0.0035 | 2.036 | 2.036 |
| AL136680 | GBP3 | NM_018284 | 0.0265 | 2.040 | 2.040 |
| AA135522 | GPD1L | NM_015141 | 0.0063 | 2.045 | 2.045 |
| BF970044 |  |  | 0.0035 | 2.047 | 2.047 |
| U47674 | ASAH1 | NM_004315 | 0.0424 | 2.051 | 2.051 |
| BG165833 | FADS1 | NM_013402 | 0.0002 | 2.052 | 2.052 |
| AA088177 | TMEM200A | NM_052913 | 0.0111 | 2.052 | 2.052 |
| BF063271 | GALNT3 | NM_004482 | 0.0028 | 2.058 | 2.058 |
| AI075407 | IFIT3 | NM_001031683 | 0.0080 | 2.060 | 2.060 |
| AI004453 | TRIML1 | NM_178556 | 0.0385 | 2.062 | 2.062 |
| NM_024519 | FAM65A | NM_024519 | 0.0264 | 2.062 | 2.062 |
| AK095151 | UBR5 | NM_015902 | 0.0068 | 2.065 | 2.065 |
| N49935 | RASSF4 | NM_032023 | 0.0143 | 2.067 | 2.067 |
| AF070673 | SNN | NM_003498 | 0.0088 | 2.068 | 2.068 |
| AI432195 |  |  | 0.0059 | 2.069 | 2.069 |
| AK026736 | ITGB6 |  | 0.0014 | 2.072 | 2.072 |
| AF131801 | SPG3A | NM_015915 | 0.0057 | 2.077 | 2.077 |
| AB051846 | RAP1A | NM_001010935 | 0.0397 | 2.080 | 2.080 |
| NM_016323 | HERC5 | NM_016323 | 0.0007 | 2.083 | 2.083 |
| AW237462 | MAP7D2 | NM_152780 | 0.0325 | 2.089 | 2.089 |
| N21320 | SLC12A6 | NM_001042494 | 0.0293 | 2.089 | 2.089 |
| N74607 | AQP3 | NM_004925 | 0.0333 | 2.089 | 2.089 |
| AB037810 | SIPA1L2 | NM_020808 | 0.0017 | 2.094 | 2.094 |
| AI650582 | FAM118A | NM_001104595 | 0.0204 | 2.105 | 2.105 |
| AA946876 |  |  | 0.0363 | 2.109 | 2.109 |
| NM_003823 | RTEL1 /// TNFRSF6B | NM_003823 | 0.0476 | 2.109 | 2.109 |
| BC032952 | MEX3C | NM_016626 | 0.0319 | 2.111 | 2.111 |
| AL574184 | HPGD | NM_000860 | 0.0446 | 2.112 | 2.112 |
| AF280094 | SP110 | NM_004509 | 0.0318 | 2.113 | 2.113 |
| BC004907 | EPS8L1 | NM_017729 | 0.0199 | 2.115 | 2.115 |
| AA166895 | NHLH2 | NM_001111061 | 0.0005 | 2.126 | 2.126 |
| AB037797 | ARRDC3 | NM_020801 | 0.0161 | 2.129 | 2.129 |
| AW511227 | MIB2 | NM_080875 | 0.0425 | 2.131 | 2.131 |
| S69232 | ETFDH | NM_004453 | 0.0349 | 2.134 | 2.134 |
| N22849 |  |  | 0.0074 | 2.140 | 2.140 |
| AW611550 | MFSD8 | NM_152778 | 0.0030 | 2.145 | 2.145 |
| AI884906 | RNF182 | NM_152737 | 0.0041 | 2.146 | 2.146 |
| AI743534 | ARHGAP24 | NM_001025616 | 0.0074 | 2.154 | 2.154 |
| AL117607 | LOC203274 |  | 0.0165 | 2.155 | 2.155 |
| NM_001277 | CHKA /// LOC650122 | NM_001277 | 0.0192 | 2.157 | 2.157 |
| BC020812 | LOC389072 | NM_001080475 | 0.0197 | 2.160 | 2.160 |
| NM_005410 | SEPP1 | NM_001085486 | 0.0145 | 2.163 | 2.163 |
| U46006 | CSRP2 | NM_001321 | 0.0215 | 2.170 | 2.170 |
| AL120021 | KLHL24 | NM_017644 | 0.0190 | 2.175 | 2.175 |
| BF512388 | C10orf58 | NM_032333 | 0.0004 | 2.176 | 2.176 |
| NM_024581 | C6orf60 | NM_001100411 | 0.0016 | 2.193 | 2.193 |
| NM_017786 | GOLSYN | NM_001099743 | 0.0069 | 2.193 | 2.193 |
| NM_013409 | FST | NM_006350 | 0.0341 | 2.195 | 2.195 |
| R12665 | PATL2 | XR_015470 | 0.0163 | 2.197 | 2.197 |
| AL109698 |  |  | 0.0190 | 2.204 | 2.204 |
| AW402635 | POLR2J2 /// POLR2J3 /// POLR2J4 | NM_001015884 | 0.0271 | 2.206 | 2.206 |
| U77914 | JAG1 | NM_000214 | 0.0077 | 2.207 | 2.207 |
| AL512760 | FADS1 | NM_013402 | 0.0121 | 2.214 | 2.214 |
| AW071793 | MXD1 | NM_002357 | 0.0104 | 2.220 | 2.220 |
| NM_004509 | SP110 | NM_004509 | 0.0068 | 2.223 | 2.223 |
| BC030754 |  |  | 0.0255 | 2.228 | 2.228 |
| AI435399 | SLFN5 | NM_144975 | 0.0187 | 2.229 | 2.229 |
| AW204518 | ZNF341 | NM_032819 | 0.0229 | 2.237 | 2.237 |
| NM_020632 | ATP6V0A4 | NM_020632 | 0.0251 | 2.255 | 2.255 |
| W73230 | C7orf41 | NM_152793 | 0.0143 | 2.262 | 2.262 |
| AI991103 | C5orf39 | NM_001014279 | 0.0081 | 2.279 | 2.279 |
| AK022852 | SIPA1L2 | NM_020808 | 0.0001 | 2.280 | 2.280 |
| AA860341 | MORN3 | NM_173855 | 0.0308 | 2.284 | 2.284 |
| BF109592 | C11orf54 | NM_014039 | 0.0245 | 2.286 | 2.286 |
| BC006472 | DCAKD | NM_024819 | 0.0351 | 2.293 | 2.293 |
| NM_003813 | ADAM21 | NM_003813 | 0.0198 | 2.305 | 2.305 |
| AU157271 | LOC731450 | XM_001133142 | 0.0057 | 2.315 | 2.315 |
| U73936 | JAG1 | NM_000214 | 0.0101 | 2.319 | 2.319 |
| AJ247087 | MLCK | NM_182493 | 0.0061 | 2.321 | 2.321 |
| BC005871 | C10orf58 | NM_032333 | 0.0113 | 2.327 | 2.327 |
| AW301218 | THAP9 | NM_024672 | 0.0067 | 2.336 | 2.336 |
| AI822125 | DUSP27 | NM_001080426 | 0.0013 | 2.343 | 2.343 |
| NM_001321 | CSRP2 | NM_001321 | 0.0127 | 2.347 | 2.347 |
| AA565499 | NLRP7 | NM_139176 | 0.0151 | 2.347 | 2.347 |
| AW162015 | ZNF143 | NM_003442 | 0.0204 | 2.352 | 2.352 |
| AW134535 | CCNG2 | NM_004354 | 0.0120 | 2.357 | 2.357 |
| AA543084 |  |  | 0.0263 | 2.357 | 2.357 |
| AL575306 | H19 | NR_002196 | 0.0296 | 2.400 | 2.400 |
| N47725 | IFIT5 | NM_012420 | 0.0407 | 2.429 | 2.429 |
| NM_006536 | CLCA2 | NM_006536 | 0.0276 | 2.450 | 2.450 |
| AA400206 | FAM65A | NM_024519 | 0.0033 | 2.454 | 2.454 |
| AA131041 | IFIT2 | NM_001547 | 0.0418 | 2.456 | 2.456 |
| AW293316 |  |  | 0.0081 | 2.469 | 2.469 |
| AB046817 | SYTL2 | NM_032379 | 0.0114 | 2.469 | 2.469 |
| AV716964 | ATF7IP2 | NM_024997 | 0.0262 | 2.484 | 2.484 |
| NM_002356 | MARCKS | NM_002356 | 0.0089 | 2.492 | 2.492 |
| AA485440 | SPHK2 | NM_020126 | 0.0334 | 2.506 | 2.506 |
| AI827820 | MBD2 | NM_003927 | 0.0034 | 2.525 | 2.525 |
| W57613 |  |  | 0.0318 | 2.532 | 2.532 |
| Z98884 | CAMTA1 | NM_015215 | 0.0026 | 2.542 | 2.542 |
| AI890761 | TMEM68 | NM_152417 | 0.0012 | 2.552 | 2.552 |
| NM_052889 | CASP1 /// COP1 | NM_001017534 | 0.0360 | 2.580 | 2.580 |
| AI686890 |  |  | 0.0042 | 2.604 | 2.604 |
| AW341649 | TP53INP1 | NM_033285 | 0.0189 | 2.610 | 2.610 |
| AB051846 | RAP1A | NM_001010935 | 0.0383 | 2.616 | 2.616 |
| BE552414 | TMEM52 | NM_178545 | 0.0152 | 2.633 | 2.633 |
| AI826268 | SLC25A29 | NM_001039355 | 0.0037 | 2.636 | 2.636 |
| NM_006472 | TXNIP | NM_006472 | 0.0087 | 2.636 | 2.636 |
| AA911561 |  |  | 0.0292 | 2.649 | 2.649 |
| BF002104 | GDAP1 | NM_001040875 | 0.0166 | 2.669 | 2.669 |
| AI928764 | LOC154761 |  | 0.0161 | 2.687 | 2.687 |
| NM_018095 | KBTBD4 /// PTPMT1 | NM_016506 | 0.0003 | 2.698 | 2.698 |
| BC005286 | EPM2A | NM_001018041 | 0.0158 | 2.723 | 2.723 |
| NM_004354 | CCNG2 | NM_004354 | 0.0359 | 2.762 | 2.762 |
| AI572938 |  |  | 0.0184 | 2.792 | 2.792 |
| NM_004780 | TCEAL1 | NM_001006639 | 0.0229 | 2.810 | 2.810 |
| AI827820 | MBD2 | NM_003927 | 0.0066 | 2.824 | 2.824 |
| AW474434 | TNFSF10 | NM_003810 | 0.0070 | 2.838 | 2.838 |
| NM_024786 | ZDHHC11 | NM_024786 | 0.0054 | 2.851 | 2.851 |
| D63807 | LSS | NM_001001438 | 0.0037 | 2.891 | 2.891 |
| AI348159 | REEP6 | NM_138393 | 0.0035 | 2.897 | 2.897 |
| AI446414 | KITLG | NM_000899 | 0.0126 | 2.929 | 2.929 |
| AL117598 |  |  | 0.0244 | 2.957 | 2.957 |
| BE268538 | DENND4A | NM_005848 | 0.0268 | 2.987 | 2.987 |
| NM_006746 | SCML1 | NM_001037535 | 0.0211 | 3.009 | 3.009 |
| NM_003810 | TNFSF10 | NM_003810 | 0.0005 | 3.048 | 3.048 |
| AI709406 | MARCKS | NM_002356 | 0.0011 | 3.101 | 3.101 |
| BF114815 | MLCK | NM_182493 | 0.0008 | 3.112 | 3.112 |
| NM_025155 | PAAF1 | NM_025155 | 0.0481 | 3.244 | 3.244 |
| L49506 | CCNG2 | NM_004354 | 0.0033 | 3.344 | 3.344 |
| AV720803 |  |  | 0.0125 | 3.346 | 3.346 |
| NM_005670 | EPM2A | NM_001018041 | 0.0017 | 3.372 | 3.372 |
| BC035640 | AP3B2 | NM_004644 | 0.0010 | 3.401 | 3.401 |
| AU156189 |  |  | 0.0037 | 3.580 | 3.580 |
| AF003934 | GDF15 | NM_004864 | 0.0073 | 3.594 | 3.594 |
| NM_024626 | VTCN1 | NM_024626 | 0.0000 | 3.603 | 3.603 |
| AI286239 | LOC440731 | XM_933693 | 0.0000 | 3.699 | 3.699 |
| NM_001717 | BNC1 | NM_001717 | 0.0480 | 3.836 | 3.836 |
| NM_024703 | SMPD3 | NM_018667 | 0.0133 | 4.093 | 4.093 |
| AI376549 | MLCK | NM_182493 | 0.0090 | 4.153 | 4.153 |
| AF007162 | CRYAB | NM_001885 | 0.0043 | 4.560 | 4.560 |
| AF267859 | ZDHHC11 | NM_024786 | 0.0242 | 4.590 | 4.590 |
| R99291 | IHPK3 | NM_054111 | 0.0001 | 4.665 | 4.665 |
