# Supplementary material for: Effect of proline rich 15-deficiency on trophoblast viability and survival
Source: PLoS One. 2017 Apr 5;12(4):e0174976. doi: 10.1371/journal.pone.0174976 (PMC5381842; doi:10.1371/journal.pone.0174976)
Supplement: S2 Table — (DOC) [file pone.0174976.s002.doc]

S2 Table. Primers used for qPCR analysis.

|  | **Gene** | **Accession Number** | **Forward (5'-->3')** | **Reverse (5'-->3')** | **Size (bp)** | **T_a_**  **(°C)** |
| --- | --- | --- | --- | --- | --- | --- |
|  | *CCDC88A* | NM_001135597 | CTC TGC CAG AAT GTA CCG AGA | ATT TAT CAG AAC GAG CAC GAG T | 221 | 57 |
|  | *CCND1* | NM_053056 | ACG AAG GTC TGC GCG TGT T | CCG CTG GCC ATG AAC TAC CT | 320 | 58 |
|  | *CCNG2* | NM_004354 | GAG CTG CCA ACG ATA CCT G | TCT AAG ATG GAA AGC ACA GTG | 172 | 58 |
|  | *CDK6* | NM_001145306 | CGA GTA GTG CAT CGC GAT CTA A | GGT CTT TGC CTA GTT CAT CGA T | 407 | 58 |
|  | *CDKN1A* | NM_000389 | CGA AGT CAG TTC CTT GTG GAG | CAT GGG TTC TGA CGG ACA T | 111 | 57 |
|  | *CRYAB* | NM_001885 | CAC CCA GCT GGT TTG ACA CT | TGA CAG AGA ACC TGT CCT TCT | 63 | 57 |
|  | *GDF15* | NM_004864 | CCG GAT ACT CAC GCC AGA | AGA GAT ACG CAG GTG CAG | 63 | 58 |
|  | *IGF1R* | NM_000875 | CTC AAA AGT TAT CTC CGG TCT | TTT GAC TGT GAA ATC TTC GGC TA | 192 | 57 |
|  | *IGFBP3* | NM_000598 | CAT CAT CAA GAA AGG GCA T | GCT GCC CAT ACT TAT CCA C | 293 | 57 |
|  | *JAG1* | NM_000214 | CAA ACC TTG TGT AAA CGC CAA | ACC ATT AAC CAA ATC CCG ACA | 157 | 58 |
|  | *LIFR* | NM_001127671 | CCC CAA CAT GAC TTG CGA CT | CTG TAT AGG CTC GCA AGA CCA | 497 | 58 |
|  | *MCAM* | NM_006500 | TCA AGG AGA GGA AGG TGT GG | ACT CGC TGT GGA TCT TGG TC | 136 | 58 |
|  | *MXD1* | NM_001202513 | GAC AGA AAA GCC GTT CAC C | CTC GTC AGA GTC GCT CAC A | 228 | 57 |
|  | *MYC* | NM_002467 | CCT ACC CTC TCA ACG ACA GC | CTC TGA CCT TTT GCC AGG AG | 247 | 58 |
|  | *OVOL2* | NM_021220 | CAC CTC AAG TGC CAC AAC CAG | TGT AGC CGC AAT CCT CGC AGA | 256 | 58 |
|  | *PRR15* | NM_175887.2 | CCA GAA GCC TGA TCT CTC CA | CCC TTT CTC CAC GTG GTC T | 229 | 60 |
|  | *PTEN* | NM_000314 | CAC CGC CAA ATT TAA TTG CAG | CCC CGA TGT AAT AAA TAT GCA CA | 198 | 57 |
|  | *PXN* | NM_001080855 | CTG AGC CTT CAC CCA CCG TA | CCG CTT AGG CTT CTC TTT CGT | 233 | 58 |
|  | *RPS15* | NM_001018 | TTC CGC AAG TTC ACC TAC C | CGG GCC GGC CAT GCT TTA CG | 361 | 60 |
|  | *SOCS2* | NM_003877 | TCT CTG CCA CCA TTT CGG ACA | GTC CAA TCT GAA TTT TCC GTC T | 452 | 58 |
|  | *TFPI2* | NM_006528 | TCT GCC AAT GTG ACT CGC TA | ATT CTA CTG GCA AAG CGA AG | 179 | 58 |
|  | *TNFSF10* | NM_001190942 | TAC GTG TAC TTT ACC AAC GAG | GAG TTG CCA CTT GAC TTG C | 150 | 60 |
|  | *TWIST1* | NM_000474 | TCA GCT ACG CCT TCT CGG TC | AGA AAG TCC ATA GTG ATG CCT T | 473 | 58 |
